# Supplementary material for: Glycosylation-modulated conformational diversity in neurotrophin receptors
Source: Biophys J. 2026 Apr 18;125(16):4407–19. doi: 10.1016/j.bpj.2026.04.017 (PMC13507394; doi:10.1016/j.bpj.2026.04.017)
Supplement: Supplementary file 1 [file mmc1.pdf]

Biophysical Journal, Volume *125*

Supplemental information

Glycosylation-modulated conformational diversity in neurotrophin receptors

Alexandros Tsengenes, Christina Athanasiou, and Rebecca C. Wade

Supporting Material

Glycosylation-Modulated Conformational Diversity in Neurotrophin Receptors

Alexandros Tsengenes[1,2,3]†, Christina Athanasiou[1,2,3]†, Rebecca C. Wade[1,2,4,5]

[1] Molecular and Cellular Modeling Group, Heidelberg Institute for Theoretical Studies (HITS), 69118 Heidelberg, Germany.

[2] Faculty of Biosciences, Heidelberg University, 69120 Heidelberg, Germany.

[3] Heidelberg Biosciences International Graduate School, Heidelberg University, 69120 Heidelberg, Germany.

[4] Center for Molecular Biology of Heidelberg University (ZMBH), DKFZ-ZMBH Alliance, 69120 Heidelberg, Germany.

[5] Interdisciplinary Center for Scientific Computing (IWR), Heidelberg University, 69120 Heidelberg, Germany.

Table S1: Summary of simulated systems with or without glycans.

System No	Protein system	Glycosylation state	Replicas	Time/Replica (ns)
1	p75 dimer – NT3 dimer	+	3	500
2		-	3	500
3	p75 monomer – NT3 dimer	+	3	500
4		-	3	500
5	p75 monomer	+	3	500
6		-	3	500
7	TrkA dimer – NGF dimer	+	6	500
8		-	6	500
9	TrkA monomer – NGF dimer	+	6	500
10		-	6	500
11	TrkA monomer	+	3	500
12		-	3	500
13	TrkB dimer – NT-4/5 dimer	+	6	500
14		-	6	500
15	TrkB monomer – NT-4/5 dimer	+	6	500
16		-	6	500
17	TrkB monomer	+	6	500
18		-	6	500

Table S2: N-glycosylation positions on TrkA and TrkB receptors. For TrkB, the exact glycosylation positions are known experimentally.[1,2] For TrkA, the glycosylation positions that are known from the PDB ID: 2IFG[3] crystal structure are shown with normal font, while the ones that were added based on comparison with TrkB positions are shown in bold letters.

TrkA	TrkB
**Asn 67**	Asn 67
Asn 95	Asn 95
Asn 121	Asn 121
Asn 188	Asn 178
**Asn 202**	Asn 205
-	Asn 241
**Asn 253**	Asn 254
Asn 262	-
Asn 281	Asn 280
-	Asn 338
Asn 358	-
**Asn 401**	Asn 412

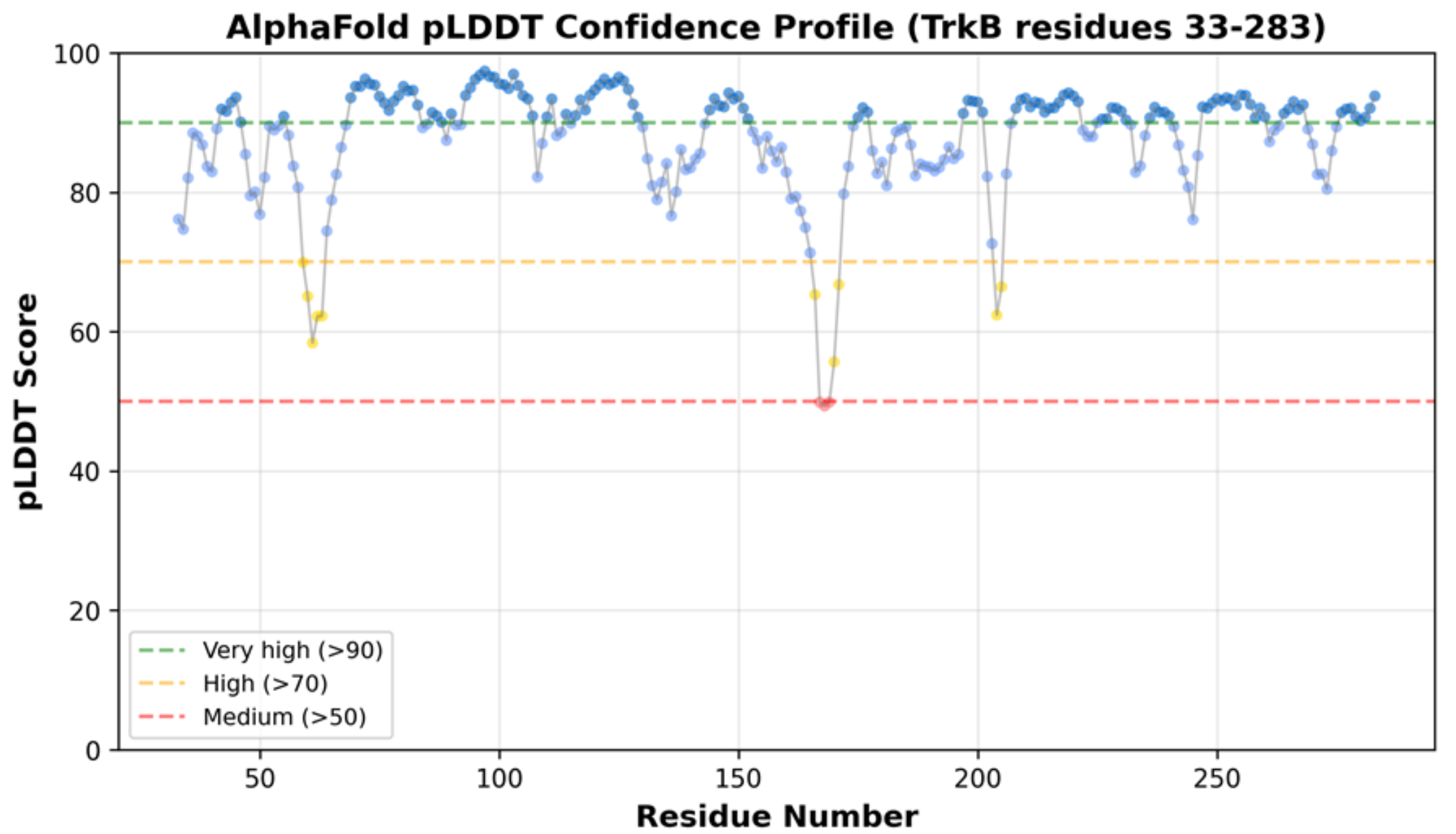

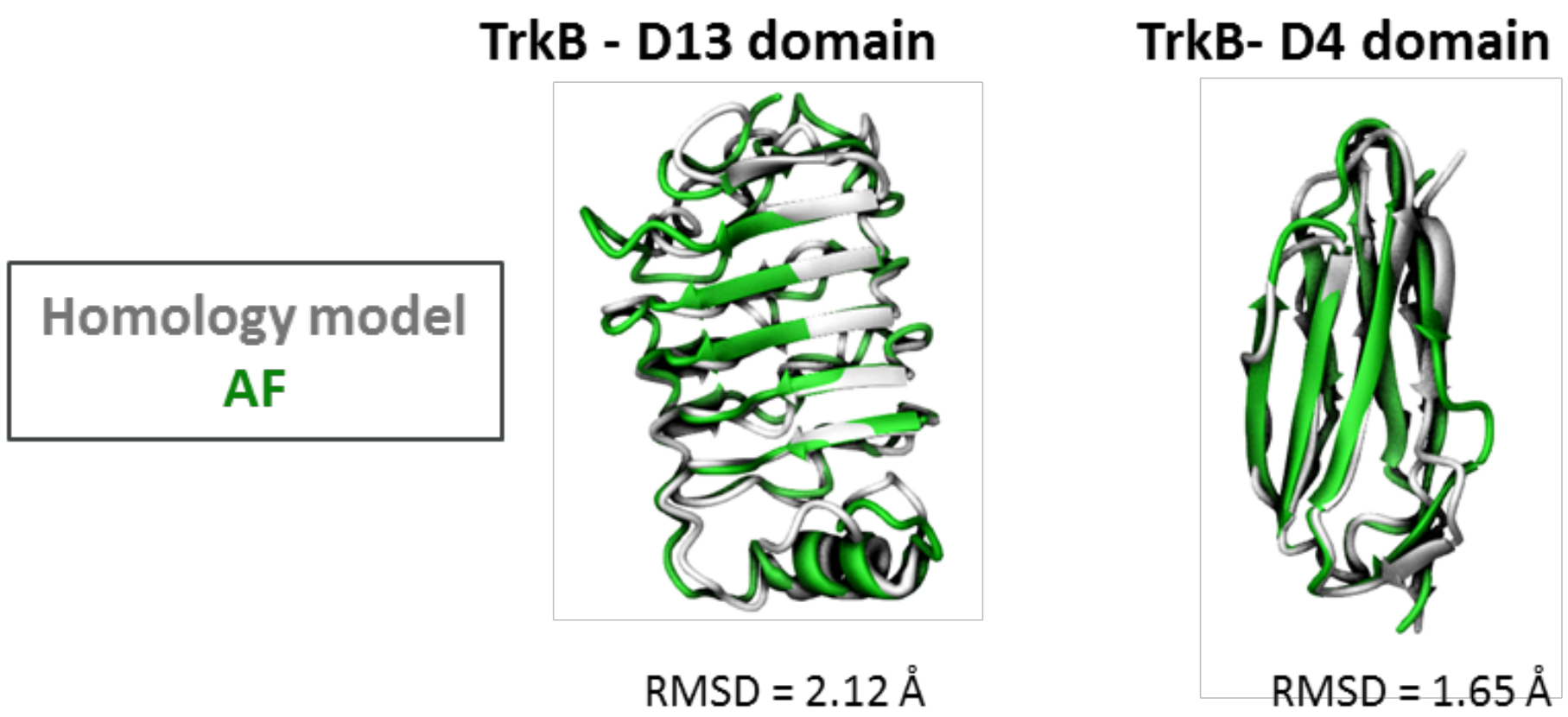

Figure S1: AlphaFold (AF) pLDDT score for the D1-D4 domains of TrkB (AF-Q16620-2-F1-v6 from the AlphaFold Protein Structure database (https://alphafold.ebi.ac.uk/)) and structural alignments of the D13 and D4 domains from the TrkB homology model used in the molecular dynamics simulations with the same domains from the AF model. Backbone RMSD values for the alignments are indicated below the figures.

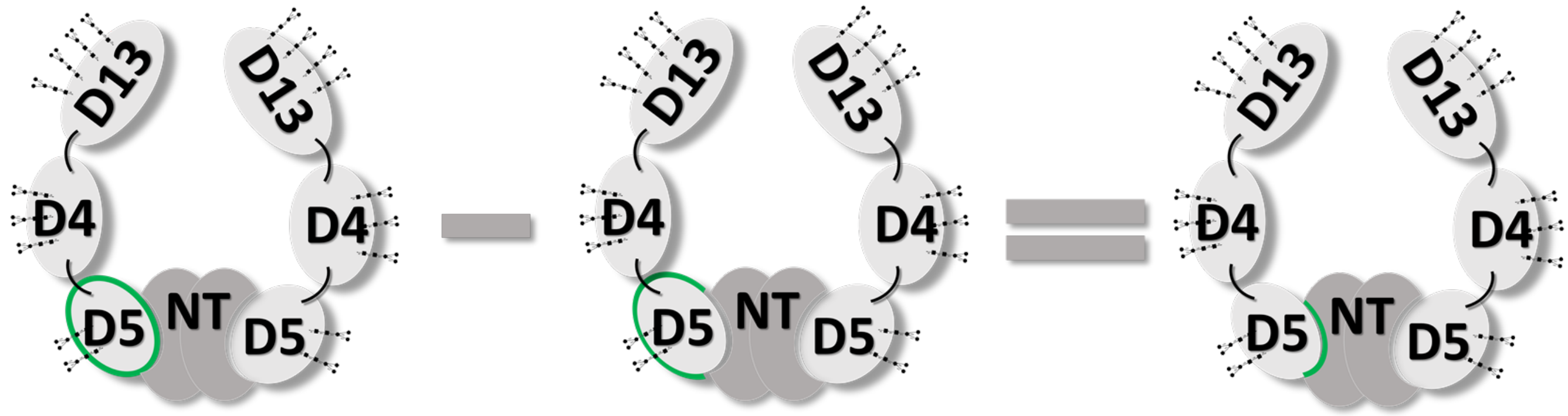

Figure S2: Schematic representation of the solvent accessible surface area (SASA) calculations that were performed to estimate the contact area of the EC domains of the receptors and the NTs. Here, the contact area between the D5 domain of the Trks and the NTs is shown as an example. For this purpose, the SASA of the D5 domain was calculated without and with the NT present and the difference of these two values yielded the contact area of D5-NT.

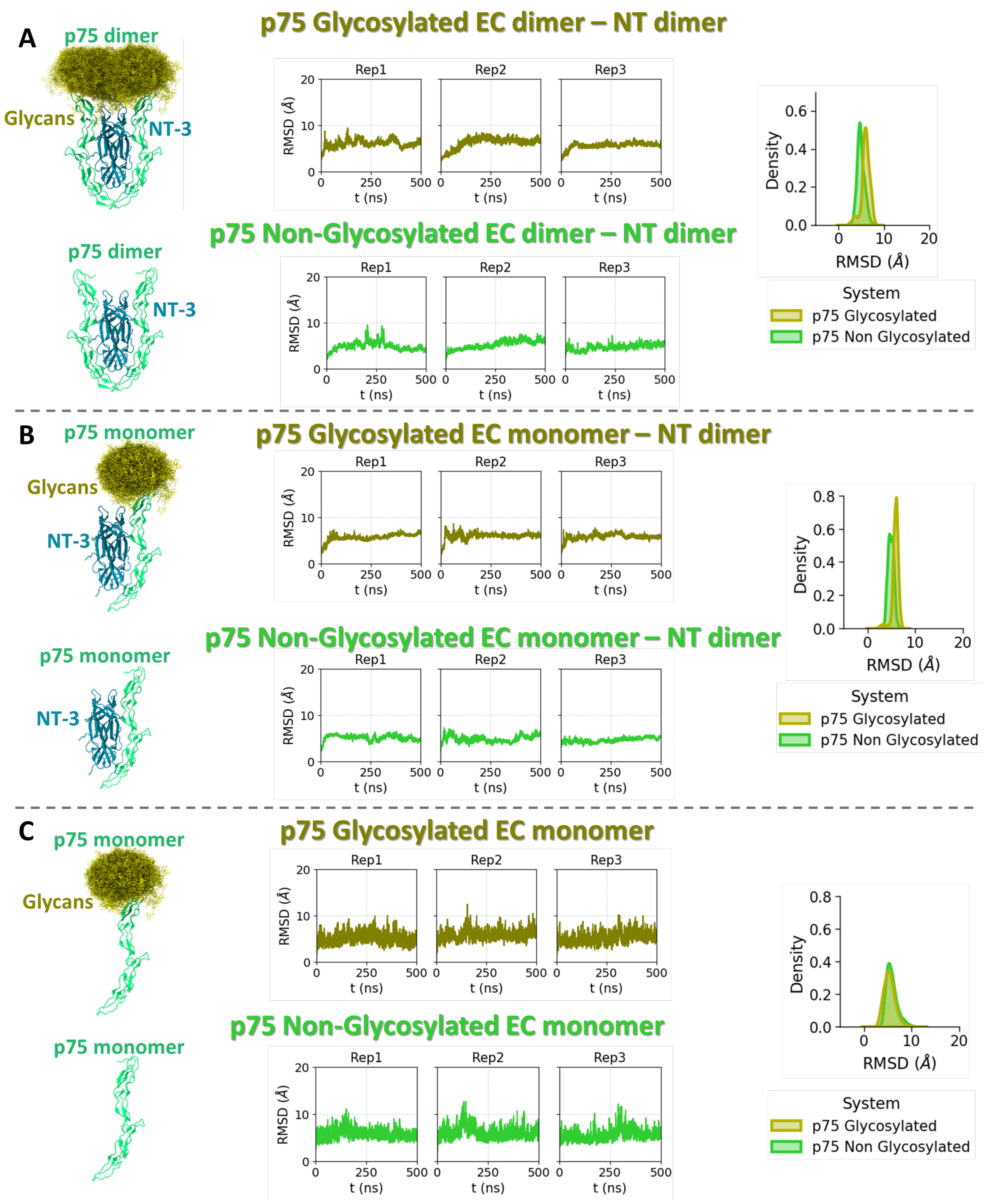

Figure S3: Summary of RMSD calculation results for the (A) p75-EC as a homodimer in complex to NT-3, (B) as a monomer in complex with NT-3 and (C) as a monomer alone, with and without glycans. The RMSD values are presented as an evolution with time (middle plots) and as probability densities for all the replicas together in the glycosylated and non-glycosylated systems (right plots).

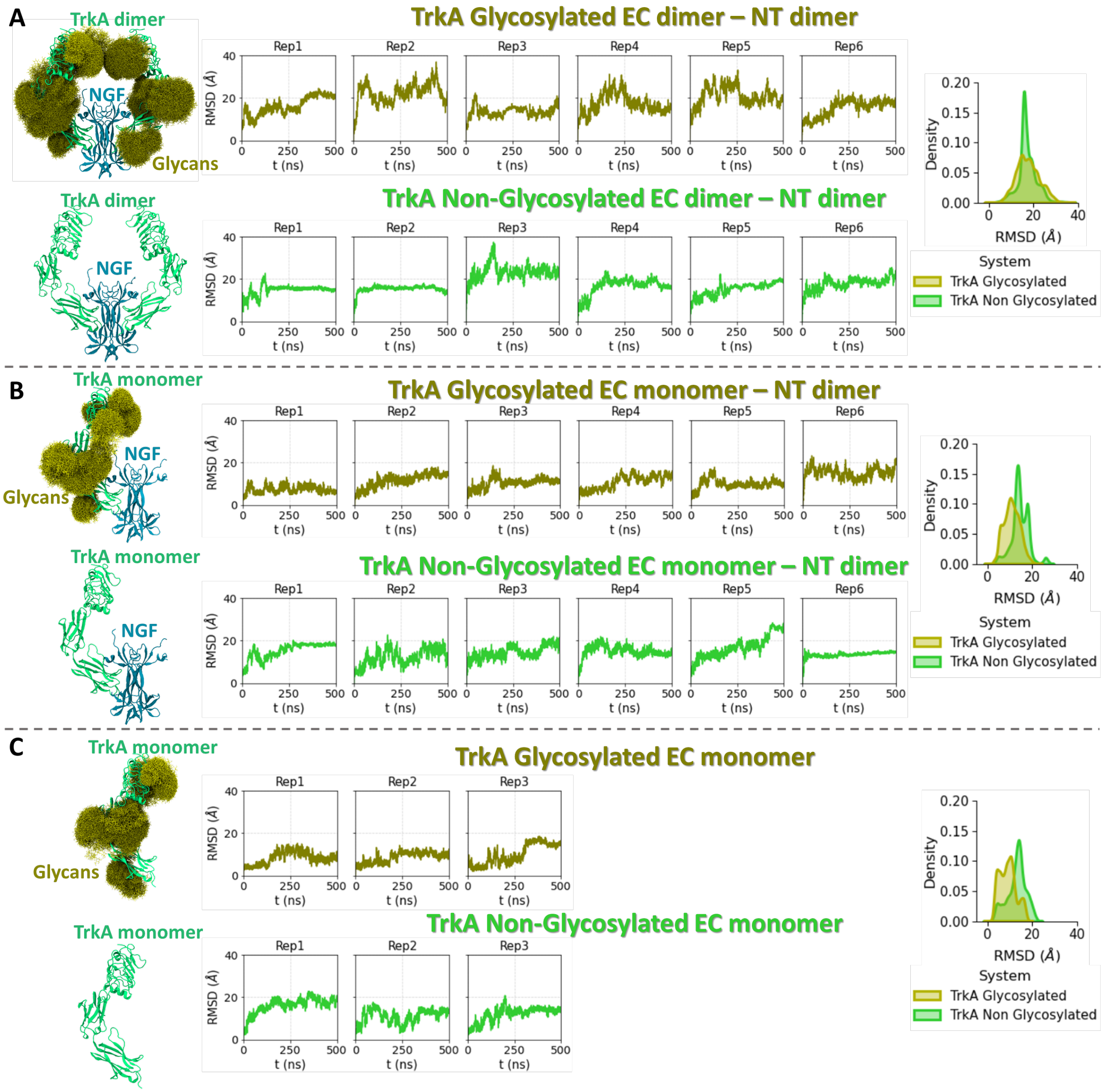

Figure S4: RMSD calculations of the glycosylated and non-glycosylated TrkA systems. (A), (B) and (C) show the RMSD for the TrkA-EC homodimer with the NGF homodimer bound, the TrkA-EC monomer with the NGF homodimer bound, the TrkA-EC monomer systems, respectively, with and without glycans. For each system the time-dependence of the RMSD of the protein backbone compared to the first frame (which corresponds to the crystal structure) is shown for each replica, as well as the distributions of RMSD values across all replicas and all frames of the production runs for the glycosylated and non-glycosylated systems (right plots in A, B and C).

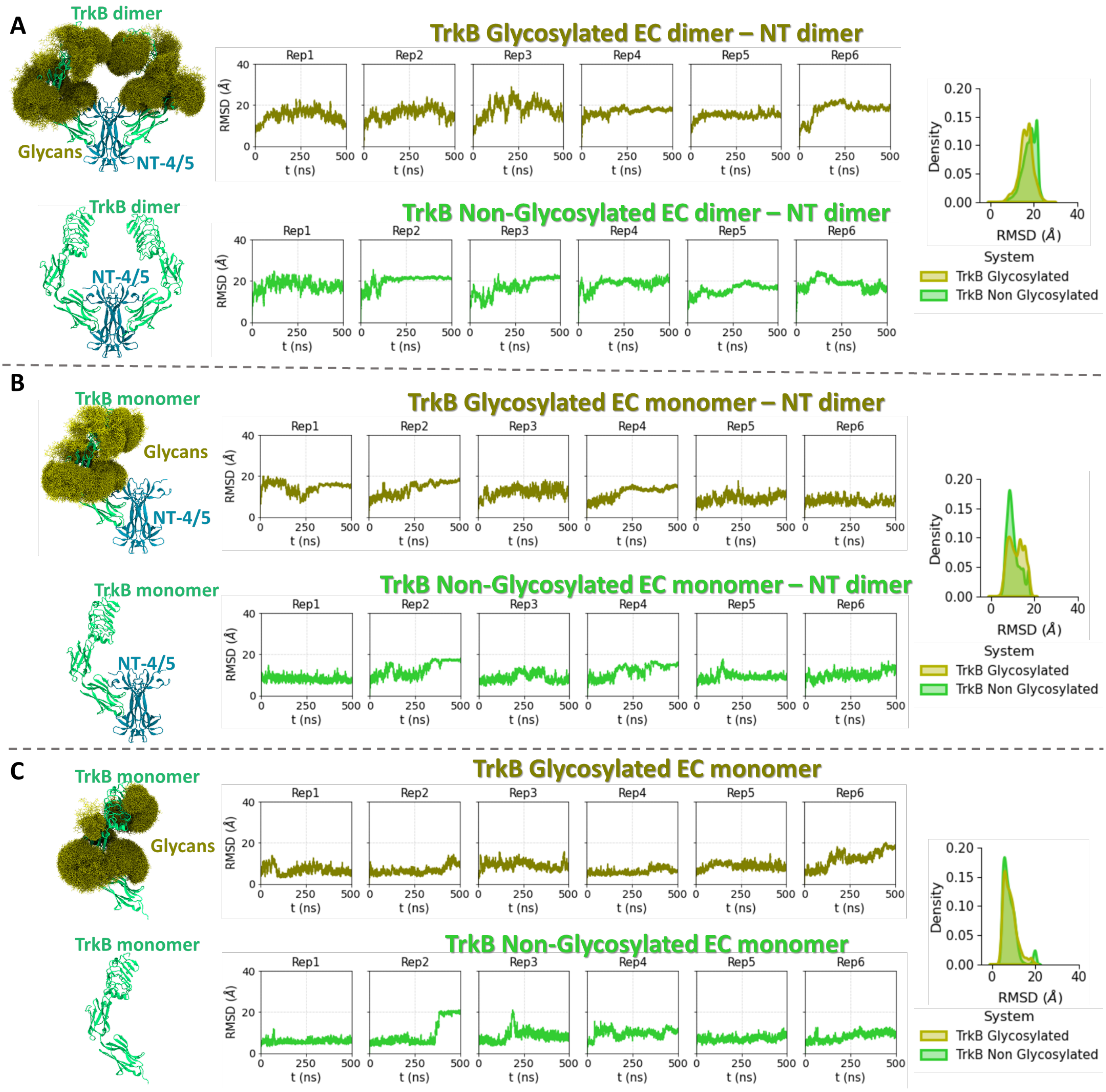

Figure S5: RMSD calculations of the glycosylated and non-glycosylated TrkB systems. (A), (B) and (C) show the RMSD for the TrkB-EC homodimer with the NT-4/5 homodimer bound, the TrkB-EC monomer with the NT-4/5 homodimer bound, the TrkB-EC monomer systems, respectively, with and without glycans. For each system the time-dependence of the RMSD of the protein backbone compared to the first frame (which corresponds to the crystal structure) is shown for each replica, as well as the distributions of RMSD values across all replicas and all frames of the production runs for the glycosylated and non-glycosylated systems (right plots in A, B and C).

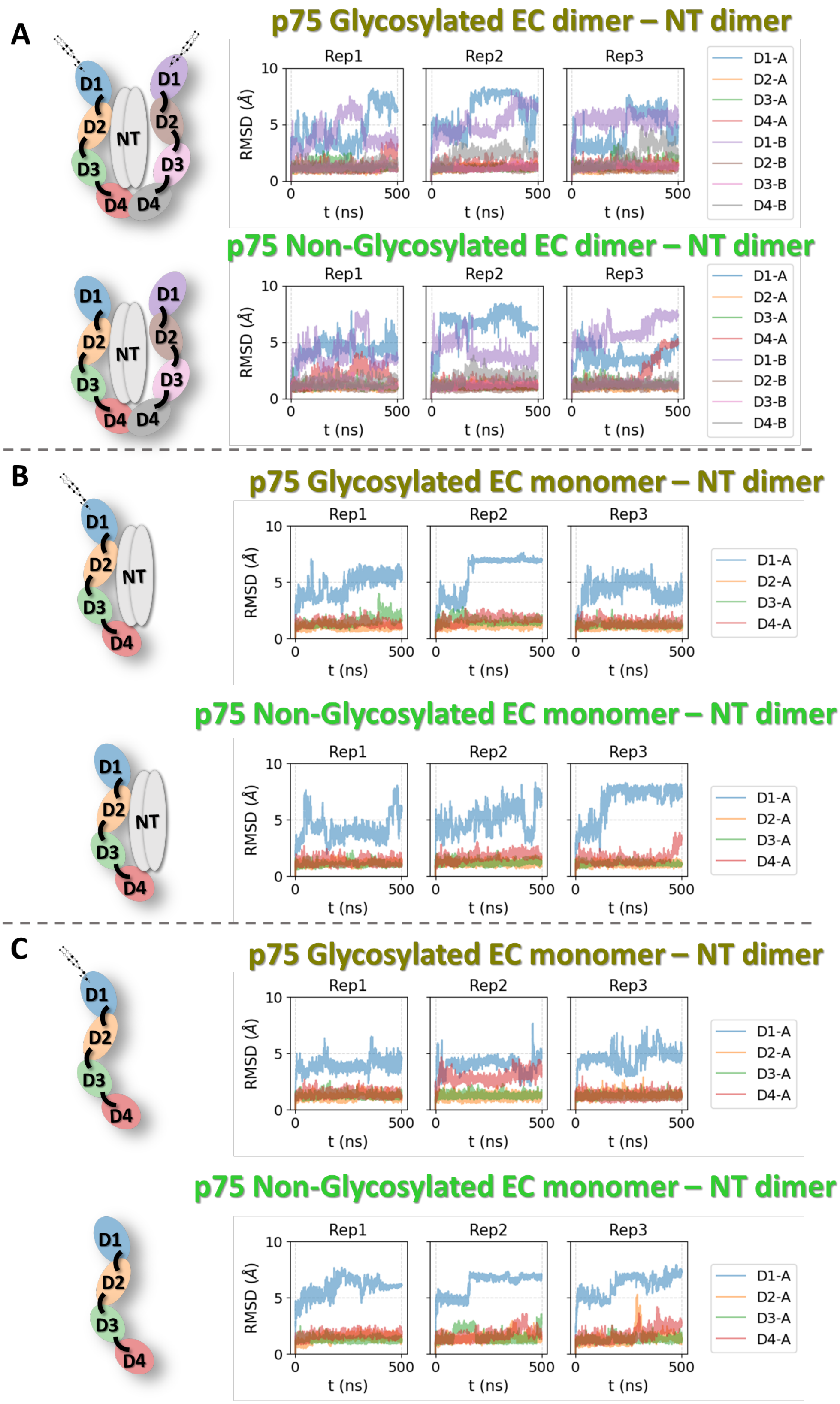

Figure S6: Summary of sub-domain RMSD calculation results for the (A) p75-EC as a homodimer in complex to NT-3, (B) as a monomer in complex with NT-3 and (C) as a monomer alone, with and without glycans. The RMSD values are presented as an evolution with time for each of the domains, after alignment to each domain.

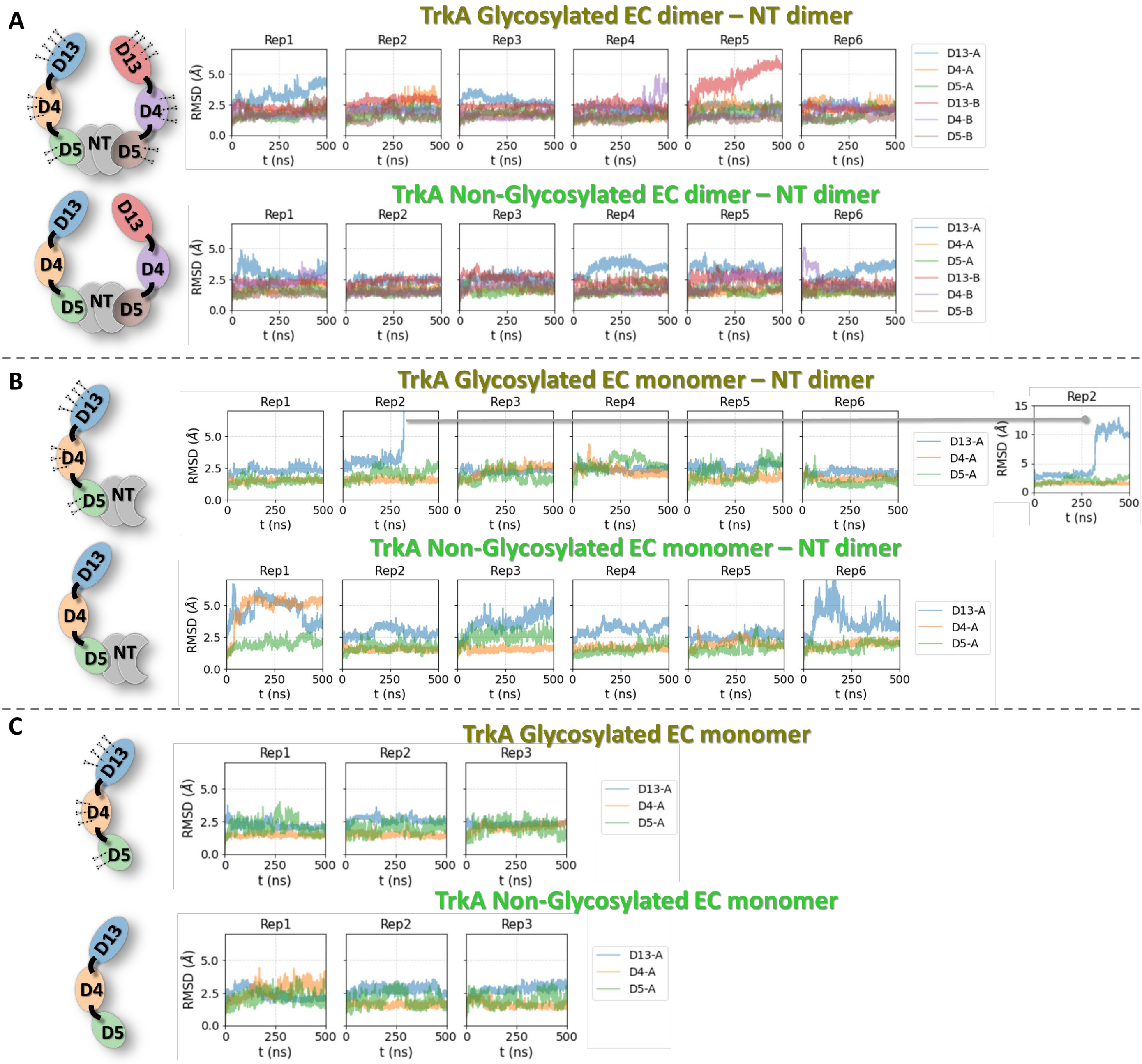

Figure S7: RMSD calculations for each domain of the glycosylated or not TrkA systems. (A), (B) and (C) show the internal RMSD for each domain the TrkA-EC homodimer with the NGF homodimer bound, the TrkA-EC monomer with the NGF homodimer bound, the TrkA-EC monomer systems, respectively, with and without glycans. For each system, the time-dependent RMSD of the domain backbone compared to the first frame is shown for each replica. Before the calculation of the internal RMSD of each domain, I performed alignment of the respective domain at each simulation snapshot. Schematic representation of each system, with color-coding of the domains is shown at the left. The first and second subunits are indicated with A and B letters in the legends.

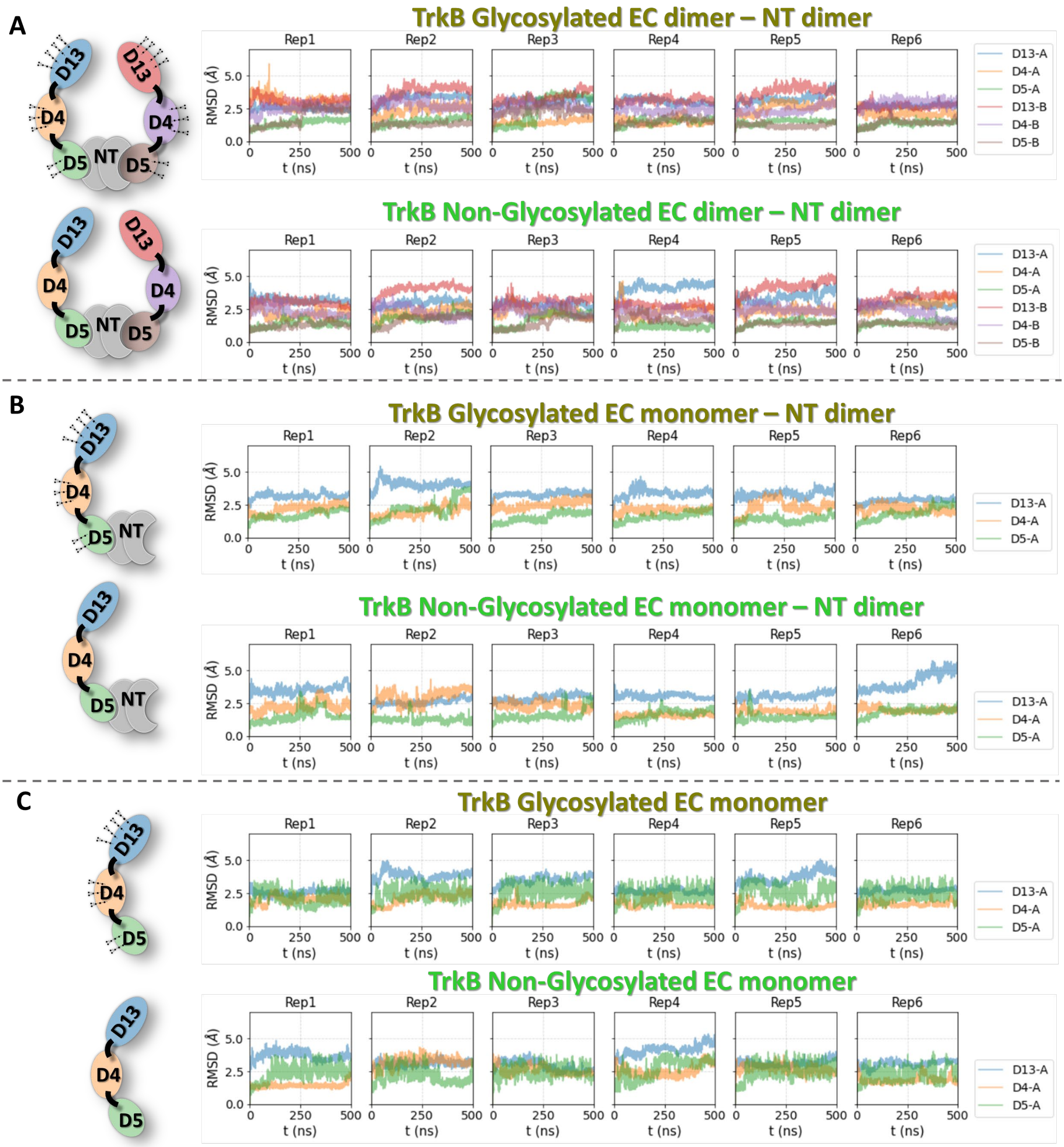

Figure S8: RMSD calculations for each domain of the glycosylated or not TrkB systems. (A), (B) and (C) show the internal RMSD for each domain the TrkB-EC homodimer with the NT-4/5 homodimer bound, the TrkB-EC monomer with the NT-4/5 homodimer bound, the TrkB-EC monomer systems, respectively, with and without glycans. For each system, the time-dependent RMSD of the domain backbone compared to the first frame is shown for each replica. Before the calculation of the internal RMSD of each domain, I performed alignment in the respective domain at each simulation snapshot. Schematic representation of each system, with color-coding of the domains is shown at the left. The first and second monomers are indicated with A and B letters in the legends.

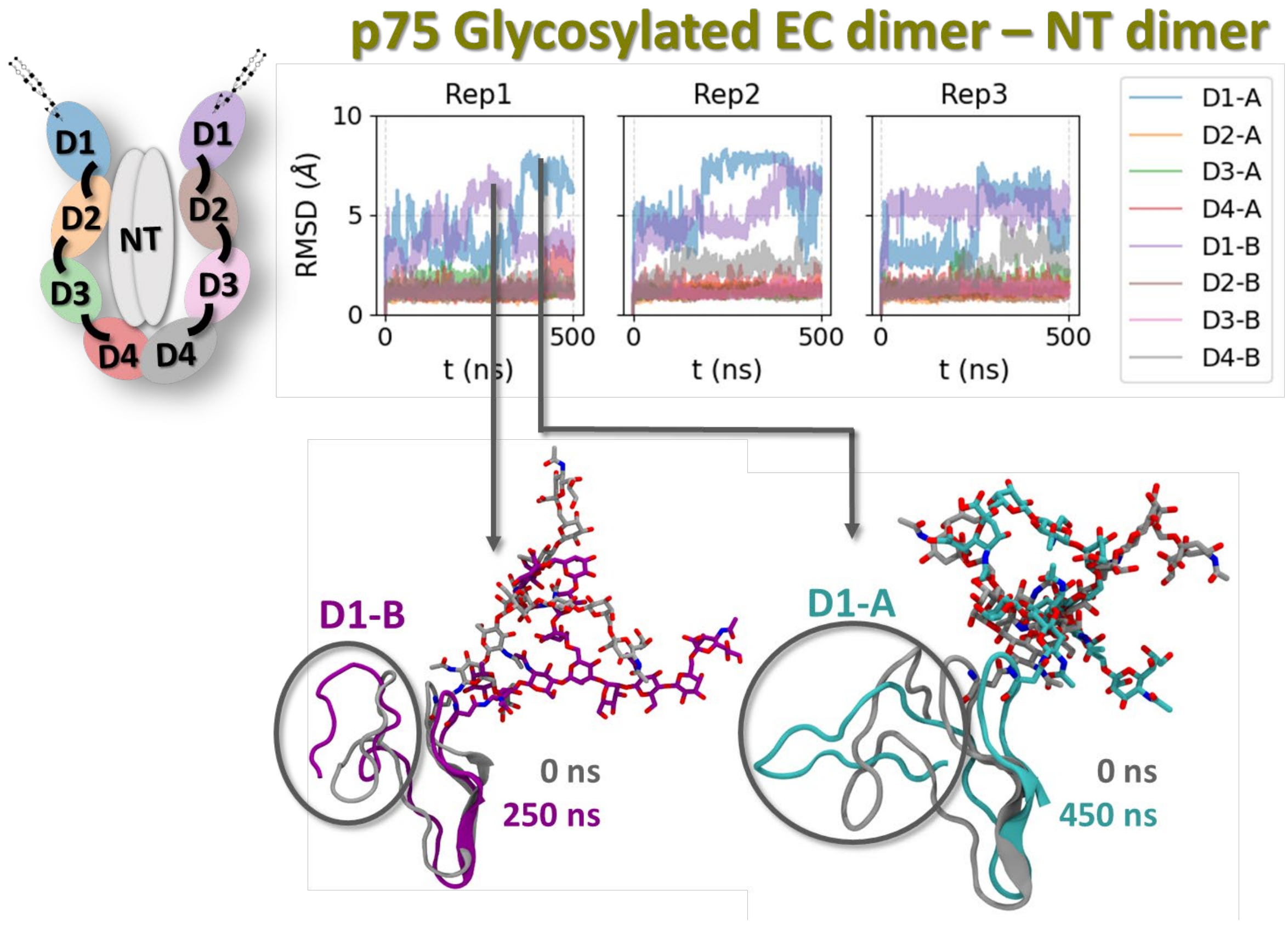

Figure S9: Closer examination of the p75-D1 domain conformations with the glycan at Asn 60, which is covalently attached to the D1 domain. Two example representations of high RMSD values are shown with D1-glycan aligned to the input structure from X-ray. The high RMSD conformations are shown in purple and cyan for the two D1 monomers and the initial structures in grey. The D1 domain is shown in cartoon representation, while the glycan is shown in stick representation.

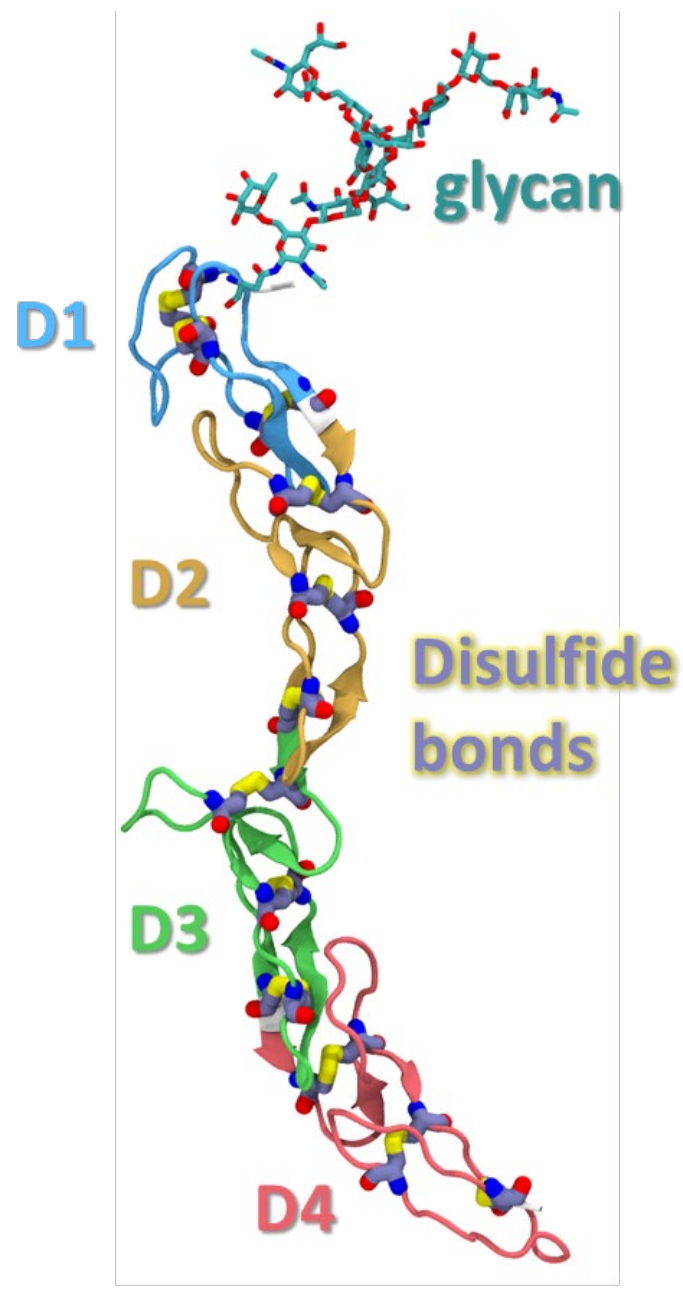

Figure S10: Disulfide bond ladder in the structure of p75-EC (from PDB ID: 3BUK). The D1-D4 domains are shown in cartoon representation. The disulfide bonds are shown in licorice representation.

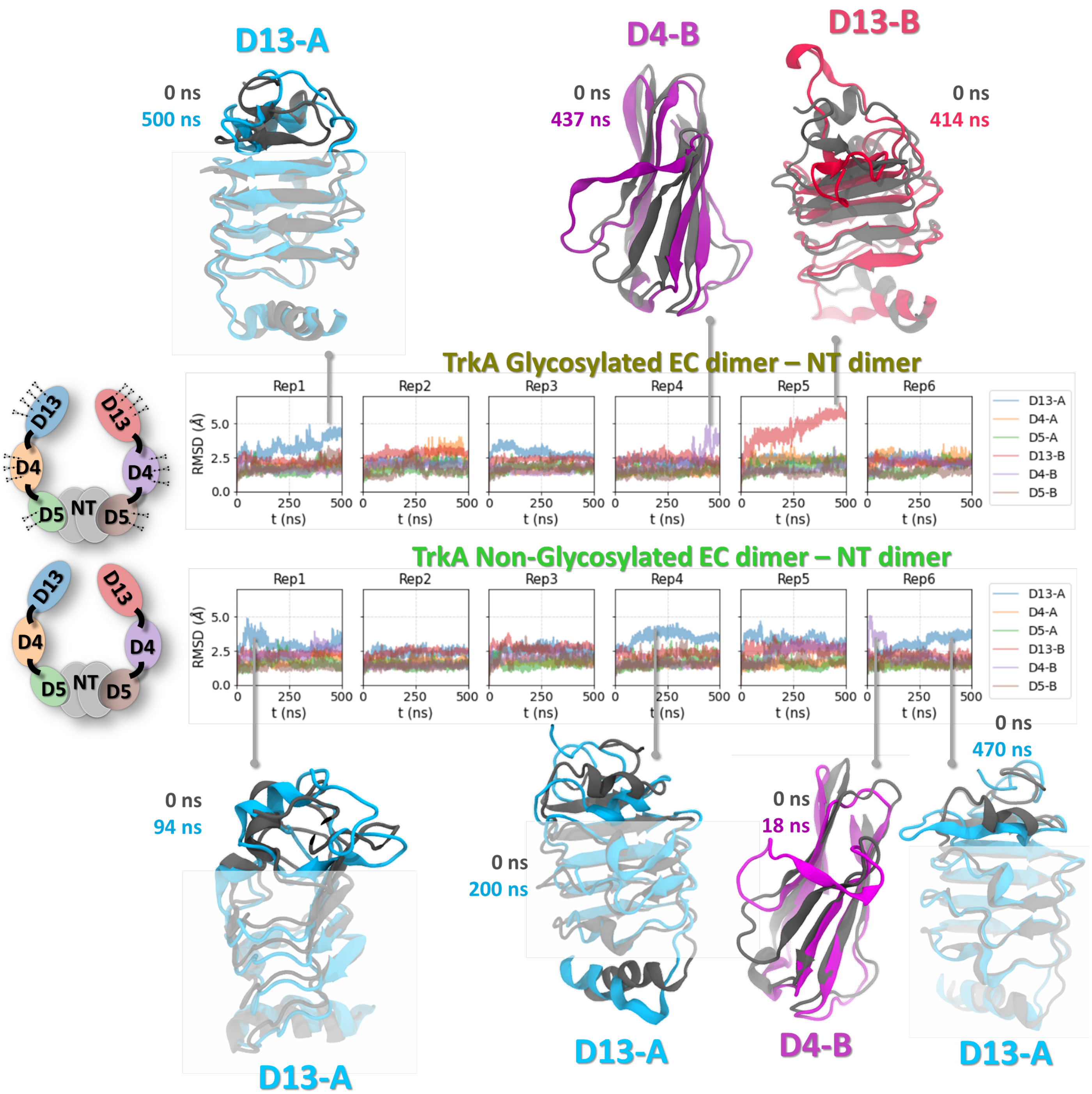

Figure S11: RMSD calculations for each domain of the glycosylated and not TrkA-EC homodimer bound to NGF system. For each system, the time-dependent RMSD of the domain backbone compared to the first frame is shown for each replica. Before the calculation of the internal RMSD of each domain, I performed alignment in the respective domain at each simulation snapshot. Schematic representation of each system, with color-coding of the domains is shown at the left. In cases of high RMSD values, the conformations of the domains are shown in cartoon representation aligned to the first frame.

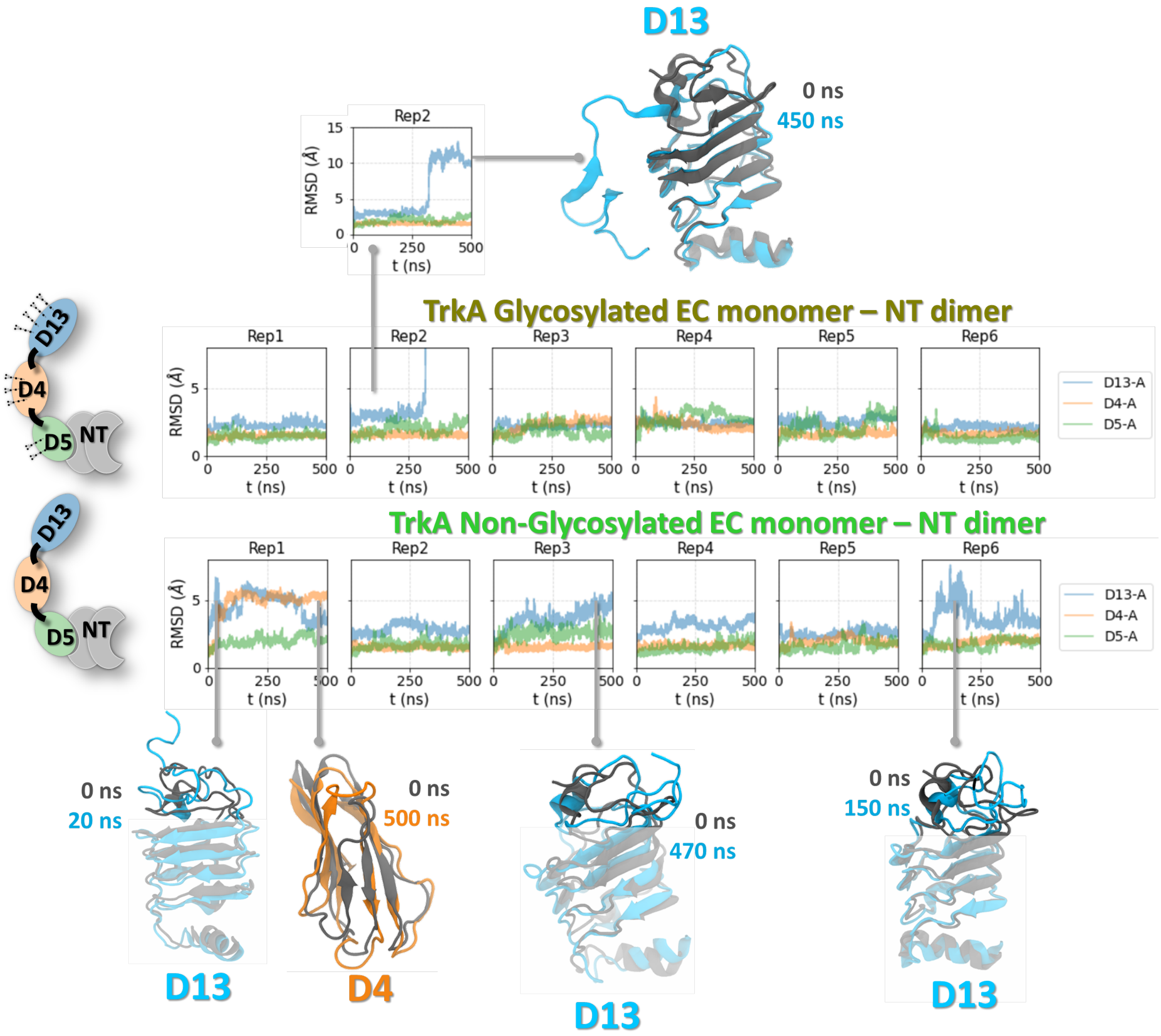

Figure S12: RMSD calculations for each domain of the glycosylated and not TrkA-EC monomer bound to NGF system. For each system, the time-dependent RMSD of the domain backbone compared to the first frame is shown for each replica. Before the calculation of the internal RMSD of each domain, I performed alignment in the respective domain at each simulation snapshot. Schematic representation of each system, with color-coding of the domains is shown at the left. In cases of high RMSD values, the conformations of the domains are shown in cartoon representation aligned to the first frame.

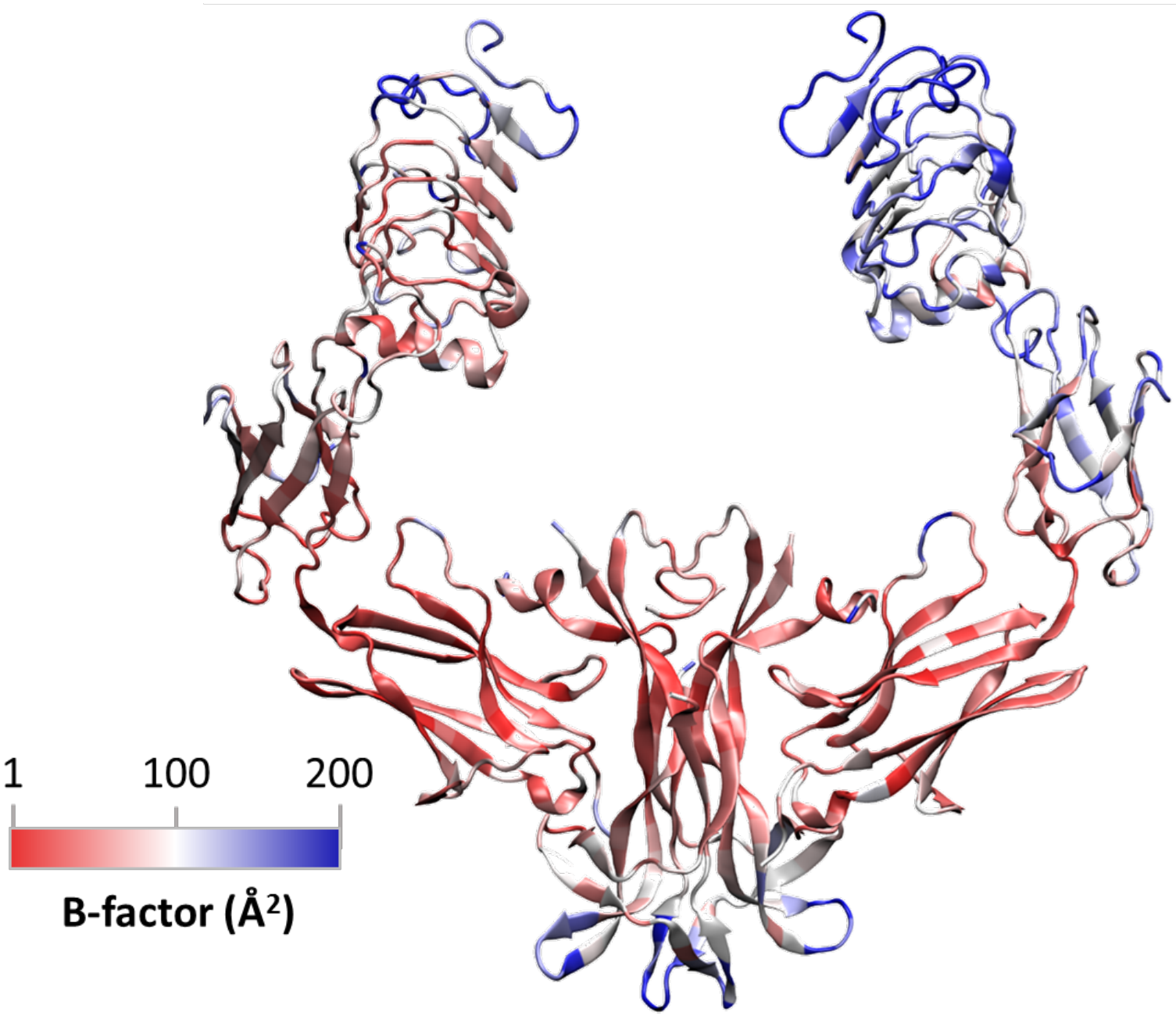

Figure S13: B-factors of the TrkA-EC bound to NGF from the crystal structure (PDB ID: 2IFG).[3]

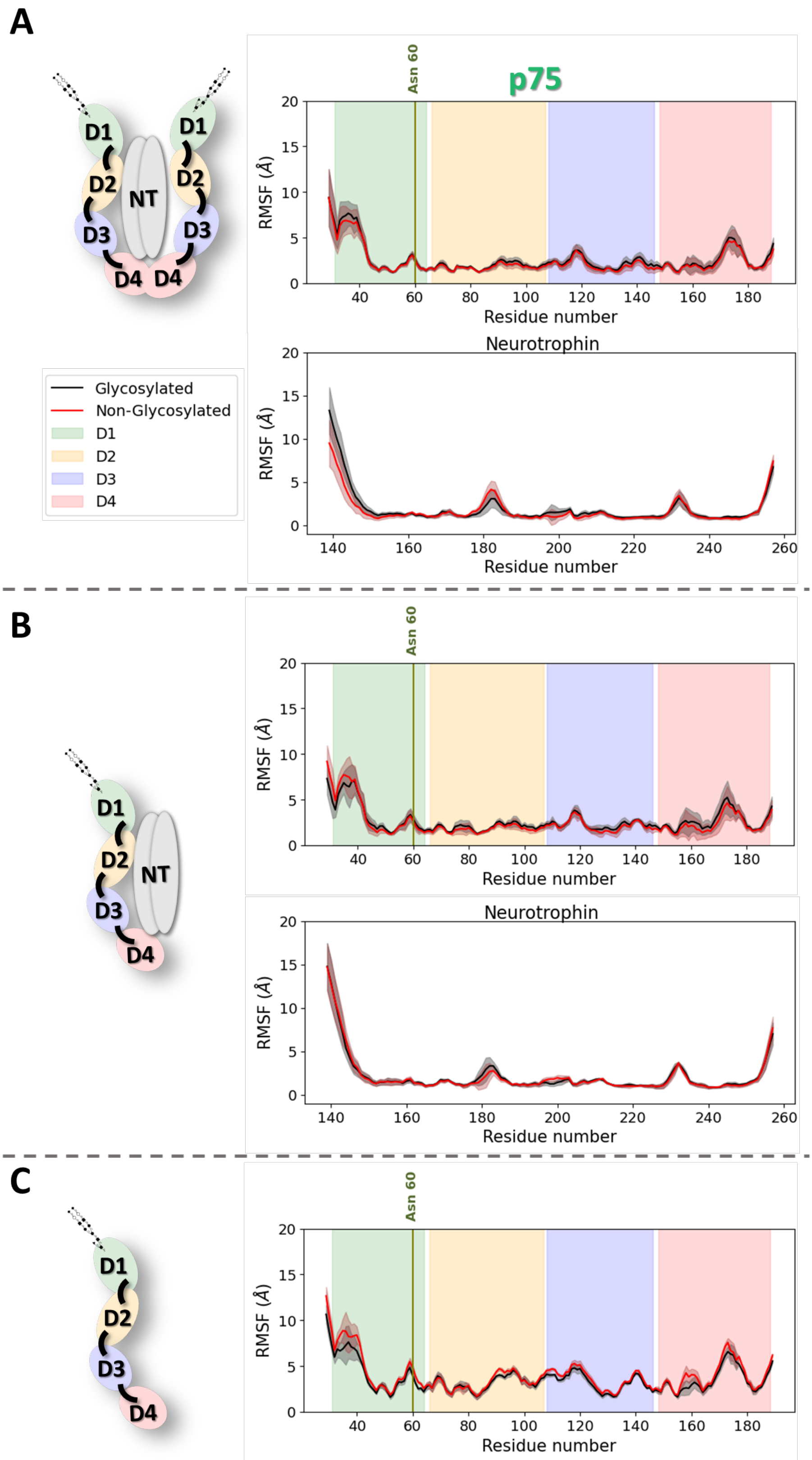

Figure S14: Summary of RMSF calculation results for the (A) p75-EC as a homodimer in complex to NT-3, (B) as a monomer in complex with NT-3 and (C) as a monomer alone, with (black) and without (red) glycans. The RMSF for each residue was calculated as an average from the three replica simulations and the standard deviation at each point is shown with shadowed areas. The different domains of p75-EC are color-coded and the glycosylated Asn 60 is indicated with a vertical line.

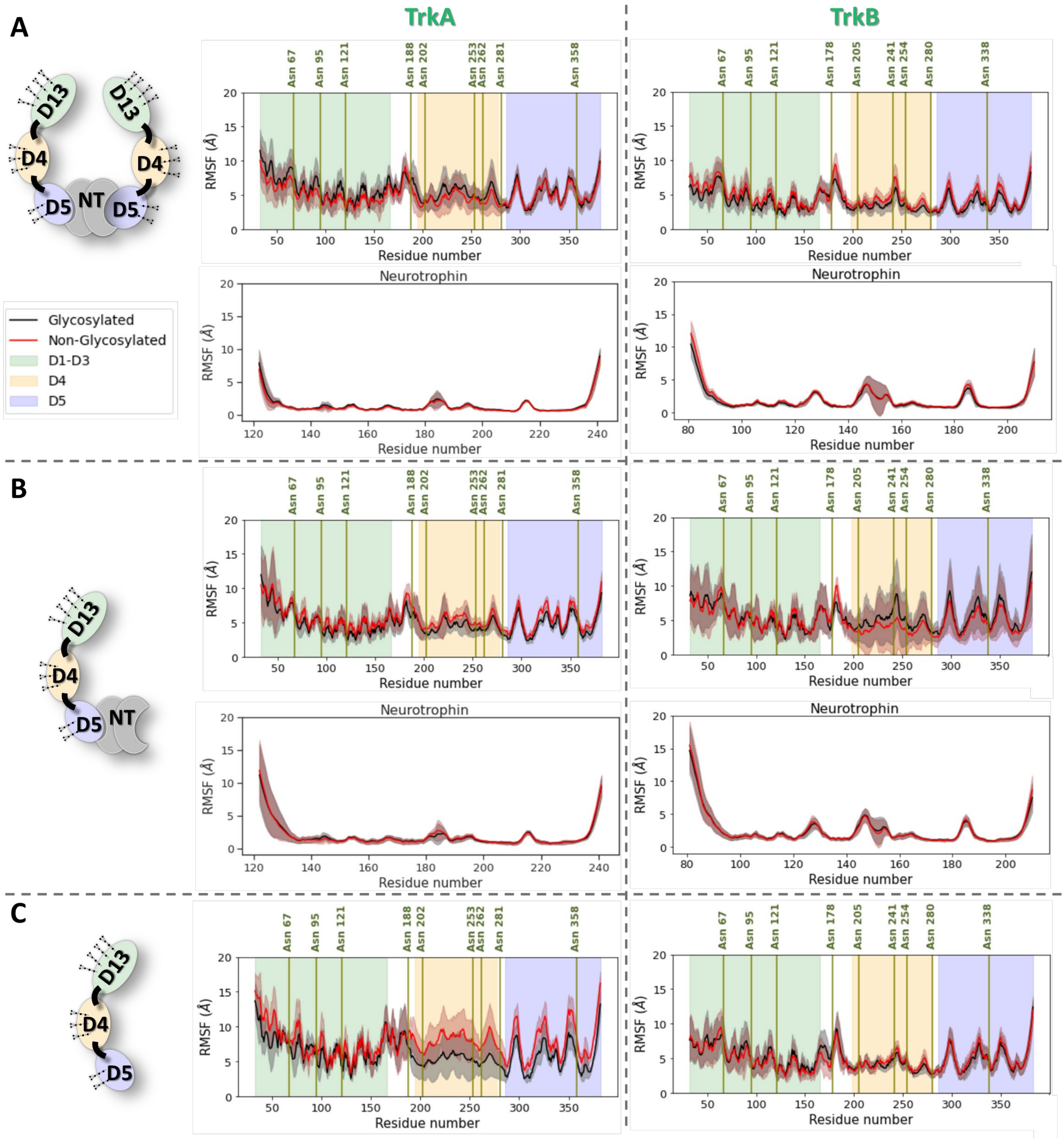

Figure S15: RMSF calculations of all the glycosylated or not TrkA and TrkB systems. (A), (B) and (C) show the average RMSF values from all replica simulations for each receptor and neurotrophin residue of the TrkA/B-EC homodimer with the NGF/ NT-4/5 homodimer bound, the TrkA/B-EC monomer with the NGF/ NT-4/5 homodimer bound, the TrkA/B-EC monomer systems, respectively, with or without glycans. The average RMSF values from all replica simulations are shown for each residue in black and red lines for the glycosylated and non-glycosylated systems, respectively. The standard deviation for each residue is shown with shadowed areas. The residue positions of glycosylated asparagines are shown with vertical lines. Before the RMSF calculation, I aligned the systems to an average structure from the simulations.

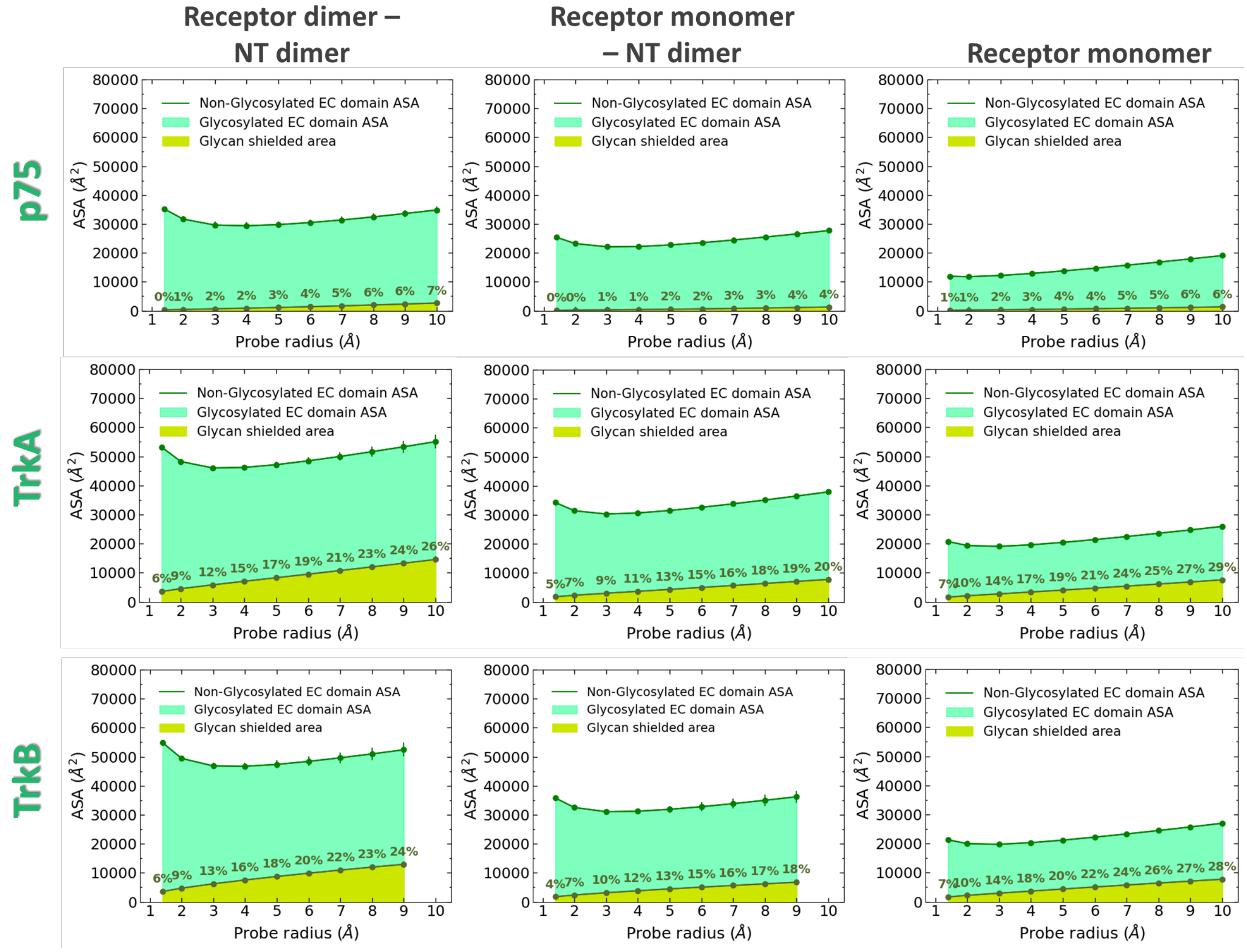

Figure S16: Accessible surface area (ASA) of the p75, TrkA and TrkB systems and the area shielded by glycans at multiple probe radii from 1.4 Å (water molecule) to 10 Å (protein-sized molecule). The values have been calculated and averaged across all replicas with glycans and are reported with standard deviation bars. The area shielded by the glycans is presented in yellow (rounded % values are reported), whereas the green line represents the accessible area of the protein in the absence of glycans. Highlighted in green is the area that remains accessible in the presence of glycans. The ASA for the TrkB dimer and monomer systems, which was calculated for probe radius 10 Å, is not shown because of incorrect calculation of the ASA in this high radius for the particular systems.

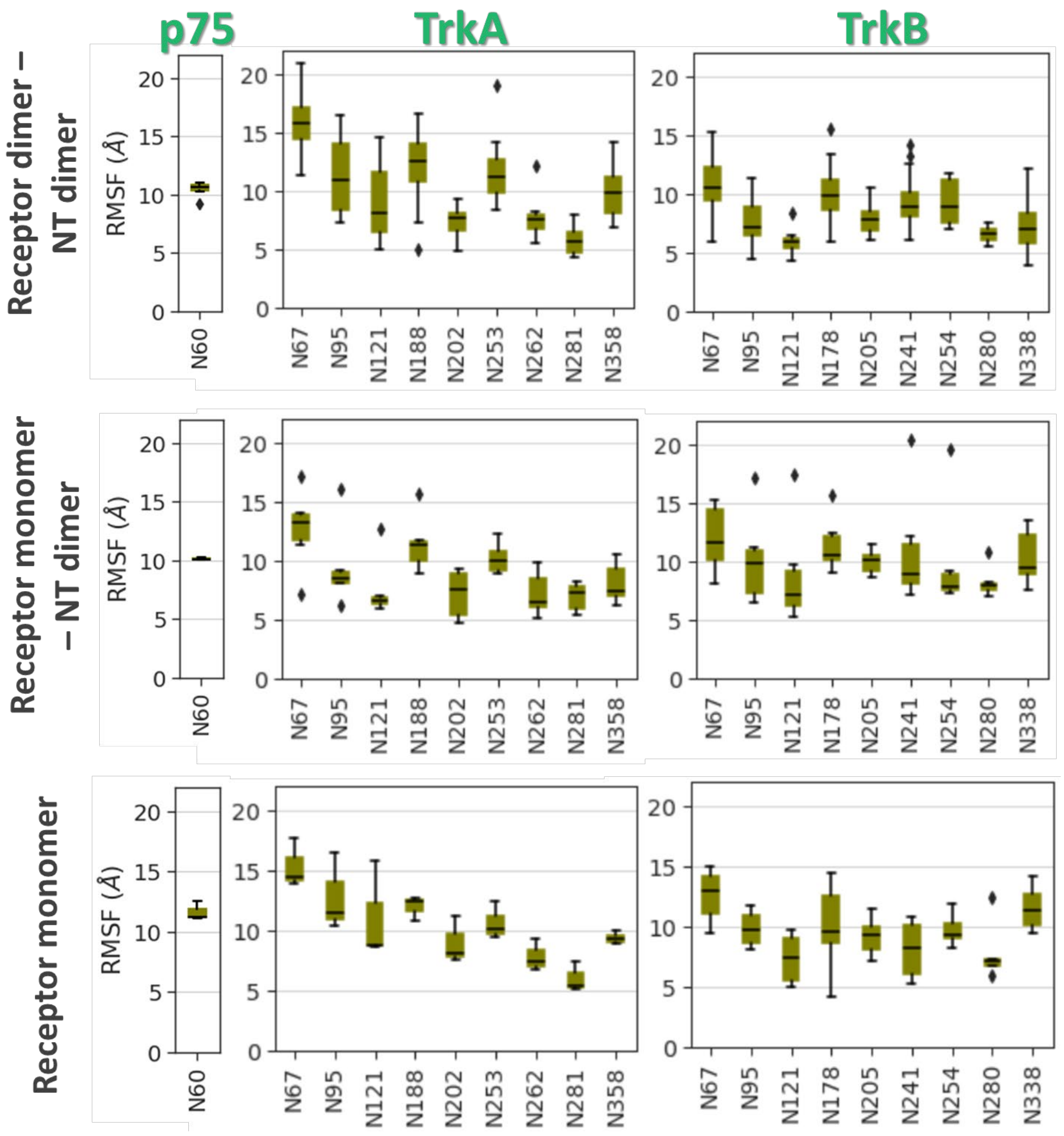

Figure S17: Box plots of RMSF calculations of all glycans attached in asparagine residues in all p75, TrkA and TrkB glycosylated systems. Before the RMSF calculation, alignment of each EC segment was performed to its initial structure (frame 0).

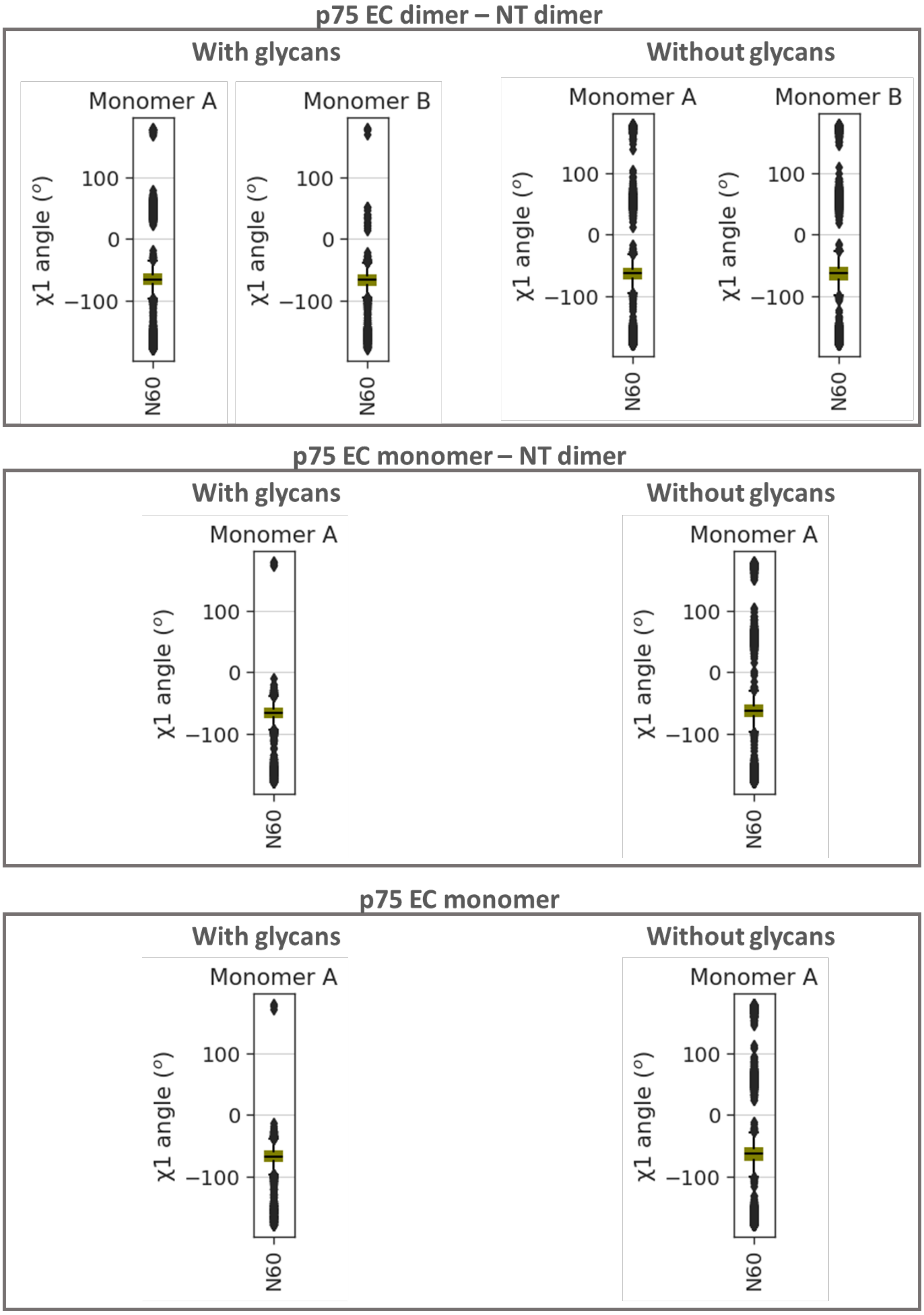

Figure S18: Distributions of the χ1 dihedral angle of N60 for the three p75-EC systems with and without the glycans present.

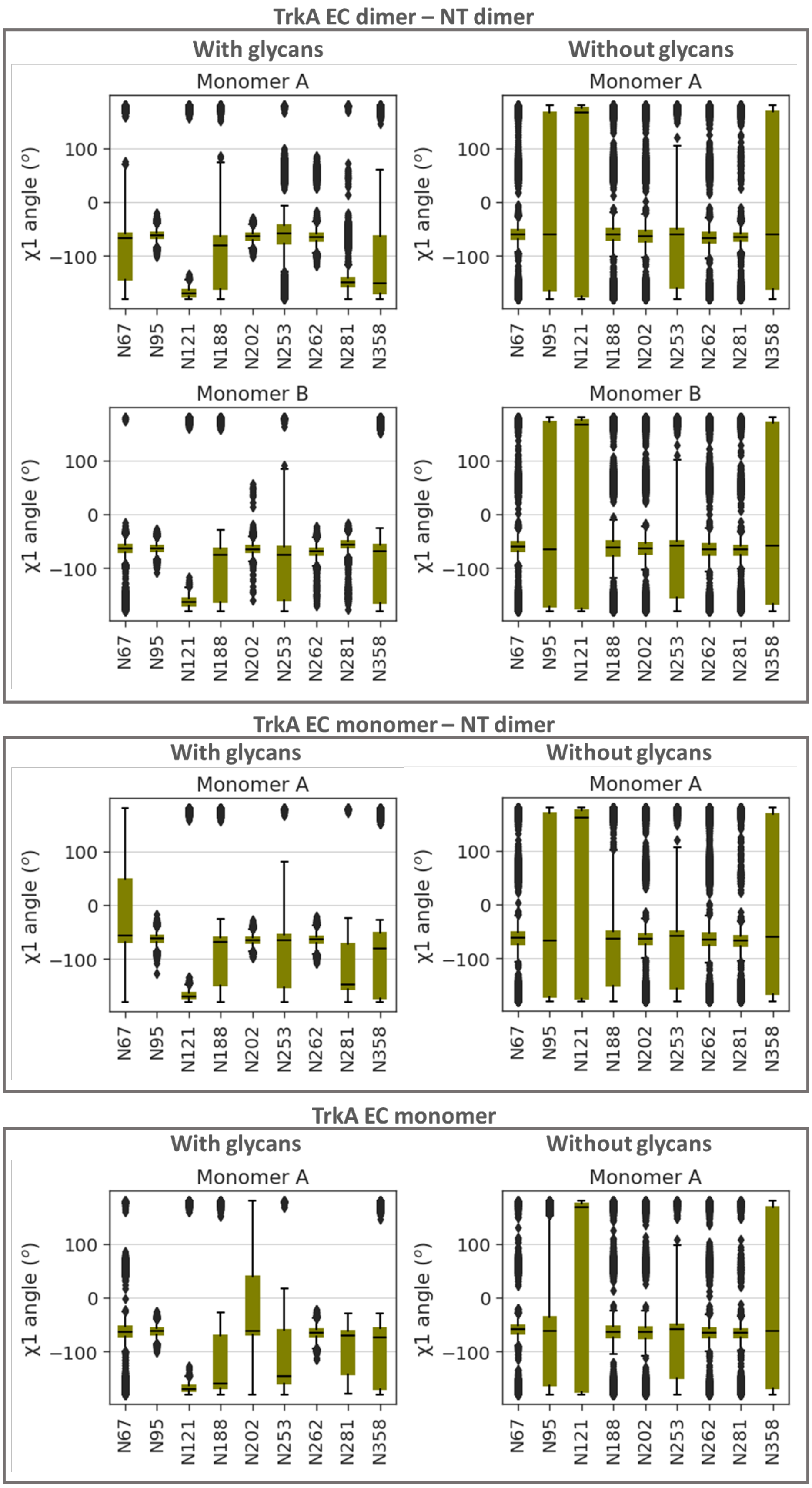

Figure S19: Box plots of the distribution of χ1 dihedral angle of glycosylated Asn residues. A comparison of the χ1 distributions is shown between glycosylated and non-glycosylated TrkA systems.

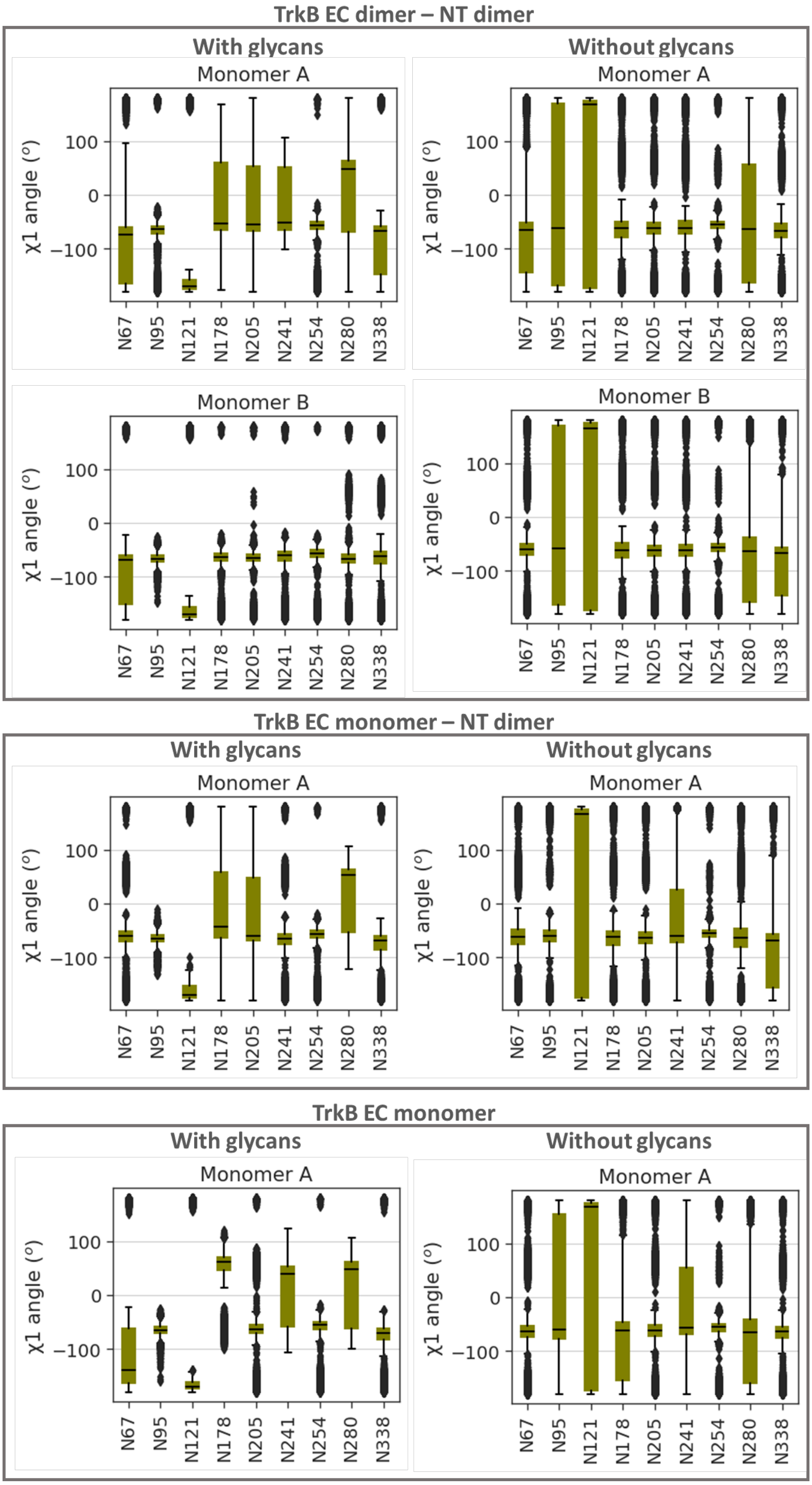

Figure S20: Box plots of the distribution of χ1 dihedral angle of glycosylated Asn residues. A comparison of the χ1 distributions is shown between glycosylated and non-glycosylated TrkB systems.

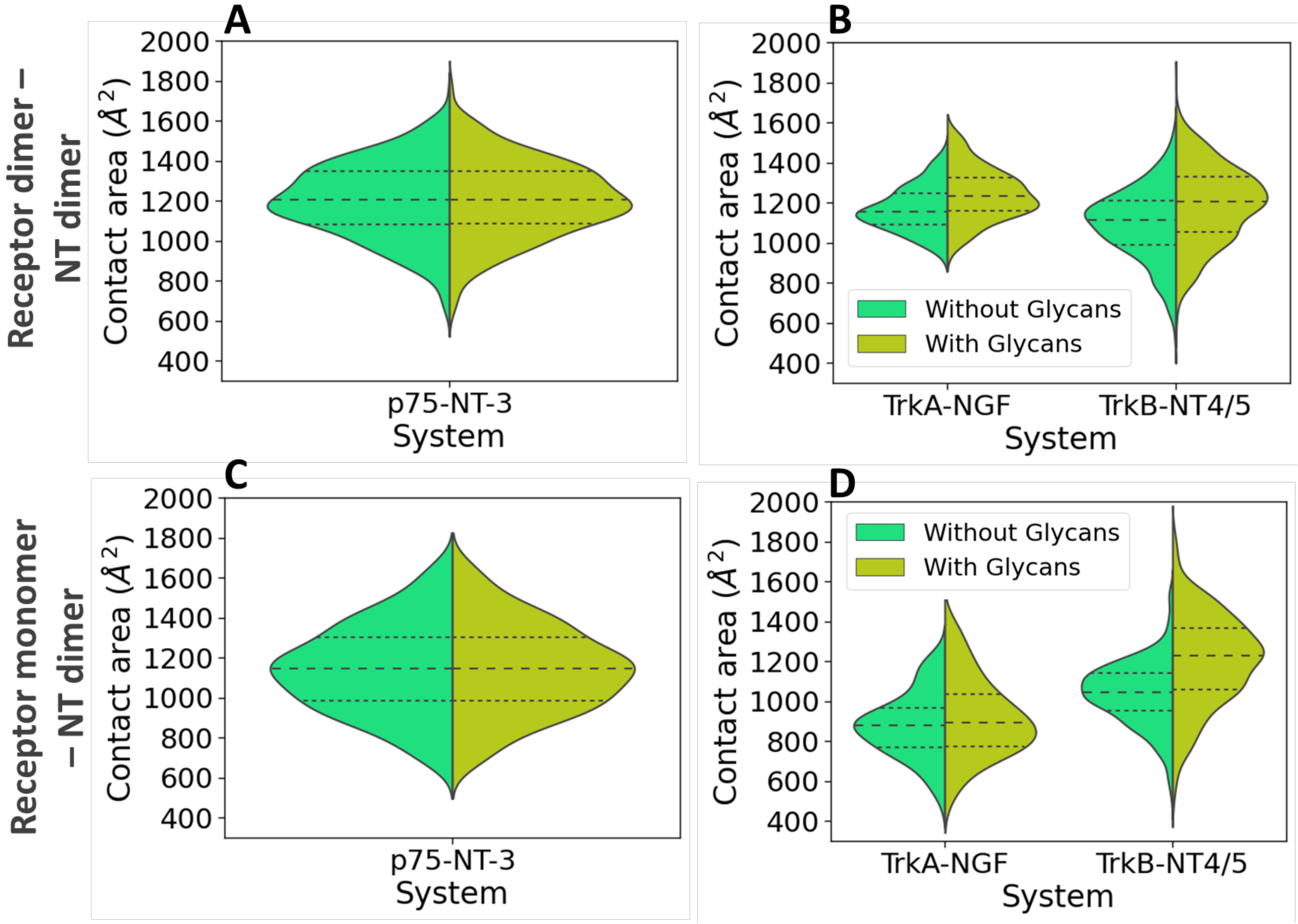

Figure S21: Violin plots with the contact area between the EC domains of p75, TrkA and TrkB receptors with the NTs. The systems with the EC receptor dimer bound to NTs with and without glycans are shown in (A) and (B), while the systems with the EC receptor monomers bound to NTs with and without glycans are shown in (C) and (D). For the systems with the EC dimers, the contact area for each monomer was calculated separately.

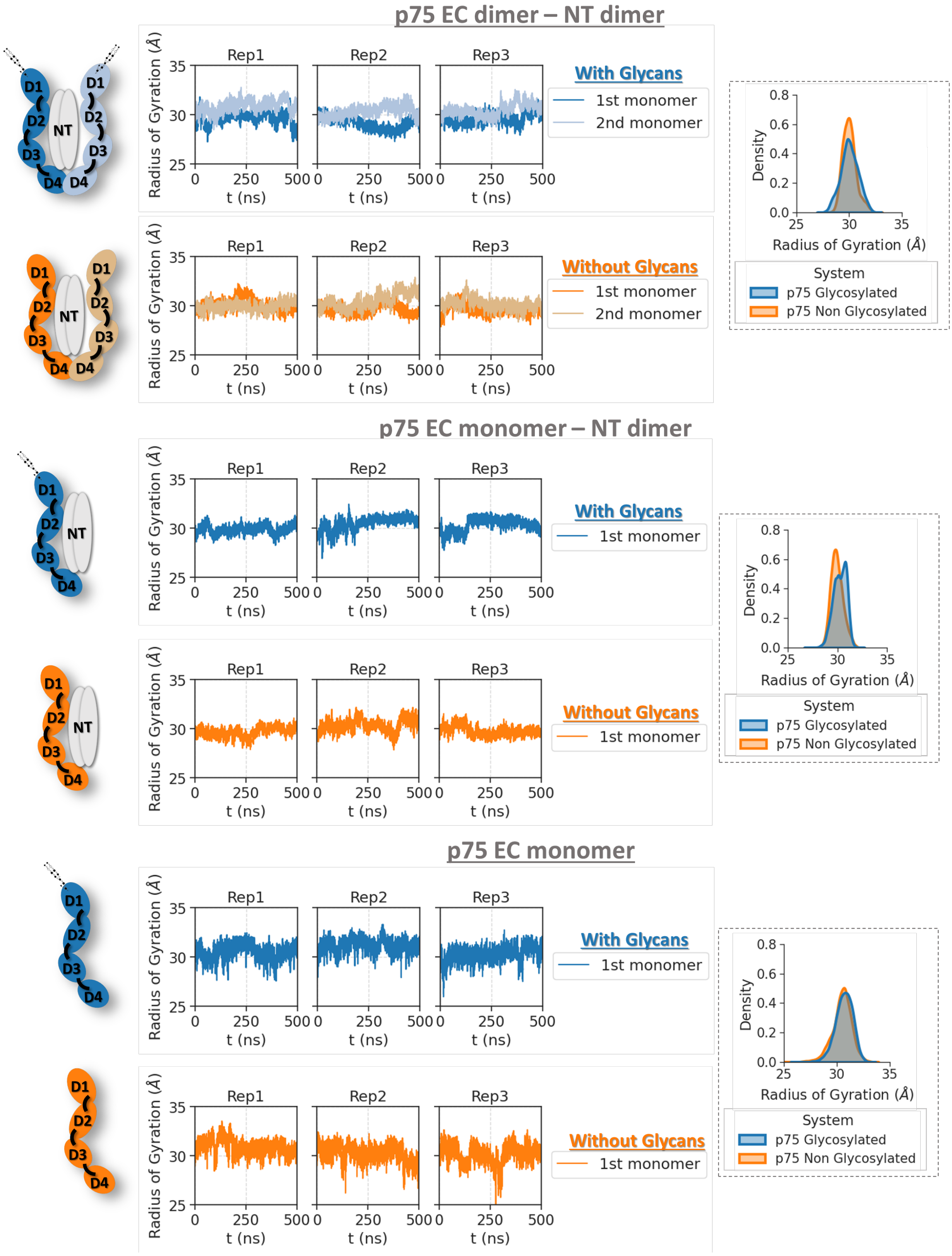

Figure S22: Time evolution and distribution of the radius of gyration values for the systems of p75-EC as a homodimer in complex to NT-3, as a monomer in complex with NT-3 and as a monomer alone, with (blue) and without (orange) glycans. For the system of p75-EC as a homodimer in complex to NT-3, the radius of gyration was calculated for both p75-EC domains separately.

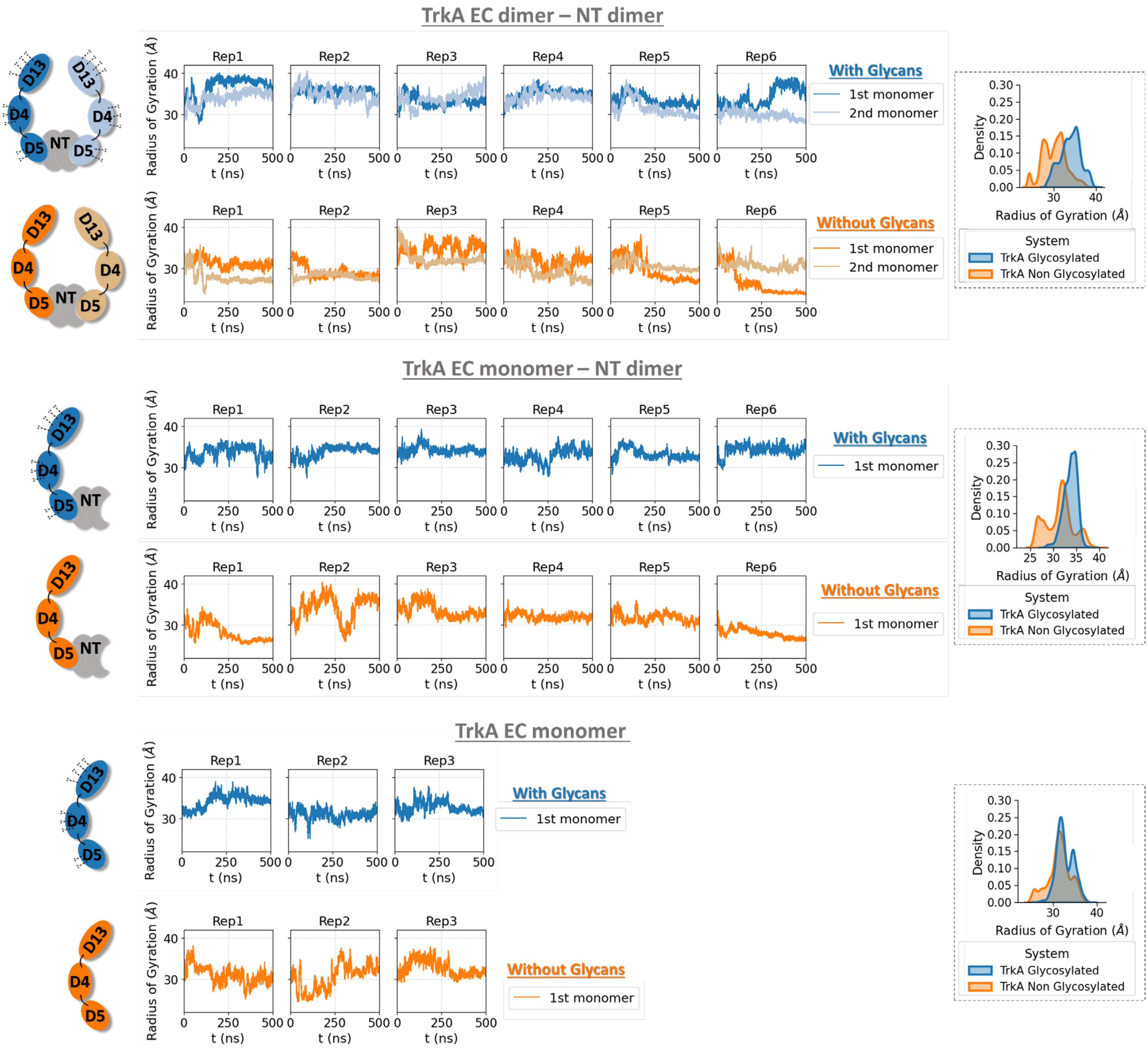

Figure S23: Radius of gyration of the receptor backbone of the glycosylated and not TrkA systems. The color-coding of each domain is shown in the schematic representations of the systems at the left of the plots. The time-evolution of the radius of each receptor monomer is shown for each replica, as well as the distributions of the radius of gyration values across all replicas for the glycosylated and non-glycosylated systems (right plots).

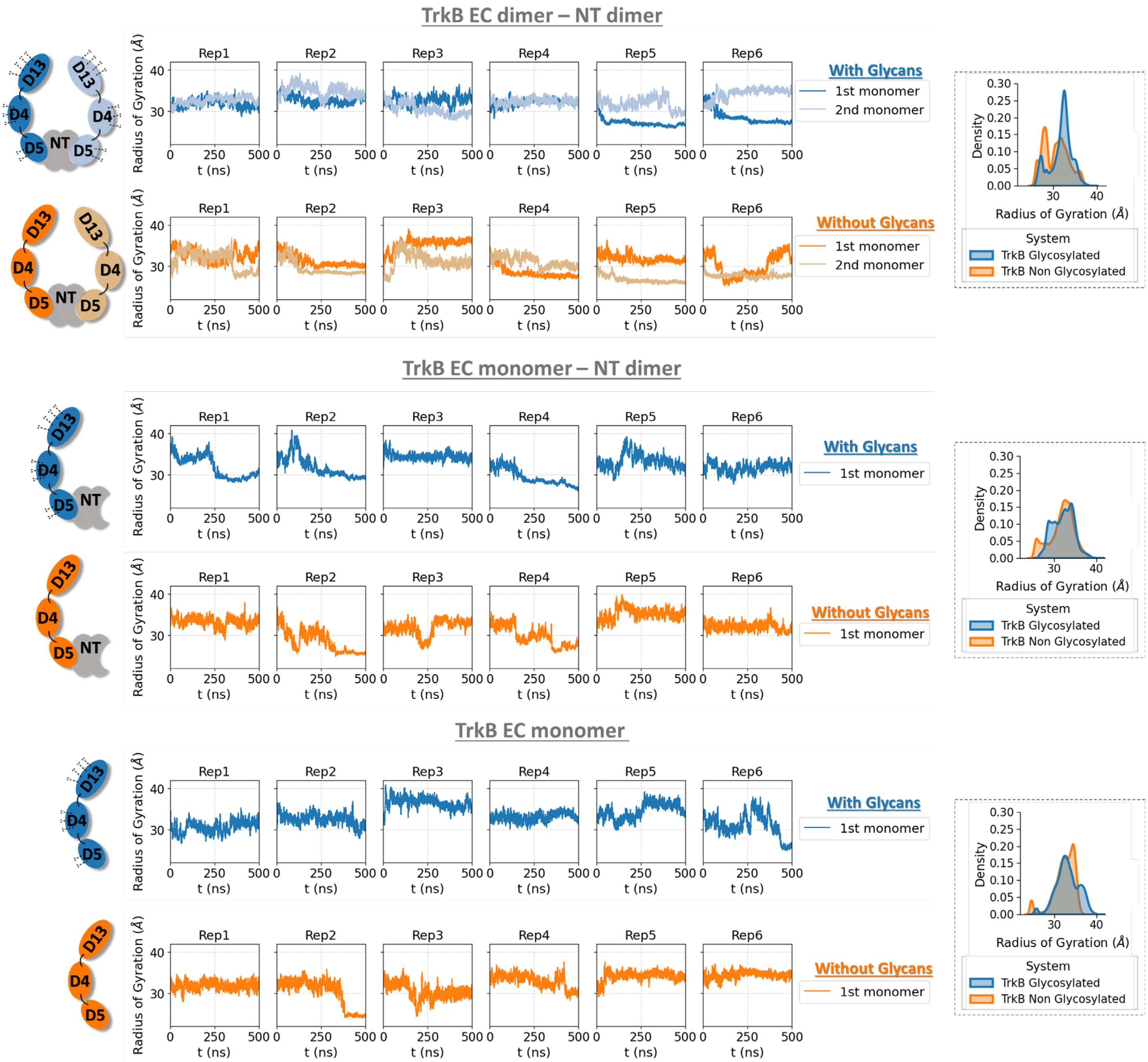

Figure S24: Radius of gyration of the receptor backbone of the glycosylated and not TrkB systems. The color-coding of each domain is shown in the schematic representations of the systems at the left of the plots. The time-evolution of the radius of each receptor monomer is shown for each replica, as well as the distributions of the radius of gyration values across all replicas for the glycosylated and non-glycosylated systems (right plots).

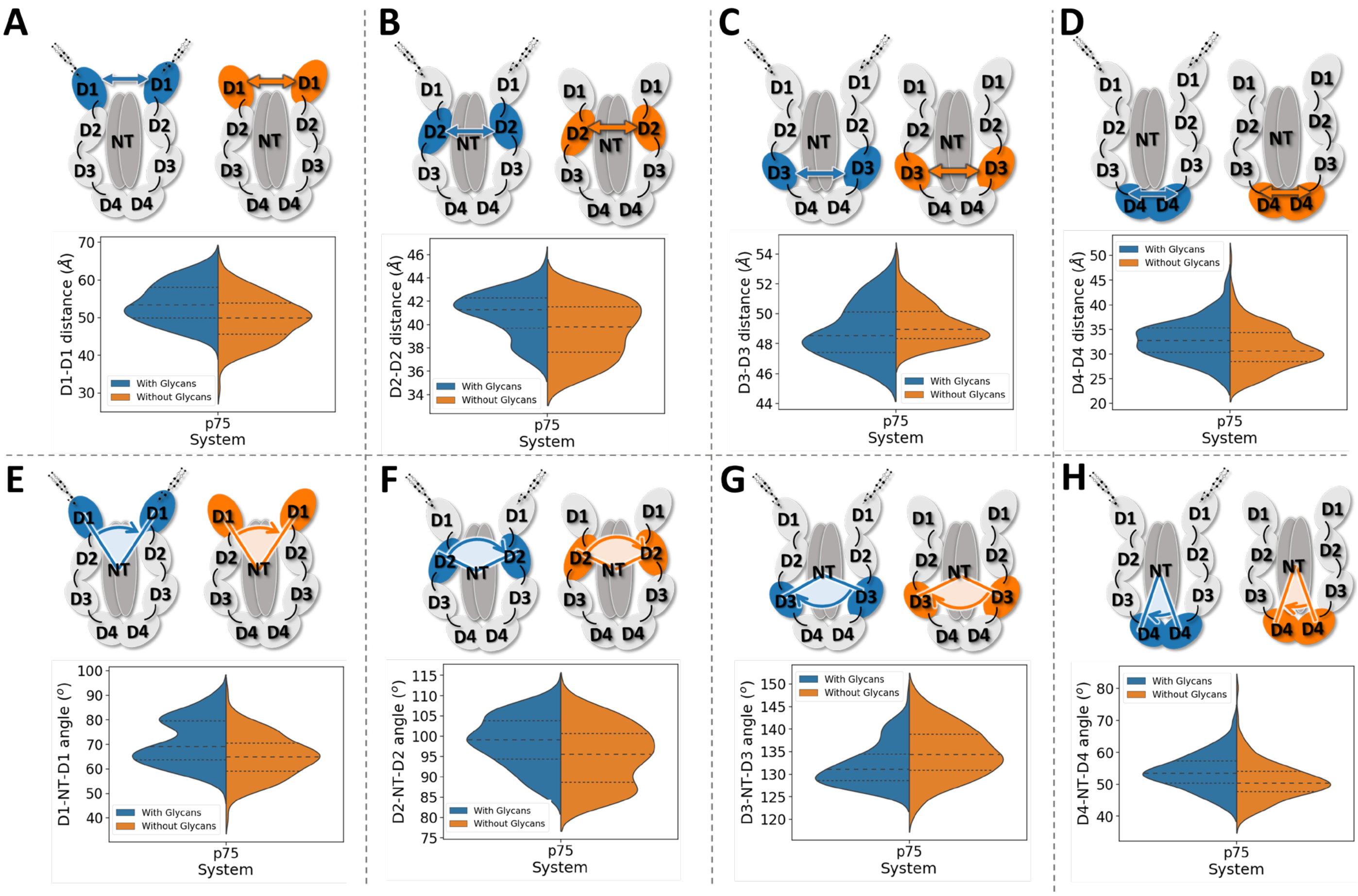

Figure S25: Calculated distributions of the distances and angles between the four p75-EC domains. (A)-(C) Distances between the COGs of D1-D1, D2-D2, D3-D3 and D4-D4 domains, in the presence (blue) and absence (orange) of glycans. (E)-(H) Angles defined by the COGs of D1-NT-D1, D2-NT-D2, D3-NT-D3 and D4-NT-D4 domains.

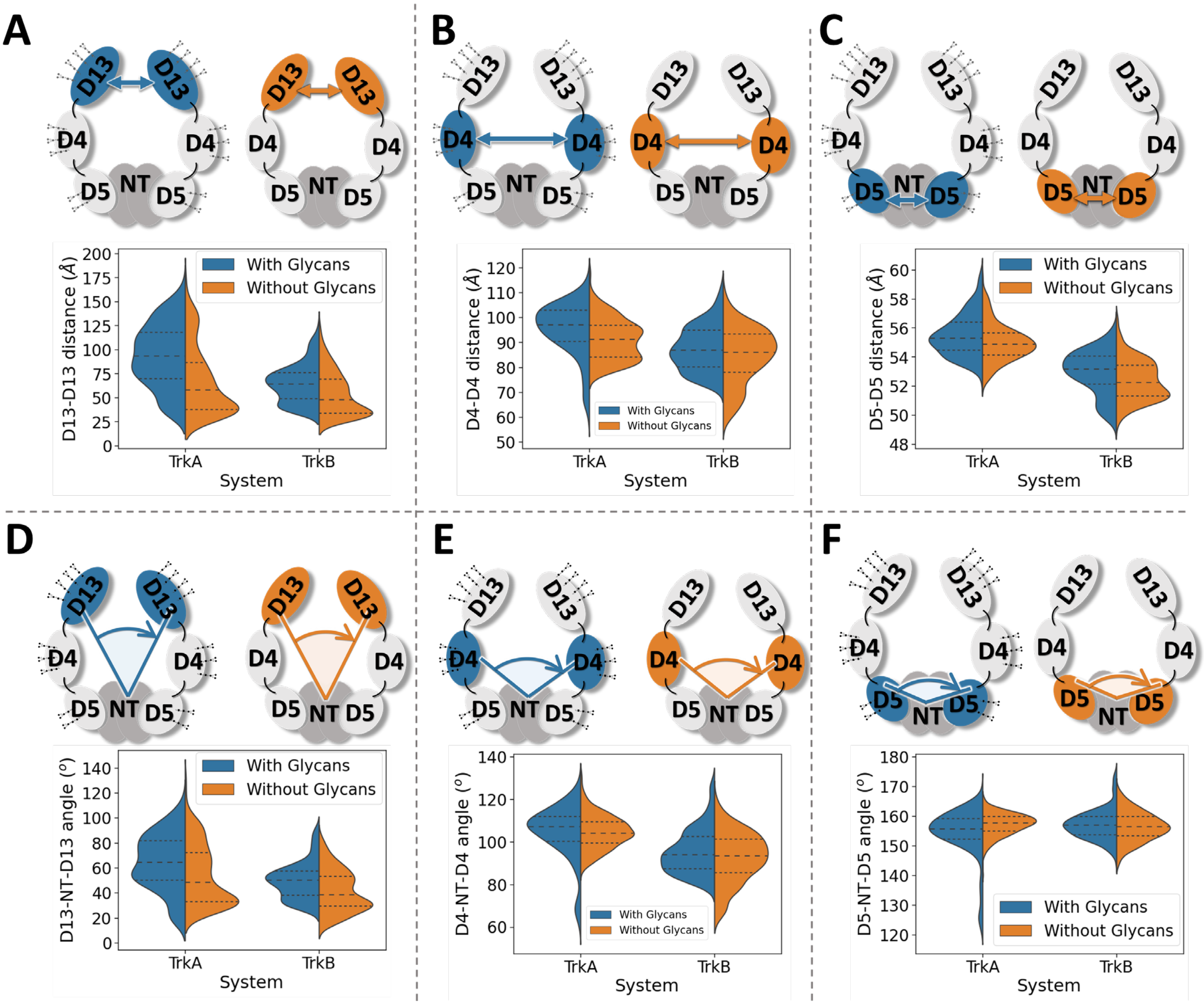

Figure S26: Distance and angle analysis between the receptor monomer domains. (A, B, C) Calculated distances between the centers of geometry (COG) of the D13, D4 and D5 domains from the two different receptor monomers. (D, E, F) Calculated angles formed between the COG of one of the same domains from the first receptor monomer, the COG of the NT and the COG of the same domain from the second monomer. In all cases, the distances and angles were calculated every 100 frames and the final distributions from the glycosylated and non-glycosylated TrkA and TrkB systems are shown in violin plots. The quartiles of the distributions are indicated with dashed lines.

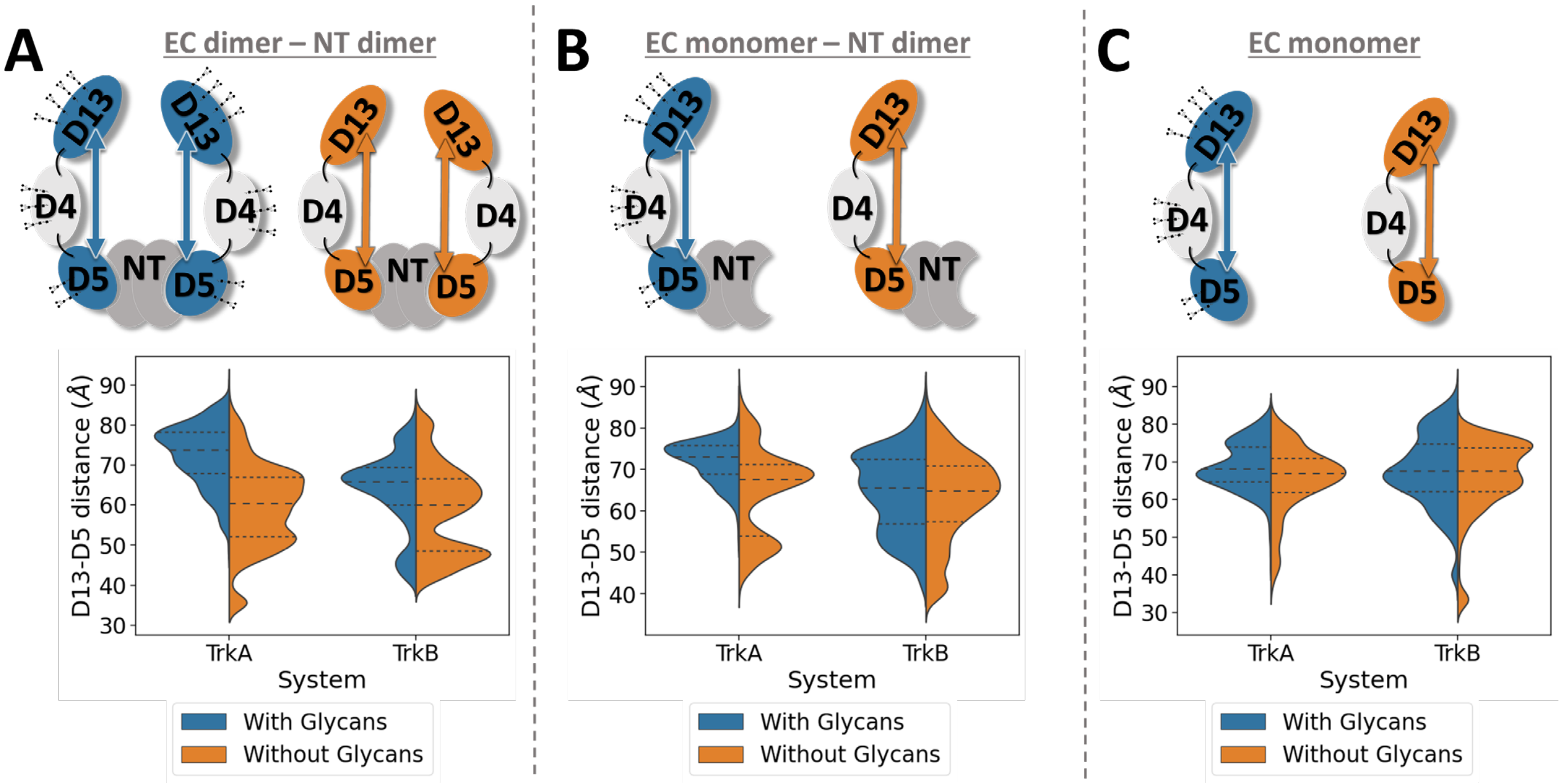

Figure S27: Distance between the centers of geometry of the D13-D5 domains in the different TrkA and TrkB systems (A, B, C). In all cases, the distances were calculated every 100 frames and the final distributions from the glycosylated and non-glycosylated TrkA and TrkB systems are shown in violin plots. The quartiles of the distributions are indicated with dashed lines.

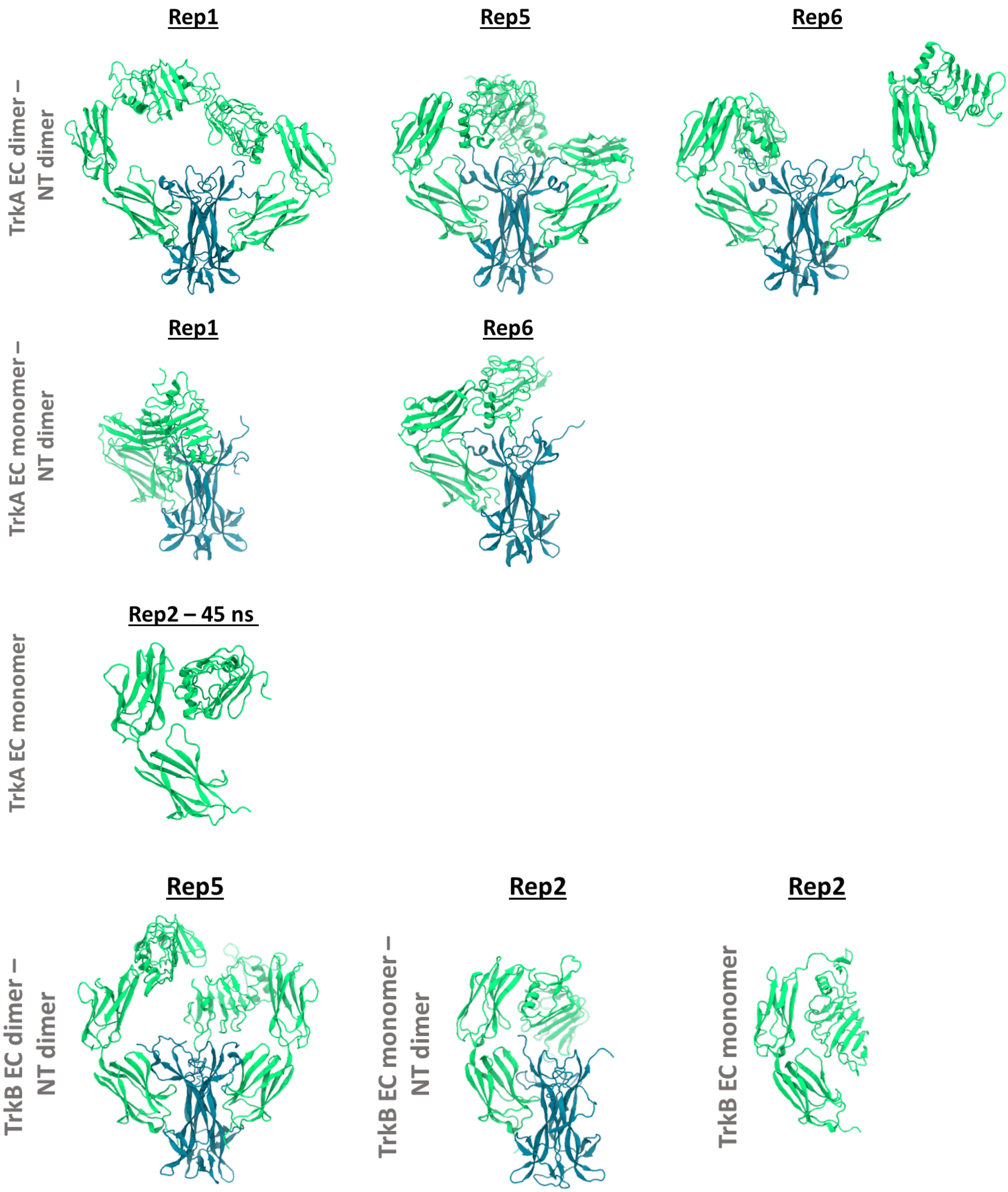

Figure S28: Conformations of the different non-glycosylated TrkA and TrkB systems, which correspond to low radius of gyration and inter-domain distance values. All the conformations are taken from the last frame of the indicated replica simulations, except for the last TrkA which is a rendering at 45 ns of the simulation.

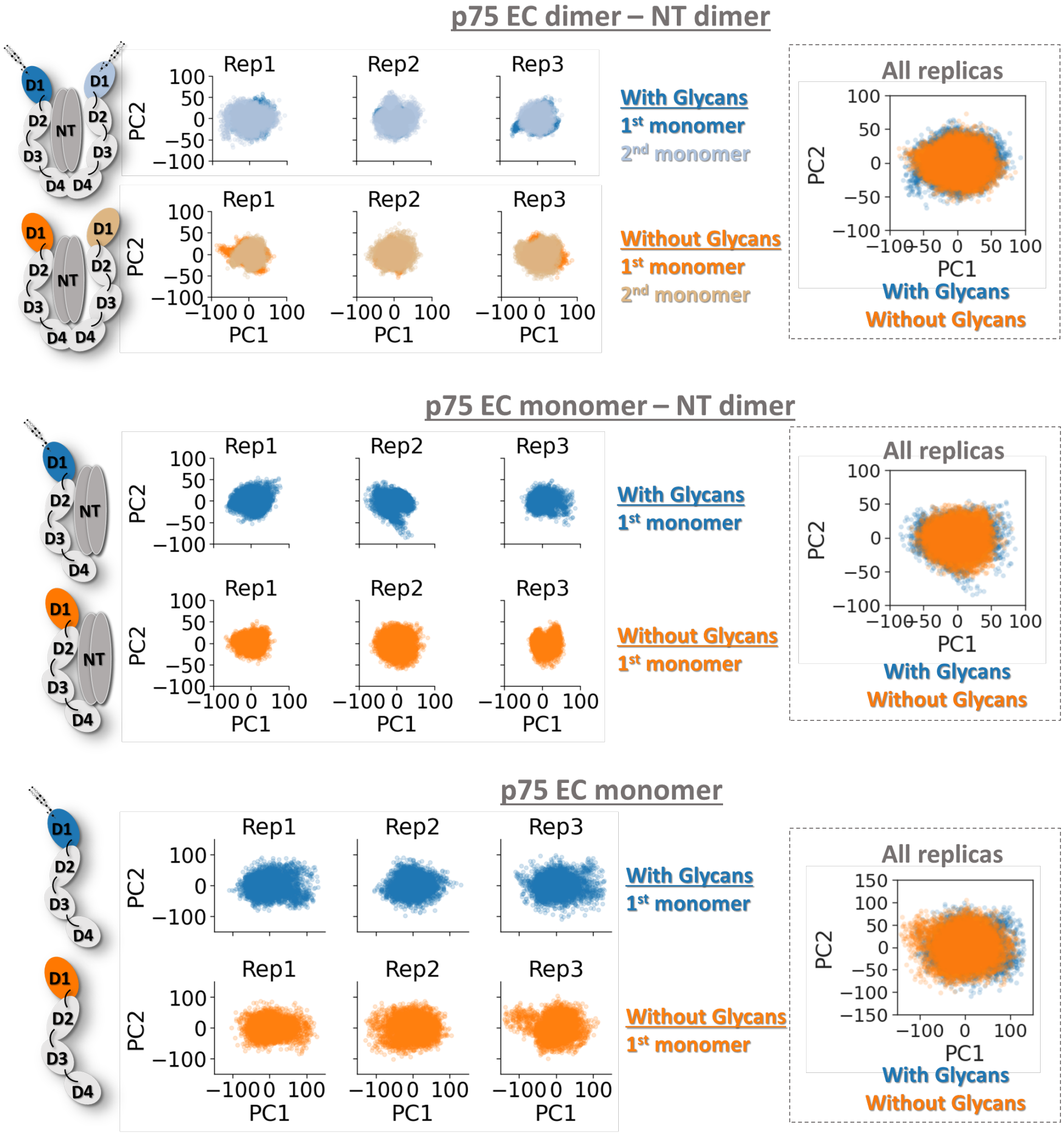

Figure S29: Principal component analysis (PCA) of the D1 domain of p75-EC after alignment to the rest of the domains for the glycosylated (blue) or not (orange) systems. Only the 1st and 2nd principal components are visualized.

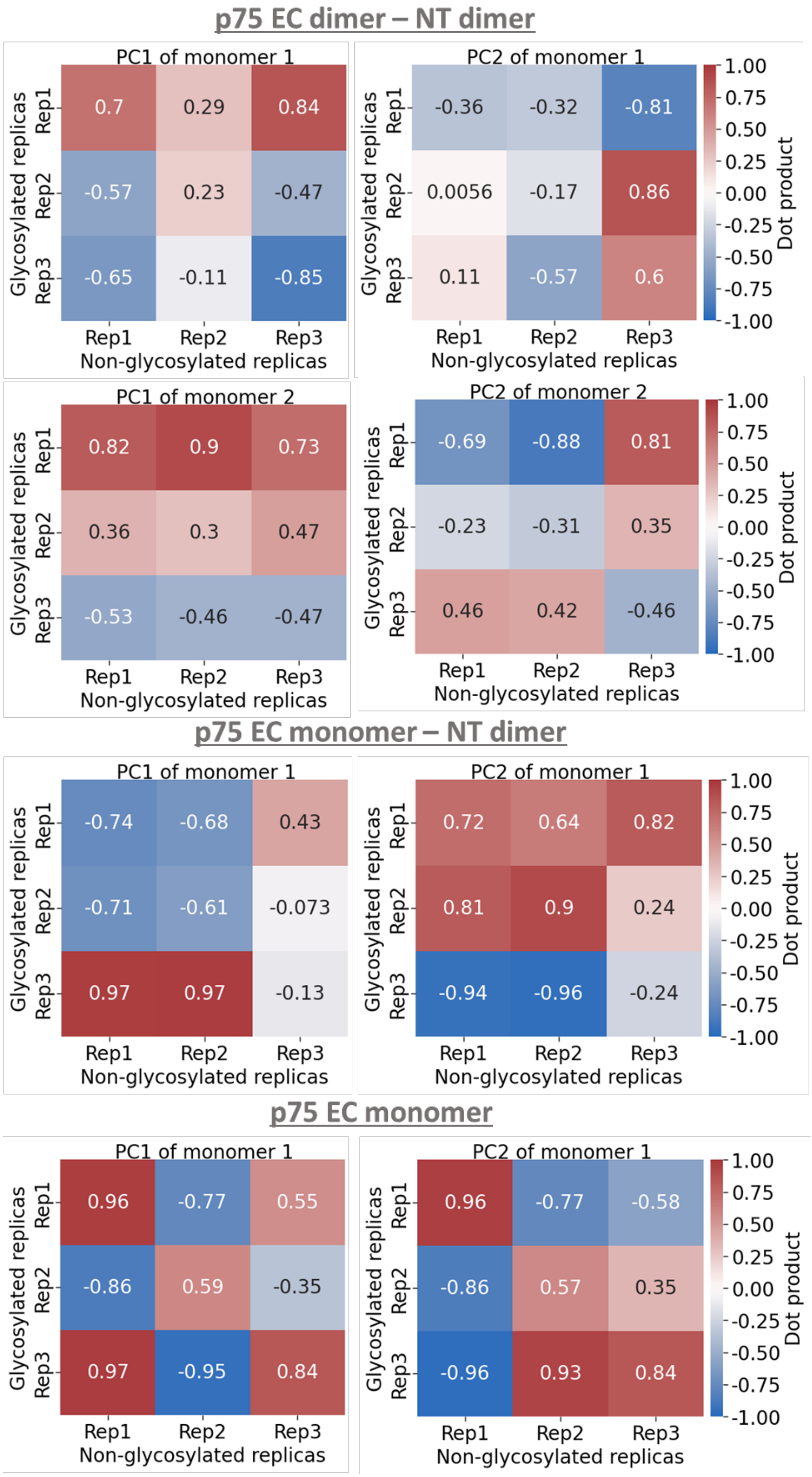

Figure S30: Dot products of the 1st and 2nd principal components from the various replicas and systems with and without glycans. The dot product values are also color-coded with heatmaps, with values equal to 1 showing parallel vectors, values of -1 showing antiparallel vector and 0 meaning vertical vectors. PCA analysis was performed separately for each replica simulation. As a result, the PC1 and PC2 are not necessarily the same in all replica simulations. Thus, to compare the PCs between different replicas, the dot products between the PC1s and PC2s from the various replicas were calculated. The results showed that in some cases the PCs were pointing towards the same direction (positive dot products) between the glycosylated and non-glycosylated systems, while in other cases they pointed in opposite directions (negative dot products), or were even perpendicular to each other (close to zero dot products).

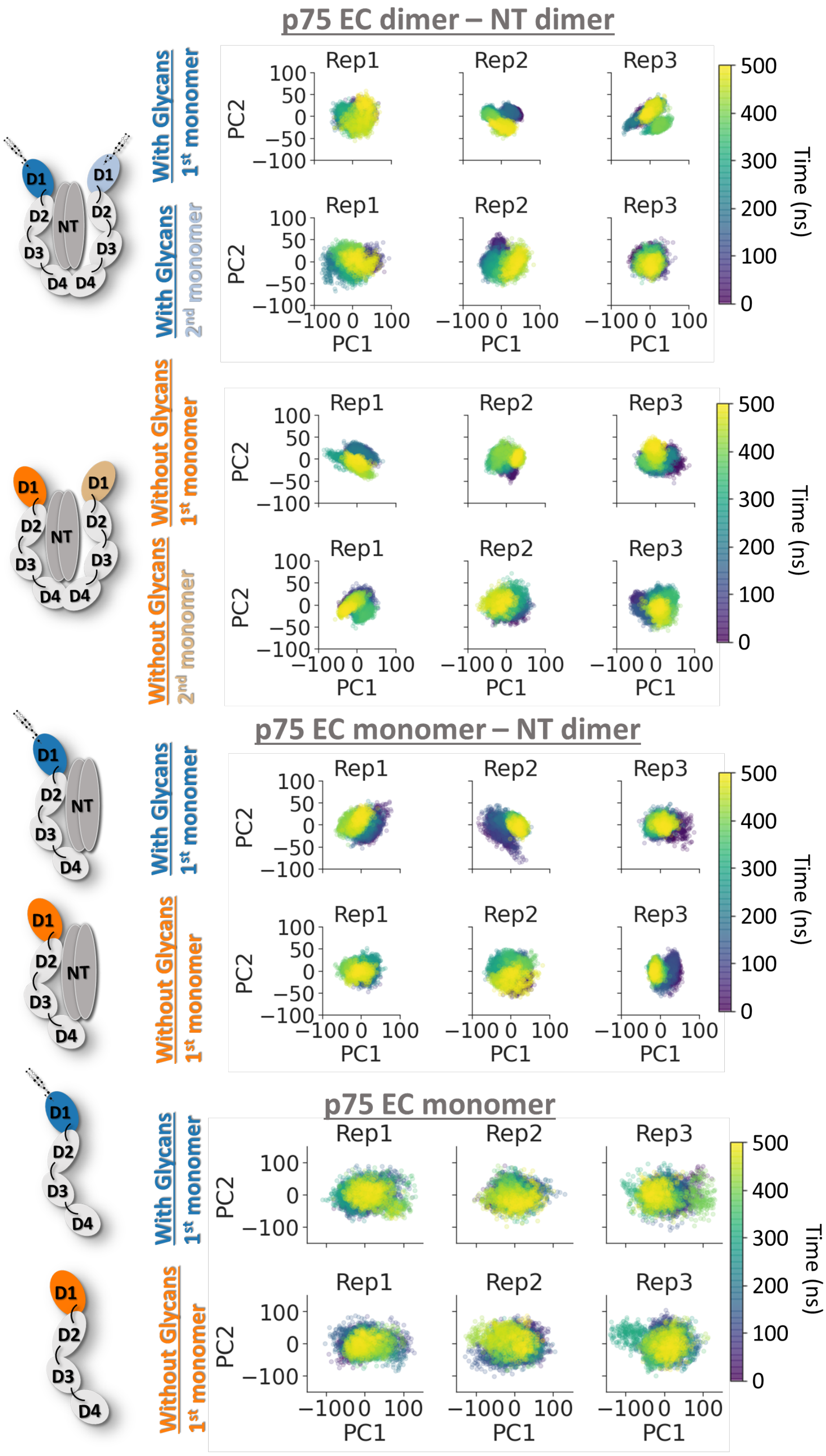

Figure S31: Principal component analysis (PCA) of the D1 domain of p75-EC as a time evolution after alignment to the rest of the domains, for the glycosylated or not systems. Only the 1st and 2nd principal components are visualized. The systems visit the same PC1-PC2 space at all times of the simulation, which is probably facilitated by the narrow space that is explored by the system, consistent with the previously discussed rigidity of the p75-EC domain.

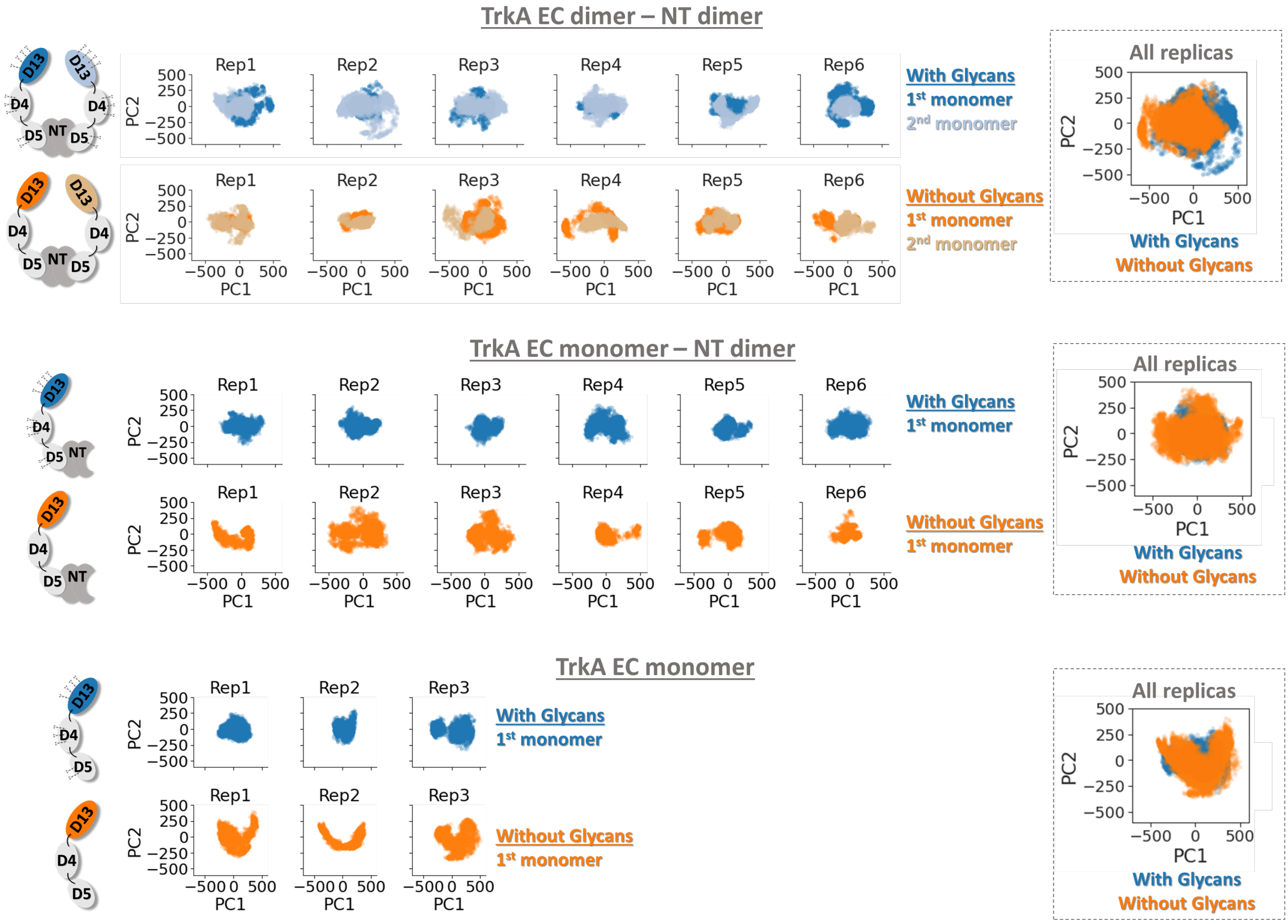

Figure S32: First and second principal components of the D13 domain during the simulations of the glycosylated and not TrkA systems. PCA analysis was performed to the D13 domains after alignment of each EC segment to the D4 and D5 domains. The color coding of each domain is shown in the schematic representations of the systems at the left of the plots.

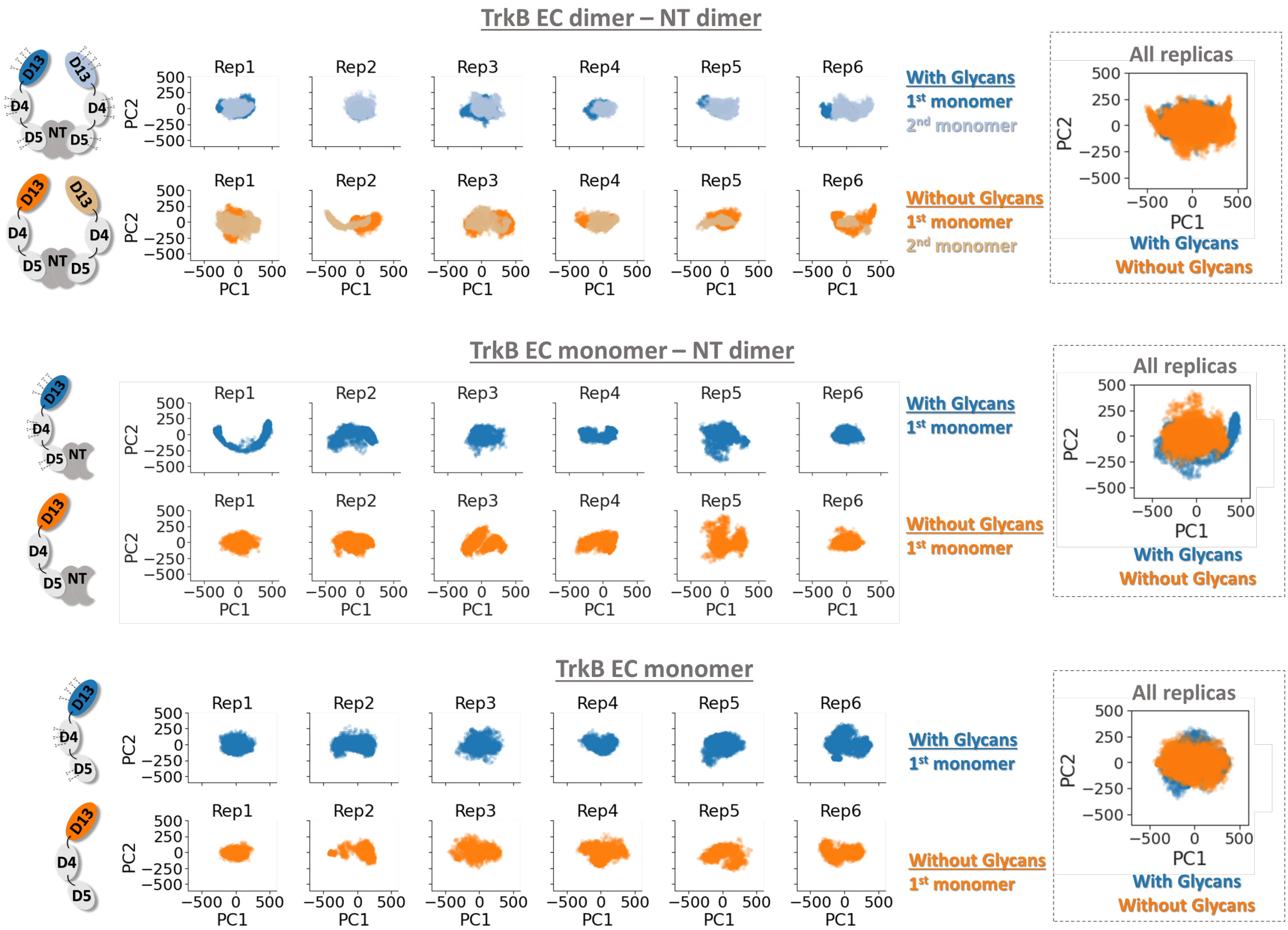

Figure S33: First and second principal components of the D13 domain during the simulations of the glycosylated and not TrkB systems. PCA analysis was performed to the D13 domains after alignment of each EC segment to the D4 and D5 domains. The color coding of each domain is shown in the schematic representations of the systems at the left of the plots.

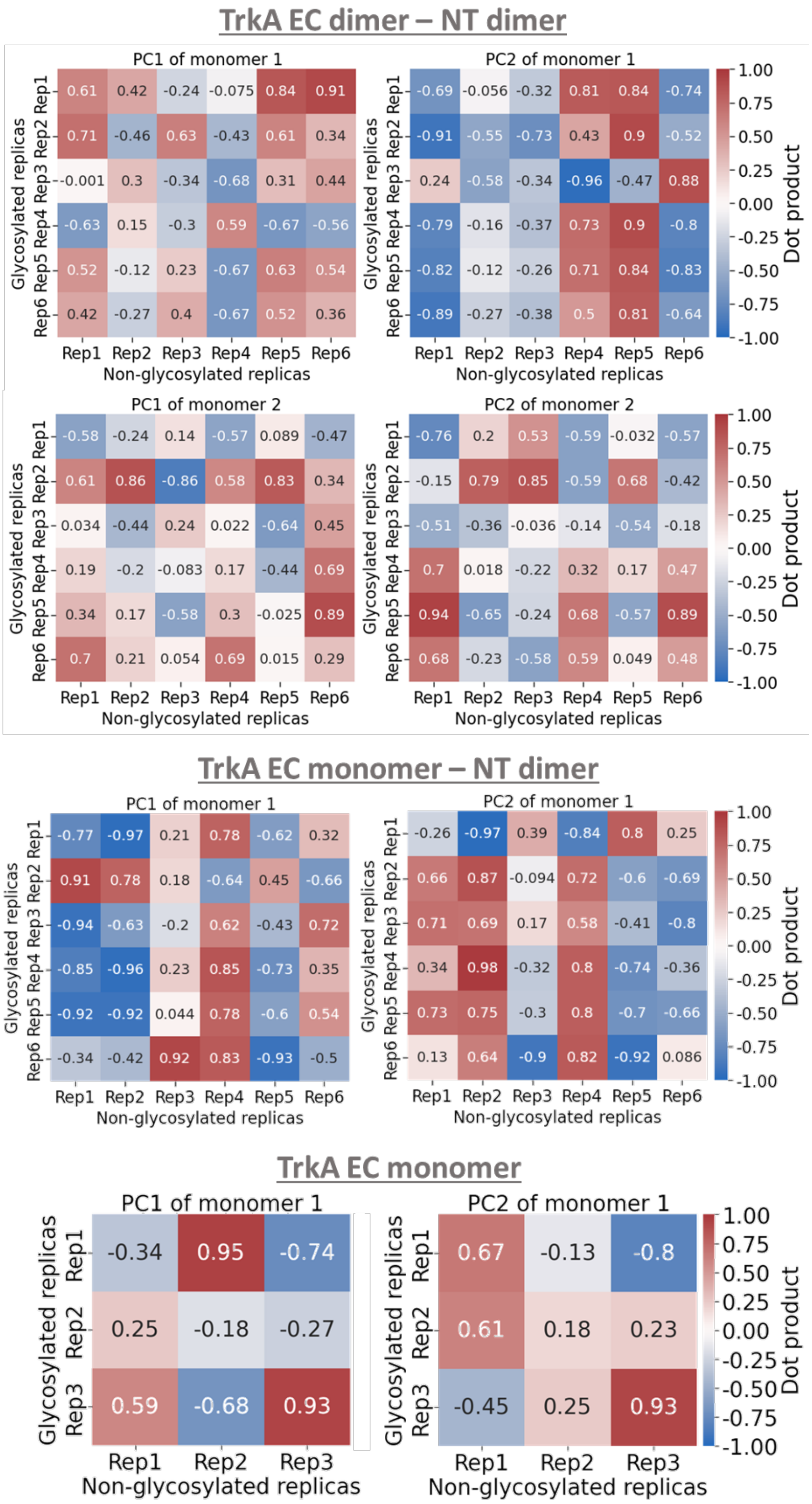

Figure S34: Calculation of the dot products of the first (PC1) and second (PC2) principal components between glycosylated and non-glycosylated replica pairs of TrkA systems. The PCA was performed on the D13 domain only, after alignment of the trajectories for the D4-D5 domains. In the systems of the receptor dimers, the PC1 and PC2 dot products were calculated for each monomer separately. The dot products are visualized as heatmaps, and the values mean: [1] parallel vectors, [-1] anti-parallel vectors and [0] vertical (or orthogonal) vectors. The analysis showes values close to zero for TrkA, indicating not so similar motions.

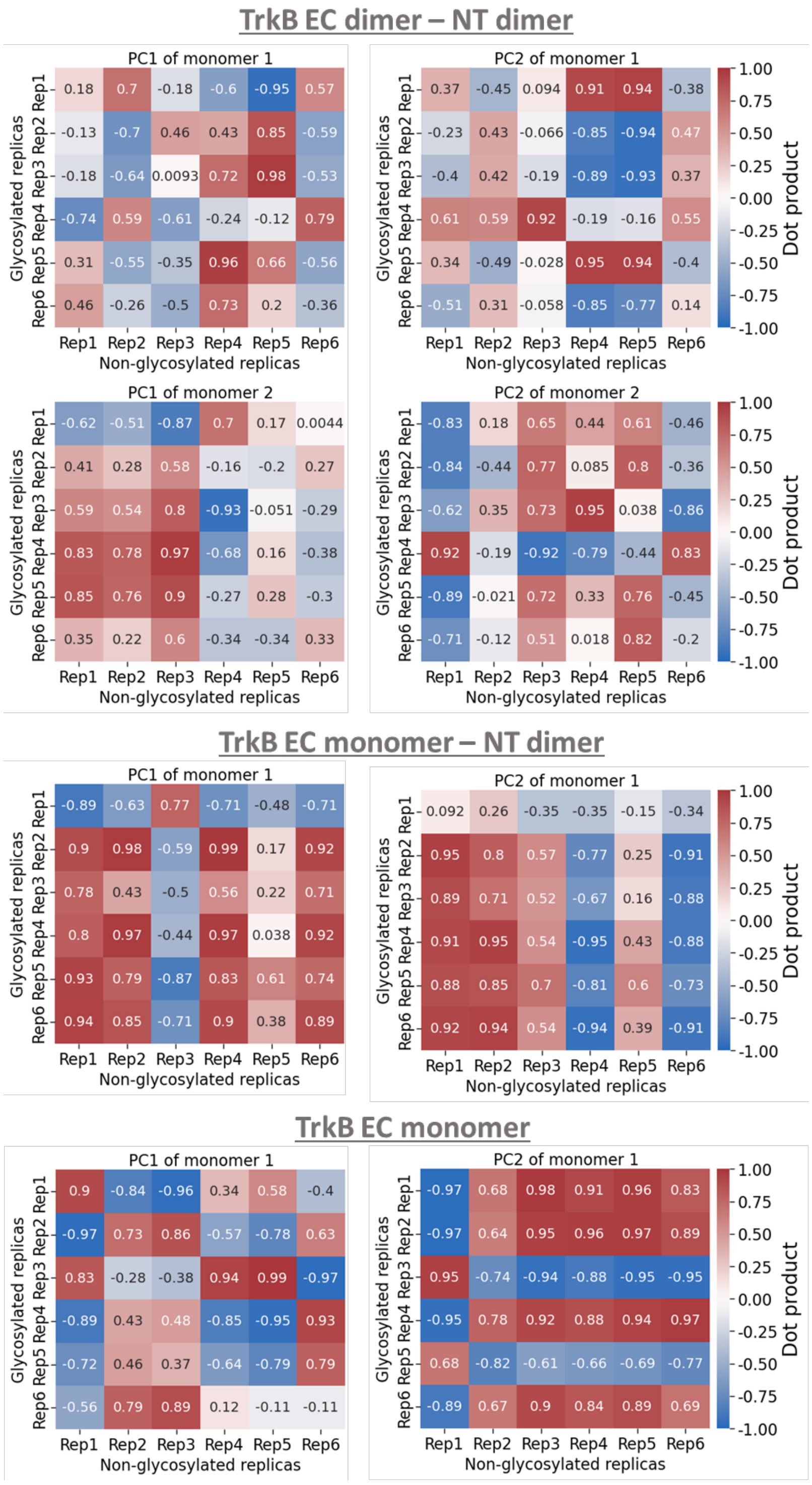

Figure S35: Calculation of the dot products of the first (PC1) and second (PC2) principal components between glycosylated and non-glycosylated replica pairs of TrkB systems. The PCA was performed on the D13 domain only, after alignment of the trajectories in the D4-D5 domains. In the systems of the receptor dimers, the PC1 and PC2 dot products were calculated for each monomer separately. The dot products are visualized as heatmaps, and the values mean: [1] parallel vectors, [-1] anti-parallel vectors and [0] vertical (or orthogonal) vectors. In contrast to TrkA, for TrkB, stronger similarities were observed with parallel or anti-parallel principal components, especially for the systems with receptor monomers. This behavior seems to coincide with the bigger differences observed between glycosylated and non-glycosylated forms for the TrkA systems compared to TrkB.

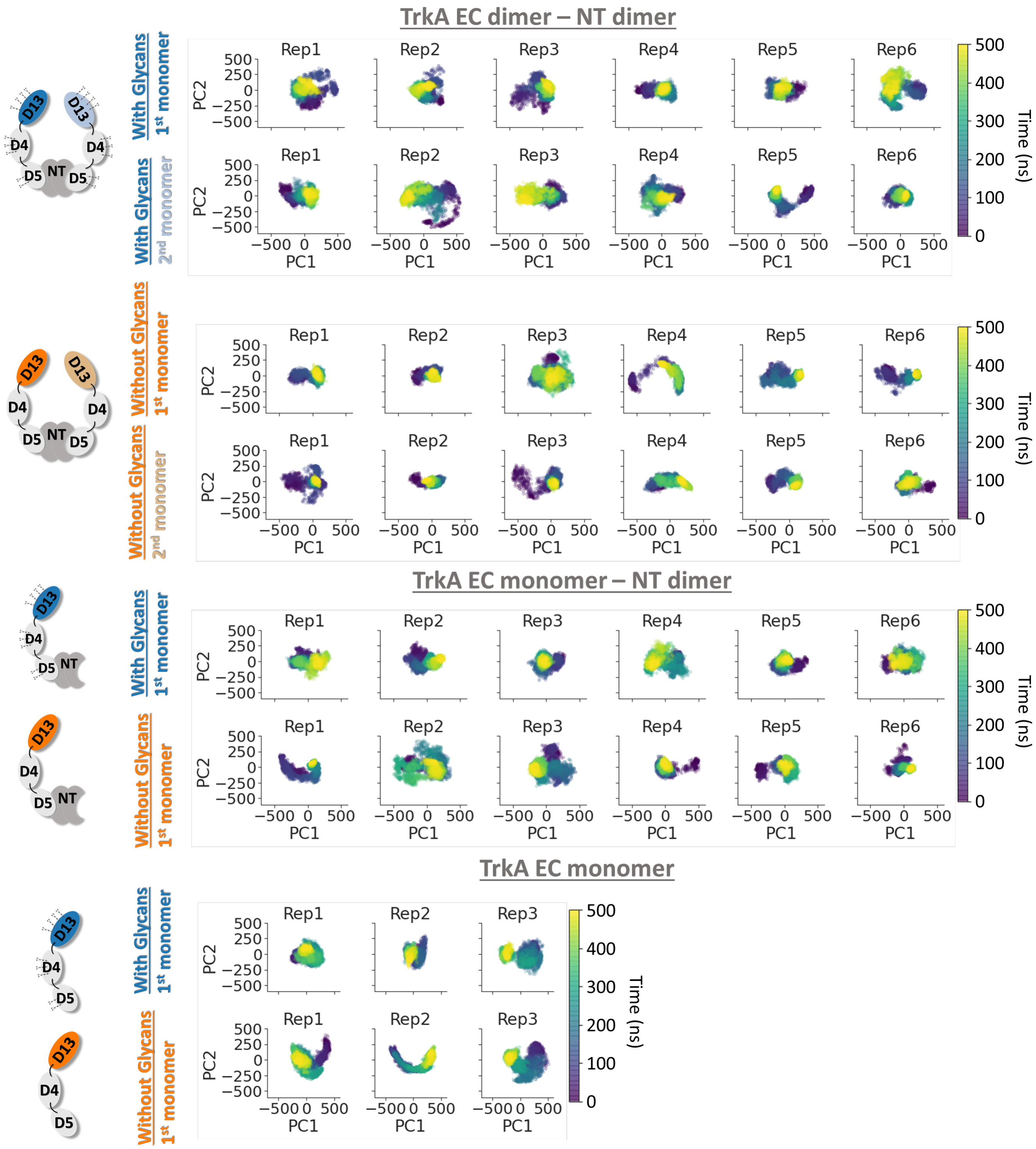

Figure S36: Time evolution of the first and second principal components of the D13 domain during the simulations of the glycosylated and not TrkA systems. PCA analysis was performed to the D13 domains after alignment of each EC segment to the D4 and D5 domains. The color coding of each domain is shown in the schematic representations of the systems at the left of the plots. The systems explore specific regions of the PC1 and PC2 space. In cases where some conformational change takes place during the simulations, and thus the system moves from one region to another in the PC1/PC2 space, no revisiting of the initial region takes place.

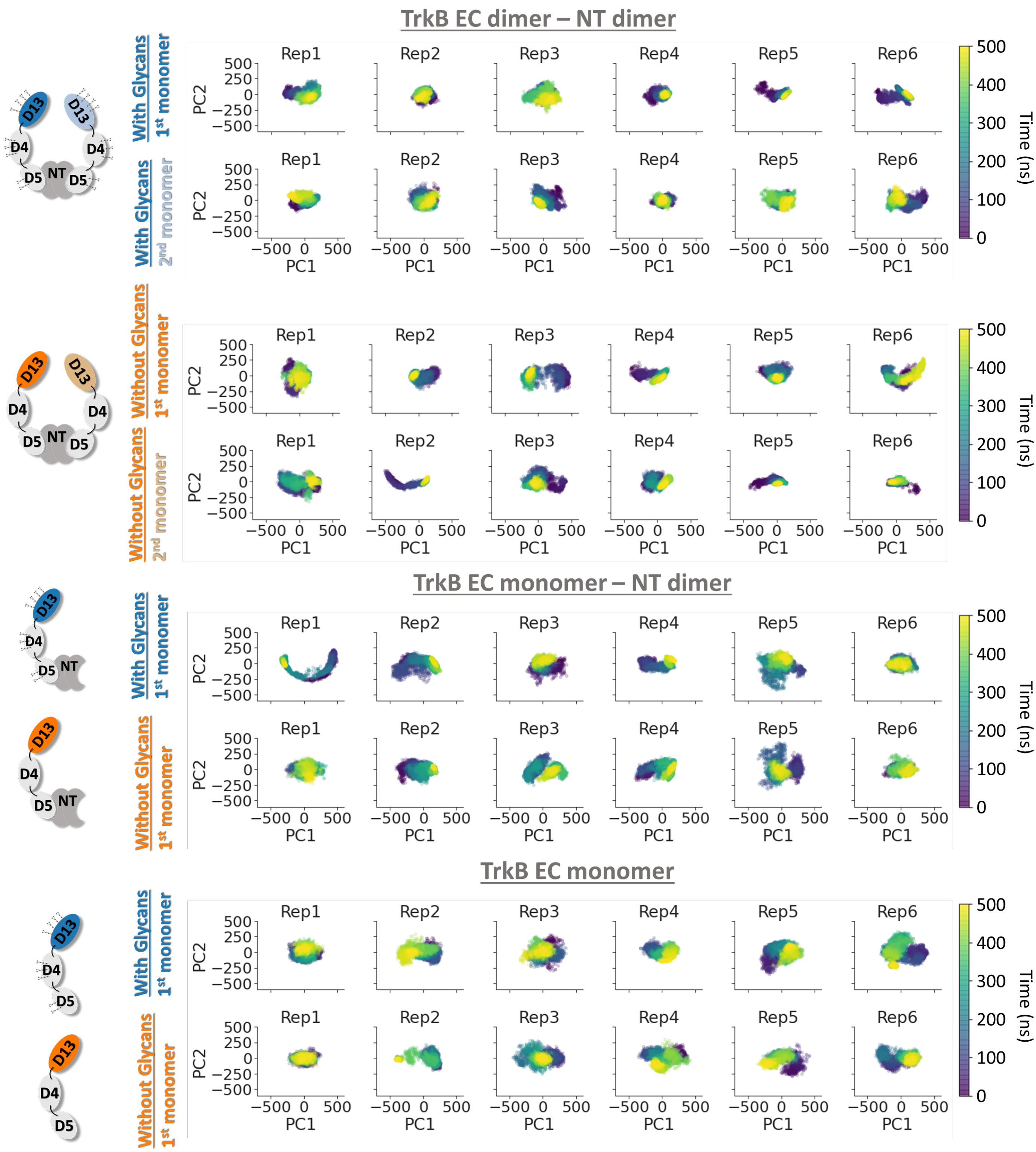

Figure S37: Time evolution of the first and second principal components of the D13 domain during the simulations of the glycosylated and not TrkB systems. PCA analysis was performed to the D13 domains after alignment of each EC segment to the D4 and D5 domains. The color coding of each domain is shown in the schematic representations of the systems at the left of the plots. The systems explore specific regions of the PC1 and PC2 space. In cases where some conformational change takes place during the simulations, and thus the system moves from one region to another in the PC1/PC2 space, no revisiting of the initial region takes place.

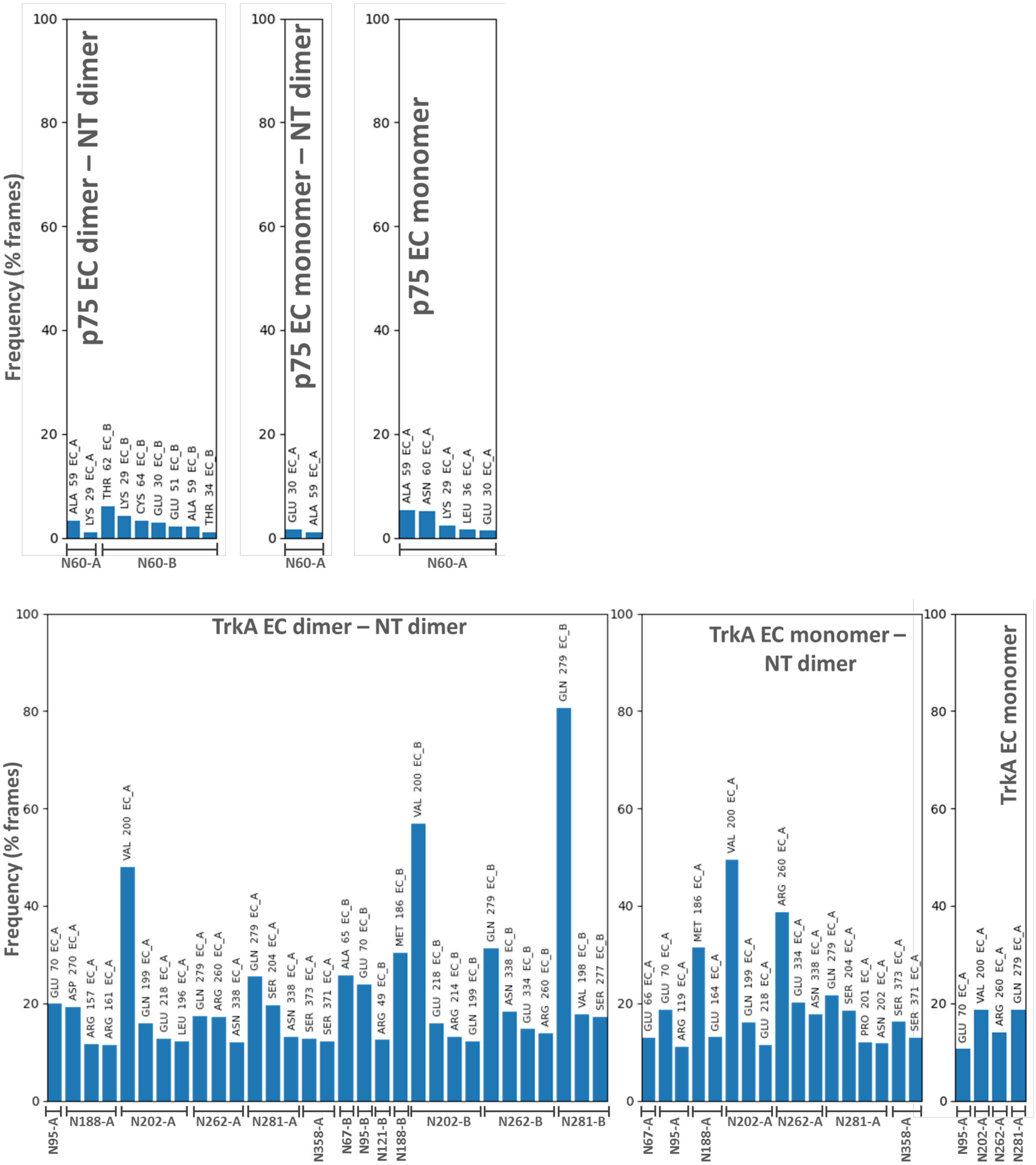
p75 EC dimer – NT dimer
p75 EC monomer – NT dimer
p75 EC monomer
Frequency (% frames)
ALA 59 EC_A
LYS 29 EC_A
THR 62 EC_B
LYS 29 EC_B
CYS 64 EC_B
GLU 30 EC_B
GLU 51 EC_B
ALA 59 EC_B
THR 34 EC_B
GLU 30 EC_A
ALA 59 EC_A
ASN 60 EC_A
LYS 29 EC_A
LEU 36 EC_A
GLU 30 EC_A
N60-A
N60-B
TrkA EC dimer – NT dimer
TrkA EC monomer – NT dimer
TrkA EC monomer
GLU 70 EC_A
ASP 270 EC_A
ARG 157 EC_A
ARG 161 EC_A
VAL 200 EC_A
GLN 199 EC_A
GLU 218 EC_A
LEU 196 EC_A
GLN 279 EC_A
ARG 260 EC_A
ASN 338 EC_A
GLN 279 EC_A
SER 204 EC_A
ASN 338 EC_A
SER 373 EC_A
SER 371 EC_A
ALA 65 EC_B
GLU 70 EC_B
ARG 49 EC_B
MET 186 EC_B
VAL 200 EC_B
GLU 218 EC_B
ARG 214 EC_B
GLN 199 EC_B
GLN 279 EC_B
ASN 338 EC_B
GLU 334 EC_B
ARG 260 EC_B
GLN 279 EC_B
VAL 198 EC_B
SER 277 EC_B
N95-A
N188-A
N202-A
N262-A
N281-A
N358-A
N67-B
N95-B
N121-B
N188-B
N202-B
N262-B
N281-B
GLU 66 EC_A
GLU 70 EC_A
ARG 119 EC_A
MET 186 EC_A
GLU 164 EC_A
VAL 200 EC_A
GLN 199 EC_A
GLU 218 EC_A
ARG 260 EC_A
GLU 334 EC_A
ASN 338 EC_A
GLN 279 EC_A
SER 204 EC_A
PRO 201 EC_A
ASN 202 EC_A
SER 373 EC_A
SER 371 EC_A
N67-A
N95-A
N188-A
N202-A
N262-A
N281-A
N358-A
GLU 70 EC_A
VAL 200 EC_A
ARG 260 EC_A
GLN 279 EC_A
N95-A
N202-A
N262-A
N281-A

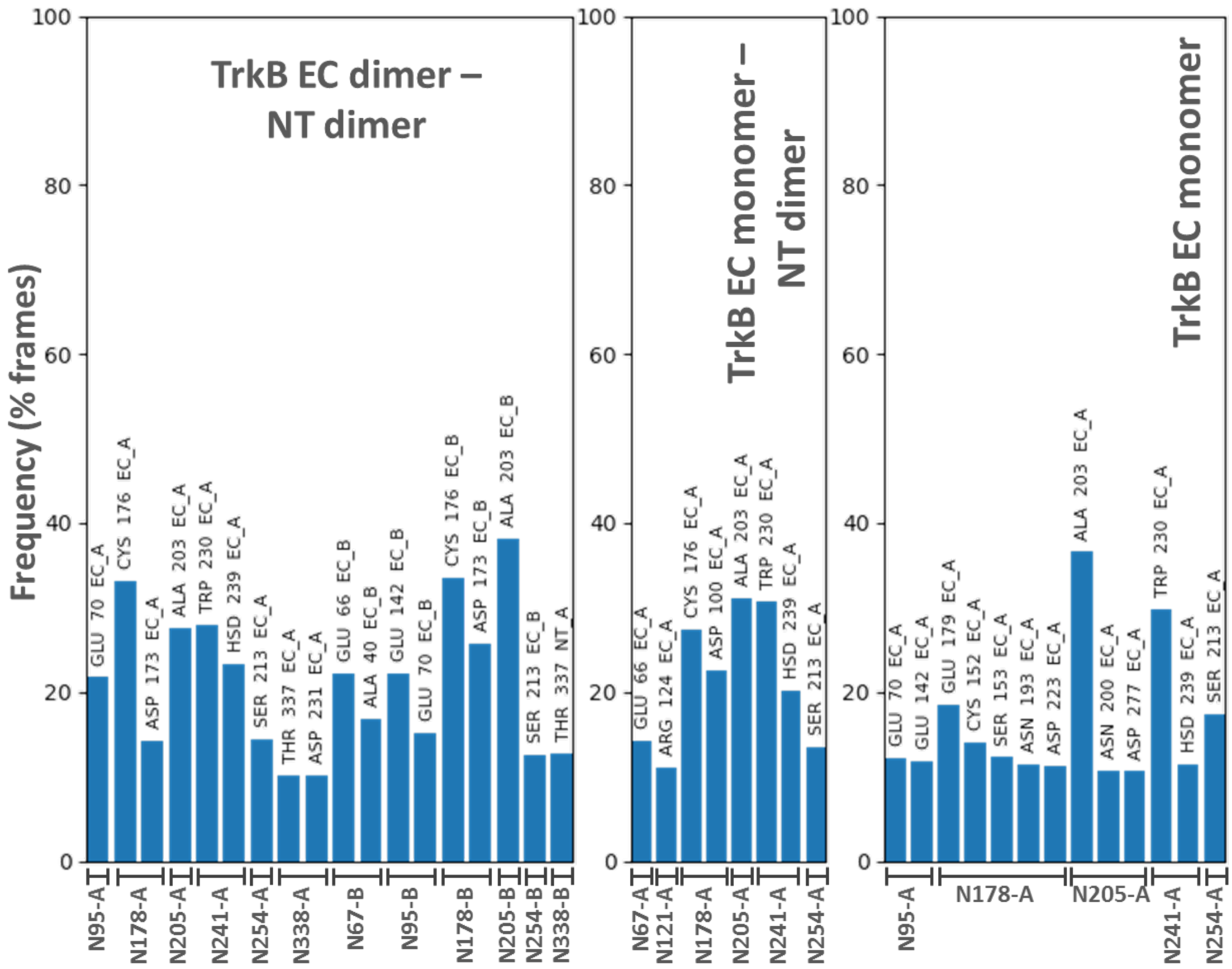

Figure S38: Hydrogen bonding analysis between the glycans attached in different Asn residues and the protein residues from all glycosylated p75, TrkA and TrkB systems. The glycans are indicated with the Asn residues from each receptor monomer (with A and B) in the x-axis, while the protein residues that interact with the glycans are indicated at the top of each bar. The height of the bars shows the percentage of the total simulation time in which the corresponding hydrogen bonding is present. Only hydrogen bonding of higher than 1% occupancy for p75, and 10% occupancy for the Trks is shown.

Supporting References

1. Haniu, M. *et al.* Extracellular Domain of Neurotrophin Receptor trkB: Disulfide Structure, N-Glycosylation Sites, and Ligand Binding. *Arch. Biochem. Biophys.* **322**, 256–264 (1995).
2. Haniu, M. *et al.* Interactions between Brain-derived Neurotrophic Factor and the TRKB Receptor. *J. Biol. Chem.* **272**, 25296–25303 (1997).
3. Wehrman, T. *et al.* Structural and Mechanistic Insights into Nerve Growth Factor Interactions with the TrkA and p75 Receptors. *Neuron* **53**, 25–38 (2007).
