# Supplementary material for: Glycosylation-modulated conformational diversity in neurotrophin receptors
Source: Biophys J. 2026 Apr 18;125(16):4407–19. doi: 10.1016/j.bpj.2026.04.017 (PMC13507394; doi:10.1016/j.bpj.2026.04.017)
Supplement: Supplementary file 2 [file mmc2.pdf]

Biophysical Journal
Article

Biophysical Society

Glycosylation-modulated conformational diversity in neurotrophin receptors

Alexandros Tsengenes,[1,2,3,6] Christina Athanasiou,[1,2,3,6] and Rebecca C. Wade[1,2,4,5,*]
[1]Molecular and Cellular Modeling Group, Heidelberg Institute for Theoretical Studies (HITS), 69118 Heidelberg, Germany; [2]Faculty of Biosciences, Heidelberg University, 69120 Heidelberg, Germany; [3]Heidelberg Biosciences International Graduate School, Heidelberg University, 69120 Heidelberg, Germany; [4]Center for Molecular Biology of Heidelberg University (ZMBH), DKFZ-ZMBH Alliance, 69120 Heidelberg, Germany; and [5]Interdisciplinary Center for Scientific Computing (IWR), Heidelberg University, 69120 Heidelberg, Germany
[6]These authors contributed equally

ABSTRACT Glycosylation is a widespread modification of cell-surface receptors, yet its structural impact is often overlooked due to the difficulty of experimentally characterizing glycans. Glycosylation of neurotrophin receptors has been reported to affect their localization and function. To investigate the effects of glycosylation of the extracellular domains (ECDs) of p75, TrkA, and TrkB neurotrophin receptors, we modeled their ECDs in glycosylated and non-glycosylated states and carried out molecular dynamics simulations of monomeric and dimeric forms of the ECDs with and without a neurotrophin bound. The single N-glycan on the p75 ECD provided minimal shielding and had limited interaction with the neurotrophin, although glycan-glycan contacts between the two p75 monomers may influence the stability of the receptor-neurotrophin complex. In contrast, TrkA and TrkB carry multiple N-glycans that shield the ECDs much more and, particularly for TrkB, increase the contact area between the receptor and the neurotrophin. The p75 ECD was comparatively rigid, independent of glycosylation state, likely due to its extensive network of disulfide bonds. In contrast, without glycans, the TrkA- and TrkB-ECDs tended to collapse inward, sometimes obstructing the neurotrophin binding site. Glycosylation of TrkA and TrkB prevented bending of the ECD into more compact states and instead promoted extended conformations that better accommodate neurotrophin binding. Overall, the simulations reveal distinct, receptor-specific roles of glycosylation in modulating neurotrophin receptor shielding, flexibility, and conformation with effects on neurotrophin binding.

SIGNIFICANCE Neurotrophins are proteins that promote neuronal survival. Their binding to the extracellular domains of transmembrane receptors leads to signal transduction intracellularly. Glycosylation of some neurotrophin receptors has been reported to affect their localization and function, but the mechanism is unclear. We investigate the effects of glycosylation on the structure, dynamics, and binding properties of the extracellular domains of three neurotrophin receptors by molecular dynamics simulation. We identify distinct receptor-specific effects of glycosylation. In particular, we find that glycosylation of tyrosine receptor kinases prevents concealment of the neurotrophin binding site due to receptor dynamics and favors a more extended receptor conformation, thereby supporting neurotrophin binding and the associated signal transduction.

INTRODUCTION

A common attribute of cell-surface receptors is the extracellular presence of covalently bonded oligosaccharides—glycans. The glycosylation sites are post-translationally modified during or after protein translation, and glycosylation may play a role in proper protein folding as well as affecting function and cell partitioning. Glycans can be categorized according to the amino acid to which the glycans are attached; e.g., N-glycans conjugate to the N atom of Asn residues, whereas O-glycans conjugate the O atom of Ser and Thr residues.[1] Due to the difficulty of identifying glycosylation sites and glycan types experimentally, the effects of glycans on protein structure and function are often neglected. Here, we therefore employ computational molecular modeling and simulation to explore the influence of glycosylation on the extracellular domains (ECDs) of three neurotrophin (NT) receptors.

NT receptors are single-pass transmembrane (TM) glycoprotein receptors with a key role in dendritic growth, synapse

Submitted November 30, 2025, and accepted for publication April 16, 2026.
*Correspondence: rebecca.wade@h-its.org
Editor: Frauke Graeter.
https://doi.org/10.1016/j.bpj.2026.04.017
© 2026 The Author(s). Published by Elsevier Inc. on behalf of Biophysical Society.
This is an open access article under the CC BY license (http://creativecommons.org/licenses/by/4.0/).

formation, neuronal survival, and apoptosis. NTs, the native ligands of these receptors, are homodimeric proteins that bind the ECDs of the full-length receptors and trigger a change in their structure that leads to receptor activation and intracellular signal transduction.[2] The ECDs bear glycosylation sites, which are known to impact the function of some of the receptors,[3] but the structural mechanisms and their potential influence on NT binding remain elusive. We focus here on the role of glycosylation in modulating the structure and dynamics of the p75, TrkA, and TrkB NT receptors. The p75 receptor is the non-specific (pan/death) receptor that can bind several types of NT between its ECDs, which are composed of four cysteine-rich domains with 12 disulfide bonds that form a disulfide ladder. TrkA and TrkB are specific receptor tyrosine kinases, with TrkA binding to nerve growth factor (NGF) and TrkB binding to brain-derived neurotrophic factor (BDNF) or NT-4/5. Their ECD is composed of five domains: domains 1 and 3 are cysteine rich, domain 2 is leucine rich, and domains 4 and 5 have an immunoglobulin G (IgG) fold. NT binding between the D5 domains of the two monomers in the receptor homodimer results in signal propagation through the TM domain and phosphorylation of the intracellular kinase domain.

The extracellular part of the p75 receptor has both N- and O-glycans, with a single N-glycan close to the N-terminal cysteine-rich domain and several O-glycans (the exact number is not known) on the flexible linker connecting the ECD and the TM domain.[3–10] In contrast, the Trk receptors have multiple N-glycosylation sites on their ECDs but lack O-glycans.[5,9,11] There is also evidence of glycosylation of the pro-domain of pro-NTs.[12–14] The single N-glycosylation of p75 has a complex glycan type,[4] while its O-glycans in the linker were found to contain sialic acids and have the core: Galβ1-3GalNAc-O-Ser/Thr.[15] Although O-glycosylation of p75 has been suggested to help with the proper folding of the EC linke[7,15] and the apical sorting of p75,[6–8] the role of the N-glycosylation of p75 is not clear, as its deletion does not have an effect on receptor cell partitioning.[3,6] In contrast, TrkA N-glycosylation is important for receptor localization to the plasma membrane and ligand-dependent activation.[3] When the receptor is not glycosylated, it is trapped intracellularly and it is constitutively active. Thus, it cannot trigger the Ras/Raf/MAP kinase cascade, which initiates in the plasma membrane, and consequently it cannot promote neuronal survival.[3]

Experimental structural biology methods, such as X-ray crystallography, often require removal of glycosylation from the protein structure during sample preparation to increase resolution, as glycans are very flexible and have a lot of structural variability and therefore tend to decrease the X-ray diffraction quality of the crystals or even hinder crystal formation.[16,17] This high flexibility renders the structural investigation of the effects of glycans on protein structure challenging. A solution is offered by molecular dynamics (MD) simulations, which enable explicit atomistic modeling of glycans and investigation of the dynamics of glycosylated proteins. Advances in mass spectroscopy have facilitated identification of protein glycosylation sites and the respective glycan types, providing a basis for modeling glycans.[18–21] Here, we modeled and performed MD simulations of the glycosylated and non-glycosylated ECDs of the human p75, TrkA, and TrkB receptors and compared differences in receptor structure and dynamics due to the presence of glycans. Our results indicate different effects and roles of the N-glycans in these three NT receptors.

METHODS

Protein modeling

For the modeling of the complex of the p75 receptor with NT-3, the crystal structure of the complex of the *Rattus norvegicus* (rat) p75-ECD homodimer and the human NT-3 homodimer was used (PDB: 3BUK).[22] The sequence identity with the human p75-ECD is ca. 92% and thus it was considered a good template. This structure contains the rat p75-ECD residues 32–190 (31–189 in human—corresponding to UniProt : P07174 and P08138, respectively), an N71S mutation in the rat p75-ECD, which results in the serine residue present in the human sequence, and the human NT-3 residues 143–253. It thus comprises (with a few missing residues) the full length of the structured parts of the p75-ECD and NT-3. This structure was processed using the Schrödinger Suite 2020[23] by mutating the residues that were different in rat to the human ones using Maestro. Then protein preparation was performed (bond order assignment, optimization of hydrogen-bond (H-bond) network, restrained energy minimization) using the Protein Preparation Wizard.[23] As the structure was missing the p75-ECD residues 29–30 and the NT-3 residues 139–142 and 254–257 in each monomer, MODELLER 9.23[24] was used for modeling them. The resulting model lacked hydrogen atoms, so the protonation states were predicted using PROPKA 3.4.0[25] via PDB2PQR 3.4.0.[26]

For the modeling of the TrkA receptor with NGF, the crystal structure of the complex of the TrkA full-ECD (residues 33–382) homodimer with the mature NGF homodimer (PDB: 2IFG) was used.[11] The structure of NGF was missing the following residues: Ser 122, Pro 182–Ser 187, Ala 237–Ala 241 (residue numbering of the UniProt sequence of human NGF; UniProt: P01138), which were built with the AutoModel class of MODELLER v.9.23, using default parameters. The linker consisting of residues Pro 182–Ser 187 has a conserved conformation in existing crystal structures and therefore it was modeled using the structure of human NGF (PDB: 5JZ7[27]) as a template. The ECD of TrkA has Ala 33–Pro 35 missing (code of human TrkA-II isoform, UniProt: P04629-1), which was built with AutoModel.

For the modeling of the TrkB receptor with NT-4/5, the full-ECD (residues 32–383) bound to the mature NT-4/5 was modeled. The crystal structure of the complex of the TrkB immunoglobulin-like domain 5 (D5) homodimer with the mature NT-4/5 homodimer (PDB: 1HCF[28]) was used together with the crystal structure of the complex of TrkA-ECD homodimer with the mature NGF homodimer (PDB: 2IFG) for the modeling of the D1–D4 domains. The full EC structure of TrkA was used as a template for TrkB, as both receptors have the same fold in their EC domains, i.e., two cysteine-rich regions (D1 and D3) flanking a leucine-rich repeat (known as D2) and two IgG-like domains (D4 and D5). The first three residues of the TrkB-D5 are cloning artifacts and were mutated to the wild-type sequence. The rest of the D1–D4 domain residues were modeled with AutoModel using the PDB structure (PDB: 2IFG) as a template. The structure of NT-4/5 is missing the following residues: Asn 145–Gly 150 and Gly 207–Ala 210 in chain A and Gly 81–Glu 84, Asn 145, Arg 209, and Ala 210 in chain B (residue numbering of the UniProt sequence of human NT-4/5, UniProt: P34130), which were modeled with the AutoModel class of MODELLER v.9.23. Additionally, the linker consisting of residues Asn 145–Gly 150 was built using the structure of human NT-4/5 from the PDB structure (PDB: 1B98[29]) as a template. Comparison of the D1–D4 domains from the TrkB model with the AlphaFold2 model (AlphaFold: AF-Q16620-2-F1-v6) of the full TrkB structure (pLDDT = 81.56), from the AlphaFold Protein Structure database[30,31] confirms the correct fold of the model and the validity of the structure (Figure S1). The D1–D4 domains that were modeled here have a median pLDDT score of 90.1 in the AF model, with very high scores of the D13 and D4 folded domains, and only the intermediate linkers have lower confidence (Figure S1). Structural alignments of the D13 and D4 domains from the homology models to the AF model show good agreement, with backbone root-mean-square deviation (RMSD) values of 2.12 and 1.65 Å, respectively (Figure S1).

For each receptor, three different systems were modeled: (1) one with the receptor ECD homodimer bound to NT, (2) one with one receptor ECD monomer bound to NT, and (3) one with the receptor ECD monomer alone. All systems were modeled with and without glycans (Figure 1; Table S1).

Glycosylation modeling

For p75, it is known that each ECD has one complex N-glycan of composition $Hex_5HexNac_4dHex_1NeuAc_2$ (Hex, hexose; HexNac, N-acetylhexosamine; NeuAc, N-acetylneuraminic acid) (Figure 1) at Asn 60.[4] p75 also has several O-glycosylation sites, which are located on the linker between the ECD and the TM domain.[6–8] However, here, only the structured ECD of p75 was modeled, so the O-glycosylation was not present.

The TrkA and TrkB receptors have N-linked glycans in their ECD.[3,9] Specifically, TrkA has 13 potential N-glycosylation sites, based on the Asn-X-Ser/Thr sequon, out of which at least nine are actually glycosylated.[3] Six of these sites are known from the crystal structure of the full TrkA-ECD bound to NGF.[11] These are the Asn residues at positions 95, 121, 188, 262, 281, and 358 (Table S2). In TrkB, there are 12 potential N-glycosylation sites of which 10 are glycosylated and their exact positions are known: Asn 67, 95, 121, 178, 205, 241, 254, 280, 338, and 412 (Table S2).[5,9] All glycosylation sites in TrkB are in the ECD, except for Asn 412, which is located in the linker between the D5 domain and the TM helix.

To decide on the positions of the glycan sites in TrkA that were not already known experimentally, the possible glycosylation positions in the TrkA structure were compared with the known positions in TrkB. The equivalent positions of the TrkA and TrkB glycosylation sites are given in Table S2. Glycan structures at four additional positions, Asn 67, 202, 253, and 401 (residues in bold font in Table S2), were built in TrkA, resulting in a total of 10 glycosylation sites, as for TrkB. No experimental information is available on the glycan types for either TrkA or TrkB except for an indication that sialic acid is present in the TrkA glycans.[32,33] Therefore, we decided to model the glycan root, $Man_3GlcNAc_2$, at all glycosylation sites on TrkA and TrkB that were present in the ECD, i.e., all glycans in Table S2, except for Asn 401 in TrkA and Asn 412 in TrkB, because these are located in the linker after the ECD, which was not modeled. For all systems, the glycans were built using CHARMM-GUI.[34]

MD simulations

All glycoprotein systems were inserted into an orthorhombic simulation box with CHARMM-GUI, solvated in TIP3P water with 150 mM NaCl, and then MD simulations were prepared and run using the CHARMM36m force field[35] and the GROMACS v.2020 MD engine.[36] The protocol consisted of a steepest-descent energy minimization, NVT and NPT equilibration steps with gradual removal of restraints, and one NPT production run. The NVT equilibration step was performed at 310 K for 500 ps with harmonic restraints with a force constant of 400 kJ/(mol·nm^2) on protein backbone atoms and harmonic restraints with a force constant of 40 kJ/(mol·nm^2) on the protein side chains. Then, the NPT equilibration was performed at 310 K and 1 atm for 500 ps with restraints on the protein backbone and side-chain atoms using force constants of 200 kJ/(mol·nm^2) and 20 kJ/(mol·nm^2), respectively. Finally, 3–6 replica production simulations of 500-ns duration were performed for each system without restraints and with different initial velocities for each replica (Table S1).

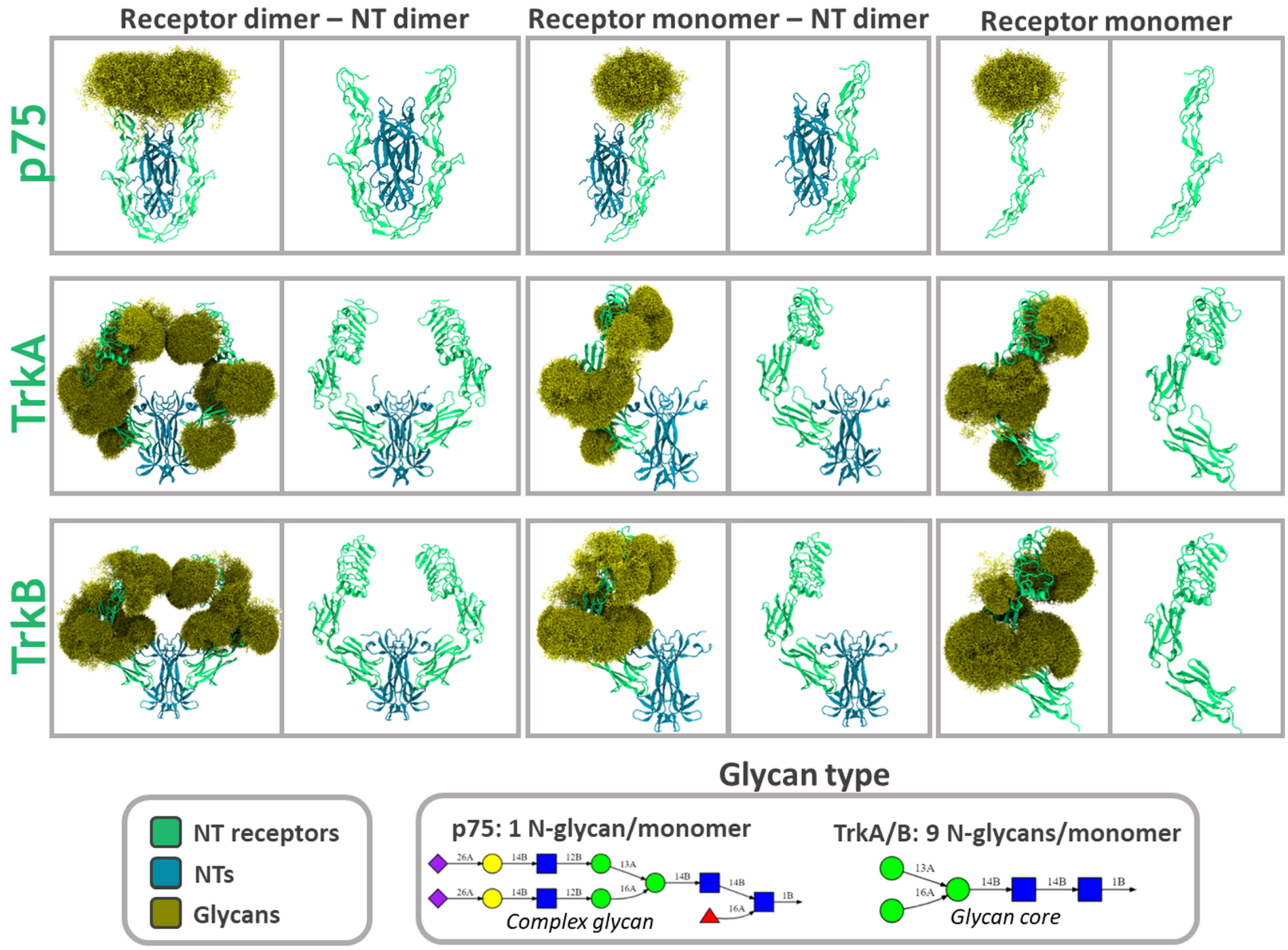

Figure 1. Overview of the modeled glycosylated and non-glycosylated p75 and Trk systems with the corresponding "glycan clouds" from the MD simulations. The systems are (1) the p75-ECD/TrkA-ECD/TrkB-ECD homodimer with the NT-3/NGF/NT-4/5 homodimer bound, (2) the p75-ECD/TrkA-ECD/TrkB-ECD monomer with the NT-3/NGF/NT-4/5 homodimer bound, and (3) the p75-ECD/TrkA-ECD/TrkB-ECD monomer. All systems were modeled and simulated with and without glycosylation. Proteins are shown in cartoon representation with the receptors in green and the NTs in blue. The systems with glycans present are shown with multiple glycan conformations (glycan clouds) superimposed from a set of time frames from the simulations. The glycans are shown in dark-yellow wire representation. The glycan types for p75, TrkA, and TrkB are also shown in Symbol Nomenclature for Glycans (SNFG) representation with the blue boxes denoting the N-acetyl-glucosamine, the green circles the mannose, the yellow circles the galactose, the purple rhombus the sialic acid, and the red triangle the fucose monosaccharides.

The Nosé-Hoover thermostat[37,38] and the Parrinello-Rahman barostat[39,40] were used with a coupling constant of 5 ps and isotropic pressure with $4.5\cdot\times 10^{-5}$ compressibility. The first equilibration step was run with a 1-fs time step and then a 2-fs time step was used for the second equilibration step and the production run, during which frames were saved every 100 ps. For the treatment of the non-bonded electrostatic interactions, the particle mesh Ewald (PME)[41,42] method was used with a cutoff distance of 1.2 nm, while, for the non-bonded van der Waals interactions, a cutoff of 1.2 nm and a switching distance of 1.0 nm were applied. All hydrogen-containing bonds were constrained with the LINCS algorithm.[43]

Simulation analyses

All simulations were post-processed with the *gmx trjconv* tool of GROMACS to remove periodic boundary condition jumps, reconstruct the molecules, and center the proteins in the simulation box. All trajectory frames were aligned to the initial structure using the whole protein backbone. Graphical rendering was done with VMD 1.9.3.[44] Properties were computed using the MDAnalysis python library[45,46] and plotted with the Matplotlib python library.[47] The Seaborn python library[48] was used to compute and plot distributions.

RMSD

The RMSD of the protein backbone was calculated for all systems using the MDAnalysis python library.[45,46] The RMSD was calculated for all frames of the trajectories, and the RMSD time evolution was plotted with the Matplotlib python library.[47] The distribution densities of the RMSD values were calculated and visualized with the Seaborn python library.[48] In addition, the internal RMSDs of all protein domains were calculated by aligning the trajectory to the backbone of each domain before the RMSD calculation for that particular domain.

Root-mean-square fluctuation

The root-mean-square fluctuation (RMSF) of the protein residues was calculated by first aligning the protein Cα atoms to an average structure from the simulations using the align.AverageStructure class of MDAnalysis, which aligns the trajectory to the first frame and then averages the coordinates. Then the RMSF of the Cα atoms of each residue was calculated using MDAnalysis and visualized with Matplotlib. The RMSF was also calculated for each glycan by aligning each ECD protein subunit to the first frame and then calculating the RMSF of each saccharide in the glycans. The RMSF values were plotted in boxplots with Seaborn.

Radius of gyration

The radius of gyration of the backbone of each receptor subunit in each trajectory frame was calculated with MDAnalysis. The radius of gyration distribution densities for the glycosylated and non-glycosylated systems were calculated and visualized with Seaborn.

Solvent-accessible surface area

Solvent-accessible surface areas (SASAs) were computed with the *measure sasa* tool of VMD 1.9.3.[44] The SASA values of the proteins (both receptor and NT) were computed every 100 frames with and without taking the glycans into account. The computed values were subtracted to obtain the area shielded by the glycans. Probe radii of 1.4, 2, 3, 4, 5, 6, 7, 8, 9, and 10 Å were used to emulate the interaction with molecules of different sizes, from water to small molecules and proteins.

Contact area between receptors and NTs

The contact area between the p75-ECD and NT-3, as well as the D5 domain of the TrkA and TrkB receptors with the NTs NGF and NT-4/5, respectively, was calculated with a probe radius of 1.4 Å using the *measure sasa* tool of VMD 1.9.3.[44] Specifically, the SASA of the domains of each receptor was calculated twice: with and without the NTs. Then, the two areas were subtracted to retrieve the receptor:NT contact area (Figure S2). For the simulations with the glycans, this calculation was performed with and without the glycans to identify any participation of the glycans in the contact area. These calculations were repeated for the individual receptor subunits of the receptor dimer systems.

Distances and angles between domains

For the receptor homodimers in complex with the NTs, the distances between the centers of geometry (COGs) of the domains of the subunits of the receptors were calculated. Specifically, for p75, the D1-D1, D2-D2, D3-D3, and D4-D4 distances were monitored, whereas, for the Trks, the D13-D13 (D13 = D1, D2, and D3), D4-D4, D5-D5, and D13-D5 distances were calculated. These distances were compared in the presence and absence of the glycans. Distances were calculated with MDAnalysis and the numpy.linalg.norm function of the Numpy library.[49] The same python libraries were used to compute the angles defined by the COGs of the D1-NT-D1, D2-NT-D2, D3-NT-D3, and D4-NT-D4 domains for p75 and the D13-NT-D13, D4-NT-D4, and D5-NT-D5 domains for the Trks. Both the distances and the angles were calculated every 100 frames. The data were post-processed with the Pandas library[50,51] and visualized with violin plots in Seaborn.

Principal-component analysis

Principal-component analysis (PCA) was performed to identify the intrinsic hinge motions of the D1 domain of the p75-ECD with respect to the D2–D4 domains and the D13 domains of the TrkA and TrkB receptors. All the simulation frames were aligned to the backbone of the D2–D4 domains of one p75 subunit or to the backbone of the D4-D5 domains of one Trk subunit. After the alignment, PCA was performed for the coordinates of the D1 of p75 and the D13 of the Trks using the built-in class in MDAnalysis. Plotting of the first and second principal-component values was performed with Seaborn.

H-bond analysis

The H-bonds between each glycan and the protein residues were calculated with the HydrogenBondAnalysis class of MDAnalysis for all frames of the trajectories. The results were post-processed with Pandas and Numpy and visualized with Matplotlib as the percentage of the frames in which each glycan forms H-bonds with specific residues. For the plotting, only the glycan-residue pairs that form H-bonds for more than 1% of the frames for p75 and 10% of the frames for the Trks were kept.

Dihedral angle (χ1) calculation

The χ1 dihedral angles of the asparagine residues that were glycosylated were calculated for all frames of all replica simulations of both the glycosylated and the unglycosylated systems using MDAnalysis. Plotting of the distributions of the χ1 values was done with boxplots in Seaborn.

RESULTS AND DISCUSSION

Glycosylated models of the ECDs of the p75, TrkA, and TrkB NT receptors

The effects of glycosylation of the ECDs of the p75, TrkA, and TrkB receptors were studied by modeling and

simulating the receptors with and without covalently attached glycans. Different oligomerization states of the systems were modeled: (1) receptor dimers bound to NT dimers, (2) receptor monomers bound to NT dimers, and (3) receptor monomers alone, all with and without glycans (Figure 1). Atomistic unbiased MD simulations were then performed for all systems.

The p75 NT receptor has a single N-glycan of complex glycan type[4] close to the N-terminal cysteine-rich domain and several O-glycans on the linker connecting the ECD and TM domain.[3–10] Here, only the single N-glycan of p75 at position N60 of the ECD was modeled (Figure 1).

In contrast to p75, the Trk receptors are known to have multiple N-glycosylation sites on their ECD but to lack O-glycan sites.[5,9,11] Specifically, TrkA has 13 potential N-glycosylation sites, based on the Asn-X-Ser/Thr sequon, out of which at least nine are actually glycosylated.[3] Six of these sites are known from the crystal structure of the TrkA-ECD bound to NGF (Table S2).[11] Of the 12 potential N-glycosylation sites in TrkB, 10 are glycosylated at known positions (Table S2).[5,9] All glycosylation sites in TrkB are located in the ECD, except for Asn 412 in the linker between the D5 domain and the TM helix. After comparison of the possible glycosylation sites in the TrkA structure with the known equivalent sites in TrkB, four additional glycosylation sites were modeled in TrkA (Table S2). Experimental data on the type of glycans in TrkA and TrkB are lacking, except for an indication that sialic acid is present in TrkA.[32,33] Therefore, the glycan root, $Man_3GlcNAc_2$, was modeled at all glycan sites on the TrkA and TrkB-ECDs (Figure 1).

Visualization of all the glycan conformational ensembles from the simulations reveals some clear differences between p75 and the Trks (Figure 1). For p75, the single complex N-glycan at N60 creates a glycan cloud that shields only the N-terminal part of the p75-EC, where it has minimal interactions with NT-3. On the other hand, the multiple glycosylation sites on the Trks shield several regions of their ECDs and participate in contacts with the NTs. Comparison of the TrkA and TrkB receptors shows that TrkA has glycans over the whole length of its ECD, whereas the glycans in TrkB mostly accumulate closer to its N terminus. The most important difference is that TrkB does not have any glycan shielding its D5 domain, whereas the glycan at Asn 358 in TrkA covers part of the D5 domain surface. The glycan conformational ensembles do not appear to be significantly influenced by the oligomerization state of the proteins, since no important differences in the glycan clouds are visible between the corresponding receptor monomers and dimers.

The cloud of glycan conformations in p75 appears to block the entrance for NT at the upper region of the receptor, which might affect NT binding and unbinding kinetics. Kinetic studies have shown that NTs dissociate from p75 at different rates, with NGF $>$ NT-3 $\gg$ BDNF.[52] Additionally, even though the Trks and p75 interact with NTs with similar dissociation constants in the nanomolar range, the binding kinetics are different, with NGF attaching to and dissociating from the Trks slower than p75.[53] The differences in the glycosylation profiles between p75 and the Trks could contribute to these kinetic differences.

The RMSD of the protein backbone with respect to the initial structures as a function of time was monitored (Figures S3–S5). For p75, there are no significant differences between the glycosylated and non-glycosylated systems, with only the NT-bound systems exhibiting slightly higher RMSD population values for the glycosylated states (Figure S3). Interestingly, the systems with the p75-ECD as a monomer alone show more fluctuations in the RMSD values, which suggests that the NT might stabilize the p75 ECD. Overall, the RMSD values are below 10 Å, which indicates deviation from the initial crystal structure without large conformational changes.

For the Trks, the RMSD profiles are similar for the glycosylated and non-glycosylated systems (Figures S4 and S5), with the "TrkA EC monomer-NT" and "TrkA EC monomer" systems showing slightly higher RMSD values for the non-glycosylated states. In contrast, the "TrkB EC monomer-NT" system displayed smaller RMSD values in its non-glycosylated state, as revealed from the RMSD distribution density peaks. Apart from these small differences, all systems display large RMSD values in the range of 10–30 Å, indicating large deviations from the initial models, which were based on crystal structures.

To examine the origin of these deviations from the starting structures, the RMSD values for the internal motions of each domain of the receptors were calculated by first aligning to the initial frame of each domain and then calculating the RMSD (Figures S6–S8). The results show much lower RMSD values than the global RMSD calculation—less than 3 Å for most of the domains—revealing that the domain structures are preserved during the simulations. In a few cases, higher RMSD values occurred, such as for the D1 domain of p75, for which the RMSD often exceeded 5 Å (Figure S6), accounting for the higher RMSD values for the full-ECD (Figure S3). Also, the D13-A domain in replica 1 of the "TrkA EC dimer-NT glycosylated" and replicas 1, 4, and 6 of the "TrkA EC dimer-NT non-glycosylated" systems have high RMSD values. Especially high RMSD values, of $\sim$10 Å, were observed in replica 2 of the "TrkA EC monomer-NT glycosylated" system (Figure S7). For the TrkB-ECD monomer, whether with or without glycosylation, the D13-A domain mostly had higher RMSD values than the other domains.

A closer look at the sampled conformations of these domains showed that most of the high values originate in the N-terminal linker of p75-D1, which is disordered and quite flexible. Interestingly, the region of the D1 domain where the N-glycan is attached has a more stable conformation,

although this also fluctuates (Figure S9). Overall, the rest of the p75 domains have a stable conformation despite containing several disordered regions. This stability is probably due to the 12 disulfide bonds in the p75 ECD, which form a disulfide-bond ladder (Figure S10). The high RMSD values on TrkA occur in the D13 domains due to changes in the N-terminal region (Figures S11 and S12). The D13 domains have several disordered regions, and the N-terminal part undergoes different rearrangements during the simulations. This is in agreement with the high B-factors of this N-terminal region in the crystal structure of the TrkA-ECD bound to NGF (PDB: 2IFG)[11] (Figure S13). Apart from the D13 domains, the D4 domain also sustains conformational changes, especially in the linker between the D and E strands, leading to an RMSD of 10 Å in one replica (Figure S12). Overall, most of the high RMSD values occur for the non-glycosylated systems rather than the glycosylated ones. In general, the RMSD values of the individual domains (D1–D4) are much lower than those of the global RMSD, indicating that the high RMSD values originate in the linker (or hinge) regions between the domains.

Analysis of residue fluctuations during the simulations showed that the glycosylated and non-glycosylated p75, TrkA, and TrkB systems behave very similarly (Figures S14 and S15) with only the N-terminal region of p75-D1 and the N- and C-terminal residues of the TrkA/TrkB-ECDs showing higher RMSF values, since they have more disordered structures. Also, in the TrkA EC monomer simulations, the D4 domain on average fluctuates more in its non-glycosylated state (Figure S15C, TrkA). Most notably, the glycans do not appear to influence the intrinsic fluctuations of the Asn conjugation sites as the RMSF values for the glycosylated and non-glycosylated systems are generally the same at these residues. The RMSF values of the NT residues are smaller than those of the receptors, except for the N- and C-terminal regions, which are more mobile because they correspond to unstructured, flexible loops. Also, the glycosylation of the receptors does not seem to affect RMSFs of the NTs over the simulations.

Differential glycan shielding on p75 vs. Trk receptors

The ability of the glycans to bury parts of the receptor surfaces was assessed. As has previously been demonstrated by MD simulations, glycans can shield large parts of the protein area exposed to solvent or other molecules.[18] Therefore, we calculated the accessible surface area (ASA) of the receptor ECDs, with and without glycans, for different probe radii representing molecules of different sizes ranging from 1.4 Å, which corresponds to a water molecule, to 9 Å, which represents a larger molecule, such as a protein (Figures 2A and S16). Intermediate values correspond to the size of small molecules (∼3–4 Å) or oligopeptides (5–7 Å).

Comparison of the glycan shielding among the three receptors showed that the area buried in p75 by the single glycan is much smaller than the area buried in the Trks by glycans at the nine glycosylation sites, despite the much longer structure of the complex glycan on p75. The reason is that the glycans at N60 on p75 point away from the surface of the protein and tend to interact with each other, potentially contributing to the avidity of the whole complex. Comparison of the TrkA and TrkB receptors shows a similar degree of shielding even though the glycans are attached at different positions on the receptors. This means that the size of the glycans and their total number, which are the same for both receptors, determine the amount of shielding.

This analysis also shows that increasing the size of the probe leads to greater glycan shielding, up to 7% of the receptor ECD ASA for p75 and 28% for the Trks, meaning that the binding of larger proteins may be more disturbed by the glycans than the binding of small molecules. This 4-fold increase in glycan shielding for the Trks compared to p75 might be important for their protection against proteolytic cleavage by, e.g., secretases, which has been observed physiologically for p75.[54]

To better understand the origin of the glycan shielding, the intrinsic fluctuations of the glycans were calculated for all receptor systems. For this purpose, each of the domains that have glycans were first aligned to the first frame and then the averaged RMSF values of all monosaccharides of each sugar were calculated (Figures 2B and S17). The glycan on p75 exhibited an average fluctuation of 10 Å with a very narrow distribution, while, for the Trks, higher RMSF values were observed for the glycans that are located closer to the N terminus of the ECD. This behavior is in agreement with the lower shielding observed for p75, as higher glycan fluctuations would be expected to effectively bury more receptor surface.

To examine the effects of the glycans on the conformations of the side chains of the conjugated Asn residues, the $\chi 1$ dihedral angles of these residues were calculated (Figures 2C and S18–S20). For p75, no significant difference was observed for the N60 residue in the presence or absence of the complex glycan, whereas, for the Trks, a more restricted $\chi 1$ space was observed when the Asn residues were glycosylated, suggesting that specific conformations of the side chains are adopted upon glycosylation.

Finally, the influence of the glycans on the contact area between the receptors and the NTs was assessed. The contact area was calculated as the difference in the ASA of the ECD with and without the NT present. In the systems where glycans were present, the calculation was performed with and without considering the glycans attached to the D5 domain (Figures 2D and S21). Comparison of the contact area between receptor and NT with and without taking into consideration the presence of glycans shows exactly the same results for p75, suggesting that the N-glycan does not interact with NT-3, whereas, for TrkA, and especially TrkB, the

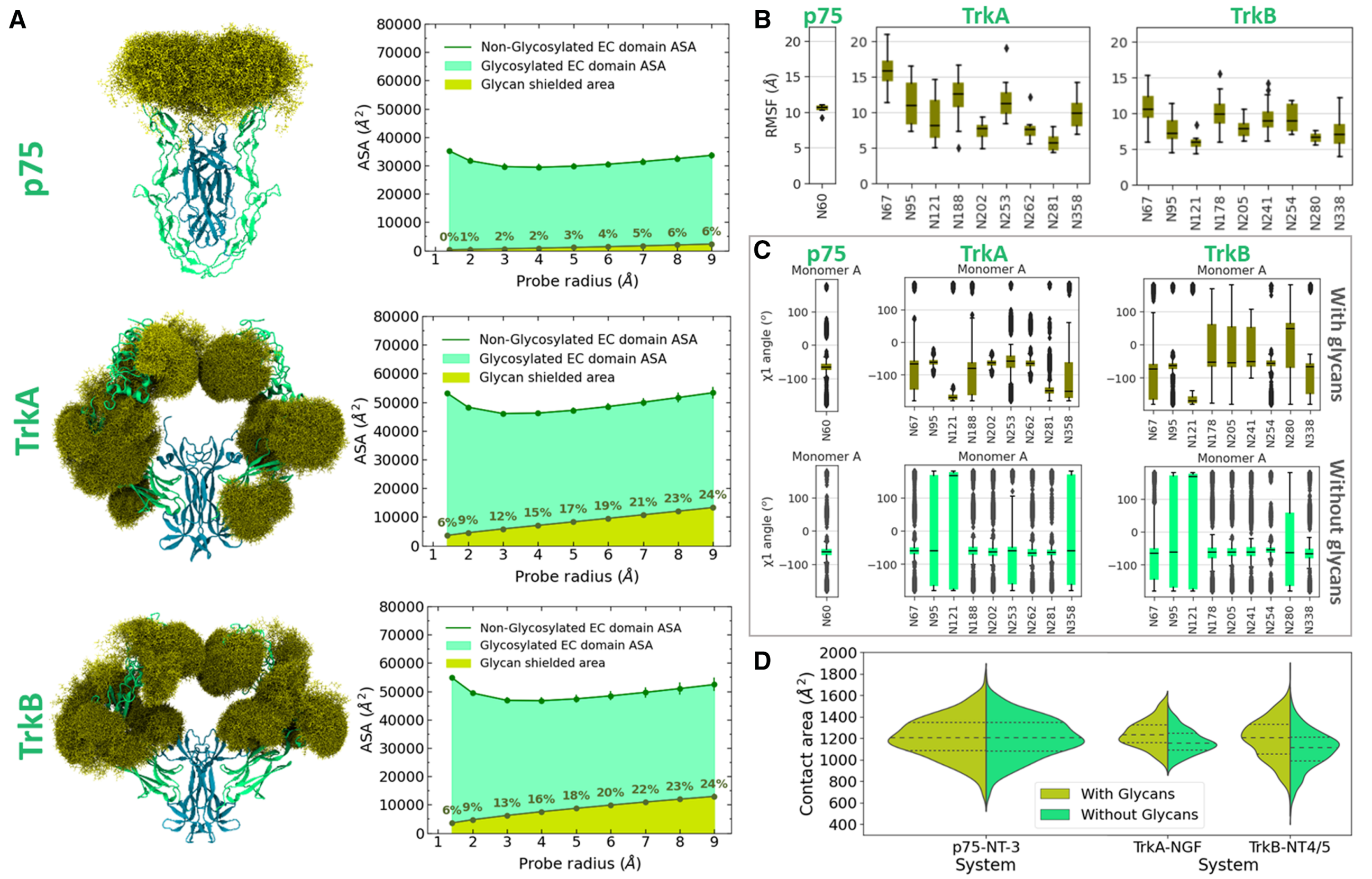

Figure 2. Analysis of shielding and structural changes due to glycosylation for the p75 and Trk "receptor dimer/NT dimer" systems. (A) Accessible surface area (ASA) of the p75, TrkA, and TrkB systems (including the ASA of the NTs when present) and the area shielded by glycans computed with multiple probe radii ranging from 1.4 Å (water molecule) to 9 Å (protein-sized molecule). The values were calculated and averaged across all replicas with glycans. The area shielded by the glycans is shown in yellow (with rounded % values), and the green line shows the accessible area of the protein in the absence of glycans. The area highlighted in green remains accessible in the presence of glycans. (B) Distributions of RMSF values for all glycans attached in the Asn (N) residues in the glycosylated p75, TrkA, and TrkB systems. (C) Distributions of the χ1 dihedral angle of the glycosylated Asn residues in the glycosylated and non-glycosylated systems. (D) Distributions of the contact area between the receptors and the NTs in the glycosylated and non-glycosylated systems. The quartiles of the distributions are indicated with boxes (B,C) and short dashed lines (D) around the medians which are indicated by horizontal lines (B,C) and long dashed lines (D). Whiskers (B,C) indicate the lowest and highest data points within 1.5 times the interquartile range of the quartiles and outliers beyond this range are plotted.

contact area is higher when considering the glycans, showing that they interact with the NTs. This difference in TrkB is probably due to the glycan attached to Asn 338, which is in close proximity to the NT and is present in TrkB and not TrkA. Comparison of the ECD dimer with the monomer systems shows that the contact area per subunit is smaller for the TrkA monomer, while, for p75 and TrkB monomers, it is similar to that for the dimer (Figure S21). This shows that the packing between NGF and TrkA is tighter in the dimeric system, indicating the importance of receptor dimerization for complex formation with NGF.

Glycosylation promotes extended conformations of the ECDs of the TrkA and TrkB receptors

The effects of glycosylation on the conformations of the ECDs of the p75, TrkA, and TrkB receptors was investigated by calculating the radius of gyration of each receptor subunit. Comparison of the distributions of the radius of gyration of the glycosylated and non-glycosylated systems reveals that the non-glycosylated TrkA and TrkB systems sample conformations of smaller radius of gyration, indicating that the ECD conformations become less extended and more compact, whereas, for p75, no difference was observed (Figures 3A and S22–S24). The apparent stability of the p75 conformation suggests greater rigidity of the structure of the p75-ECD, likely due to the presence of the disulfide bond ladder (Figure S10).

The extension of the conformations in the presence of glycosylation was more pronounced in the TrkA systems. In the absence of glycosylation, the simulations, which started from the extended crystal structures, showed contraction of the ECDs, allowing sampling of bent conformations. The crystal structure of the ECD of TrkA dimer bound to NGF shows the first three saccharides of the glycans, and thus these may be sufficient to promote the extended conformation of the ECD. Comparison of TrkA with TrkB shows that the effect of glycosylation is less

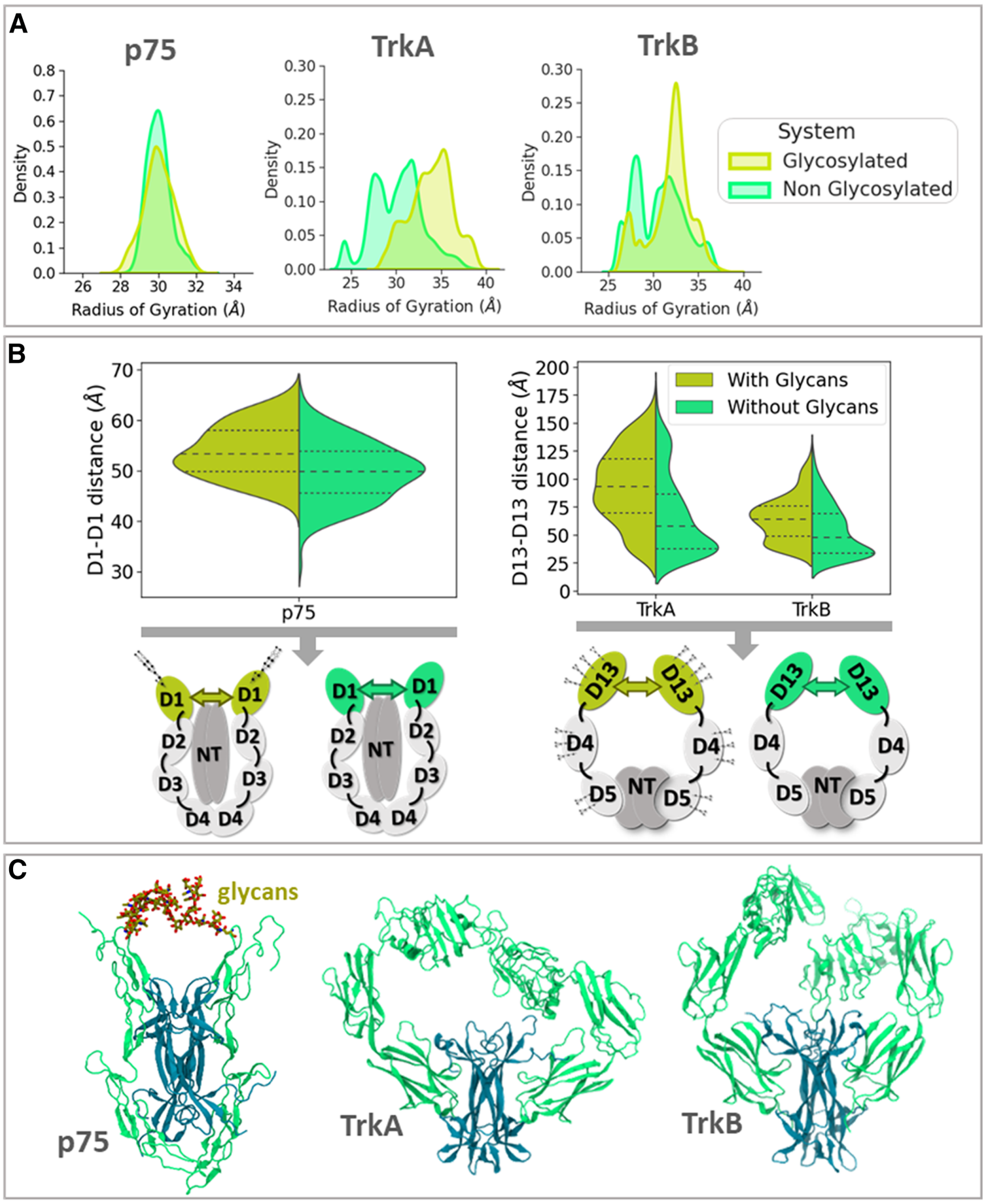

Figure 3. Conformational effects of glycosylation on the p75 and Trk receptors. (A) Distributions of the radius of gyration of the receptor ECD backbone of the glycosylated and non-glycosylated p75, TrkA, and TrkB systems. (B) Distributions of the D1-D1 interdomain distances for p75 and the D13-D13 interdomain distances for the Trks. The quartiles of the distributions are indicated with short dashed lines above and below the median which is indicated by long dashed lines. (C) Representative structures showing interactions between the complex glycans at N60 of the two p75-ECD subunits and bent ECD conformations of the non-glycosylated TrkA and TrkB systems.

apparent for TrkB. Analysis of the radius of gyration density distributions reveals that glycosylated TrkB samples a broader range of conformations, including those with smaller radii of gyration (<30 Å), whereas glycosylated TrkA only has conformations with a higher radius of gyration (>30 Å).

To quantify the hinge motions, the distance between the D1 domains for p75 and the D13 domains for the Trks was computed (Figure 3B). The average values of these interdomain distances are lower for the non-glycosylated systems, with the Trk systems showing a greater difference. The distance distributions between the rest of the domains, as well as the angles among the two domain pairs and the NTs, were also calculated, showing that, for p75, the D2-D2 and D4-D4 interdomain distances are also a little lower for the non-glycosylated systems, while, for TrkA, a smaller average D4-D4 interdomain distance was observed in the absence of glycans (Figures S25 and S26). The angles follow similar patterns to the distances. These results indicate that the two p75-ECD subunits are somewhat more distant when the two N-glycans at N60 are present, as the two glycans are accommodated between the two p75-ECD subunits (Figure 3C-p75). The glycans in this conformation interact with each other and can potentially block the NT from dissociating from p75, which might affect its binding kinetics. Also, the interacting glycans might enhance the overall avidity of the complex and stabilize it in the homodimeric state. The smaller distances between the D13 domains of the Trks is consistent with the smaller radius of gyration for the non-glycosylated systems and corresponds to bent, closed conformations of the receptors (Figure 3C, TrkA/B; Figure S28). The bent conformations of the Trks were also quantified by the distributions of the D13-D5 interdomain distance (Figure S27), which sample smaller values for all non-glycosylated systems, with this effect being more apparent in TrkA than TrkB.

PCA was performed to investigate the main motions of the proteins. For p75, the principal components (PCs) of

the movement of the most mobile domain, the D1 domain of the p75-ECD, with respect to the D2–D4 domains was computed (Figures S29 and S31). Comparison of the first and second PCs for the glycosylated and non-glycosylated systems showed similar behavior. The "p75-ECD monomer" system covered more PC1 space than the other systems, which might be due to the increased fluctuations mentioned previously (Figure S14). PCA was also performed on the coordinates of the D13 domains of TrkA and TrkB, as this region showed the largest movements (Figures S32 and S33). As for p75, comparison of the principal motions of the glycosylated and non-glycosylated systems shows that they cover mostly the same PC1 and PC2 space.

Finally, an analysis of the H-bonding between the glycans and the protein residues was performed (Figure S38). This analysis shows that, for p75, the Asn 60 glycan makes few H-bonds, most frequently with the adjacent Ala 59. For TrkA, the interaction between the glycan at Asn 202 and Val 200 occurs with >10% occupancy in all systems and subunits. The glycan at Asn 281 interacts with Gln 279 in all TrkA systems. Indeed, this interaction has ca. 80% occupancy in the second subunit of the TrkA dimer system. Apart from these interactions, the glycans form H-bonds with several Glu, Arg, and Gln residues in all TrkA systems. For the TrkB systems, the H-bonds mostly had lower occupancy compared to TrkA. Among the most stable interactions in all systems and subunits was that between the glycan at Asn 205 and Ala 203 of TrkB. The interaction between the glycan at Asn 241 and Trp 230 also occurred with >20% occupancy. Many glycans interact with Glu and Asp residues on TrkB.

Overall, the analysis of H-bonds between the glycans and the protein residues of the Trks did not reveal any very specific interactions, and the glycans mostly interact with their local environment rather than any distant residues. This might be because of the conservative modeling of the glycans with only the first five saccharides (the glycan core). Perhaps the long antennae that are normally present on the glycans under physiological conditions would be able to form more specific interactions. On the other hand, since there is usually a lot of variability in the glycan type according to the cell type, different structures of glycans can be found at a single position. Therefore, one would not expect specific interactions of the receptor with a particular type of glycan structure, as this might not be conserved. Thus, the glycans are most likely to affect the receptor conformations through steric contacts rather than specific H-bonding interactions.

CONCLUSIONS

The effects of glycosylation on the structure and function of cell membrane receptors are often neglected due to the challenges of characterizing glycosylation experimentally. Here, we modeled the glycosylation on the ECDs of the p75, TrkA, and TrkB NT receptors and performed MD simulations to assess the influence of glycosylation on the receptor structure and dynamics by comparing glycosylated with non-glycosylated states of the receptors. The three receptors were modeled in three different forms: (1) the receptor ECD homodimer with an NT bound, (2) the receptor ECD monomer with an NT bound, and (3) the receptor ECD monomer alone. For p75, it was known that the ECD has a single N-glycan of complex chemical type, which was modeled as such. The TrkA and TrkB receptors, on the other hand, have multiple N-glycosylation sites, which are known for TrkB and partially known for TrkA. Therefore, we defined the most plausible sites on TrkA by comparison with TrkB. Since the exact glycan types on the TrkA and TrkB receptors are not known, we modeled only the N-glycan core (i.e., the first five saccharides, which are common to all N-glycans).

While glycans are well known to shield protein surfaces, unbiased MD simulations for the glycosylated receptors showed that the extent of shielding differs widely among the three NT receptor ECDs. Only the N-terminal part of the p75-ECD could be shielded by the single N-glycan at the Asn 60 position, which also made only minimal interactions with NT-3. TrkA has glycans over the full length of its ECD, whereas the glycans in TrkB mostly accumulate in its N-terminal region. Another important difference is that there is no glycan shielding of the TrkB D5 domain (the domain that interacts with the NT), whereas TrkA has a glycan at residue Asn 358 that shields the solvent-exposed surface of the D5 domain.

The ensemble of glycan conformations occupies the intersubunit space of the upper region of the p75 receptor and might thereby affect NT binding kinetics, which have been found experimentally to differ for the p75 receptor compared to the Trk receptors.[53] Glycan shielding was quantified to be minimal for p75 (only up to 7%), while, for the Trks, it reached ca. 30% of the receptor ECD surface area for large probes, suggesting binding of larger proteins, such as antibodies, rather that solvent or small molecules, might be most affected by the presence of glycans. This may be relevant for protection of the receptors from proteolytic cleavage by, e.g., secretases, which occurs physiologically for the p75 receptor.[54] Another interesting observation was that the glycosylation in the Trk receptors, especially TrkB, formed contacts with the NT, increasing the contact area between receptor and NT, which was not observed for p75.

Investigation of the effects of glycosylation on the conformations of the receptors showed that the glycans promoted the extended conformations of the TrkA and TrkB receptors, while the conformation of the p75-ECD was particularly stable, most probably due to the ladder of 12 disulfide bonds in its structure. Visualization of the TrkA and TrkB trajectories showed that, when the glycans are absent, the ECD dimers bend and the two subunits interact with each other or with the NT, whereas, in the ECD

monomers, the D13 domains interact with the D5 domain. Similar closed conformations of the ECDs have been reported before from enhanced sampling simulations.[55] Smaller D13-D5 interdomain distances occurred for the non-glycosylated systems, providing direct evidence of the more closed conformations of the non-glycosylated receptors. This was more apparent for the TrkA systems, but the non-glycosylated TrkB systems also seem to explore more conformations with smaller interdomain distances than when glycans are present. For p75, longer D1-D1 interdomain distances occurred in the glycosylated form, likely because the two glycans were accommodated between the two p75-ECD subunits.

Finally, calculations of H-bonds between the complex N-glycan at Asn 60 of p75 and the protein residues revealed interactions mostly with the adjacent Ala 59, as well as Lys 29 and Glu 30. In the homodimeric p75-ECD simulations, the glycans from the two monomers interacted with each other, which could stabilize the receptor dimer and contribute to its avidity and, furthermore, hinder the NT from dissociating from p75 and affecting its binding kinetics. For the Trks, H-bond analysis did not reveal any important specific interactions, suggesting that the reason for the glycans promoting the extended conformations of the receptors is mostly due to steric hindrance of the closed conformations when the glycans are present. This is in agreement with the fact that many of the glycans are attached at the linkers of the Trks between the subdomains or close to them. Thus, the glycans can limit the hinge motions of the receptors and keep them in more extended conformations, which might facilitate NT binding. In the systems where the NT and glycans were not present, the D13 domains were able to bend and interact with the D5 domain, which is the binding site of the NT. This bending could hinder NT binding under physiological conditions or reduce the rate of binding and thus receptor activation by the NT.

Previously, we observed in MD simulations that differences in the sequences of the TM domains of p75, TrkA, and TrkB result in distinct signaling mechanisms through the TM helix homodimers.[56] Here, this MD simulation study shows that glycosylation also affects the ECDs of the three NT receptors in different ways and that it has roles in shielding and in modulating the receptor conformational ensemble that affect NT binding and may thereby impact NT signaling.

DATA AVAILABILITY

- Input and coordinate files have been deposited on Zenodo at https://doi.org/10.5281/zenodo.17764984.
- All other data are available from the authors upon request.

ACKNOWLEDGMENTS

The authors acknowledge provision of computing resources by the state of Baden-Württemberg through bwHPC and the German Research Foundation through grant INST 35/1134-1 FUGG. We thank Stefan Richter for technical support. This research was funded by the European Union's Horizon 2020 research and innovation program "Euroneurotrophin" under the Marie Skłodowska-Curie grant agreement no. 765704 and the Klaus Tschira Foundation.

AUTHOR CONTRIBUTIONS

Conceptualization, C.A., A.T., and R.C.W.; methodology, C.A., A.T., and R.C.W.; investigation, C.A. and A.T.; visualization, C.A. and A.T.; funding acquisition, R.C.W.; project administration, R.C.W.; supervision, R.C.W.; writing – original draft, C.A. and A.T.; writing – review and editing, C.A., A.T., and R.C.W.

DECLARATION OF INTERESTS

C.A. is an employee of AstraZeneca at the time of publication and may hold stock options. R.C.W. is a member of the Editorial Board of the *Biophysical Journal*.

SUPPLEMENTAL INFORMATION

Supporting material can be found online at https://doi.org/10.1016/j.bpj.2026.04.017.

REFERENCES

1. Varki, A., R. D. Cummings, …, P. H. ,E. Seeberger. 2022. Essentials of Glycobiology, 4th ed. Cold Spring Harbor Laboratory Press.
2. Chao, M. V. 2003. Neurotrophins and their receptors: A convergence point for many signalling pathways. *Nat. Rev. Neurosci.* 4:299–309. https://doi.org/10.1038/nrn1078.
3. Watson, F. L., M. A. Porcionatto, …, R. A. Segal. 1999. TrkA glycosylation regulates receptor localization and activity. *J. Neurobiol.* 39:323–336.
4. Settineri, C. A., I. Leung, …, A. L. Burlingame. 1992. Characterization of the Glycosylation on Recombinant Human Low-Affinity Nerve Growth Factor Receptor. *In* Techniques in Protein Chemistry III Elsevier, pp. 295–301.
5. Haniu, M., S. Montestruque, …, M. F. Rohde. 1997. Interactions between Brain-derived Neurotrophic Factor and the TRKB Receptor. *J. Biol. Chem.* 272:25296–25303. https://doi.org/10.1074/jbc.272.40.25296.
6. Yeaman, C., A. H. Le Gall, …, E. Rodriguez-Boulan. 1997. The O-glycosylated Stalk Domain Is Required for Apical Sorting of Neurotrophin Receptors in Polarized MDCK Cells. *J. Cell Biol.* 139:929–940. https://doi.org/10.1083/jcb.139.4.929.
7. Monlauzeur, L., L. Breuza, and A. Le Bivic. 1998. Putative O-Glycosylation Sites and a Membrane Anchor Are Necessary for Apical Delivery of the Human Neurotrophin Receptor in Caco-2 Cells. *J. Biol. Chem.* 273:30263–30270. https://doi.org/10.1074/jbc.273.46.30263.
8. Breuza, L., M. Garcia, …, A. Le Bivic. 2002. Role of the Membrane-Proximal O-glycosylation Site in Sorting of the Human Receptor for Neurotrophins to the Apical Membrane of MDCK Cells. *Exp. Cell Res.* 273:178–186. https://doi.org/10.1006/excr.2001.5442.
9. Haniu, M., J. Talvenheimo, …, M. F. Rohde. 1995. Extracellular Domain of Neurotrophin Receptor trkB: Disulfide Structure, N-Glycosylation Sites, and Ligand Binding. *Arch. Biochem. Biophys.* 322:256–264. https://doi.org/10.1006/abbi.1995.1460.
10. Baldwin, A. N., and E. M. Shooter. 1995. Zone Mapping of the Binding Domain of the Rat Low Affinity Nerve Growth Factor Receptor by the Introduction of Novel N-Glycosylation Sites. *J. Biol. Chem.* 270:4594–4602. https://doi.org/10.1074/jbc.270.9.4594.

11. Wehrman, T., X. He, …, K. C. Garcia. 2007. Structural and Mechanistic Insights into Nerve Growth Factor Interactions with the TrkA and p75 Receptors. *Neuron.* 53:25–38. https://doi.org/10.1016/j.neuron.2006.09.034.

12. Grob, P. M., A. H. Ross, …, M. Bothwell. 1985. Characterization of the human melanoma nerve growth factor receptor. *J. Biol. Chem.* 260:8044–8049.

13. Trabjerg, E., F. Kartberg, …, K. D. Rand. 2017. Conformational characterization of nerve growth factor-β reveals that its regulatory pro-part domain stabilizes three loop regions in its mature part. *J. Biol. Chem.* 292:16665–16676. https://doi.org/10.1074/jbc.M117.803320.

14. Benicky, J., M. Sanda, …, R. Goldman. 2019. N-Glycosylation is required for secretion of the precursor to brain-derived neurotrophic factor (proBDNF) carrying sulfated LacdiNAc structures. *J. Biol. Chem.* 294:16816–16830. https://doi.org/10.1074/jbc.RA119.009989.

15. Chapman, B. S., M. R. Eckart, …, G. R. Lapointe. 1996. O-Linked Oligosaccharide on the 75-kDa Neurotrophin Receptor. *J. Neurochem.* 66:1707–1716. https://doi.org/10.1046/j.1471-4159.1996.66041707.x.

16. Davis, S. J., and M. Crispin. 2010. Solutions to the Glycosylation Problem for Low- and High-Throughput Structural Glycoproteomics. *In* Functional and Structural Proteomics of Glycoproteins Springer, pp. 127–158.

17. Kozak, S., Y. Bloch, …, R. Meijers. 2020. Homogeneously N -glycosylated proteins derived from the GlycoDelete HEK293 cell line enable diffraction-quality crystallogenesis. *Acta Crystallogr. D Struct. Biol.* 76:1244–1255. https://doi.org/10.1107/S2059798320013753.

18. Casalino, L., Z. Gaieb, …, R. E. Amaro. 2020. Beyond Shielding: The Roles of Glycans in the SARS-CoV-2 Spike Protein. *ACS Cent. Sci.* 6:1722–1734. https://doi.org/10.1021/acscentsci.0c01056.

19. Harbison, A. M., C. A. Fogarty, …, E. Fadda. 2022. Fine-tuning the spike: role of the nature and topology of the glycan shield in the structure and dynamics of the SARS-CoV-2 S. *Chem. Sci.* 13:386–395. https://doi.org/10.1039/D1SC04832E.

20. Vuorio, J., J. Škerlová, …, H. Martinez-Seara. 2021. N-Glycosylation can selectively block or foster different receptor–ligand binding modes. *Sci. Rep.* 11:5239. https://doi.org/10.1038/s41598-021-84569-z.

21. Mehdipour, A. R., and G. Hummer. 2021. Dual nature of human ACE2 glycosylation in binding to SARS-CoV-2 spike. *Proc. Natl. Acad. Sci.* 118:e2100425118. https://doi.org/10.1073/pnas.2100425118.

22. Gong, Y., P. Cao, …, T. Jiang. 2008. Crystal structure of the neurotrophin-3 and p75NTR symmetrical complex. *Nature.* 454:789–793. https://doi.org/10.1038/nature07089.

23. 2020. Schrödinger Release 2020-2. Maestro, Schrödinger, LLC.

24. Šali, A., and T. L. Blundell. 1993. Comparative Protein Modelling by Satisfaction of Spatial Restraints. *J. Mol. Biol.* 234:779–815. https://doi.org/10.1006/jmbi.1993.1626.

25. Olsson, M. H. M., C. R. Søndergaard, …, J. H. Jensen. 2011. PROPKA3: Consistent Treatment of Internal and Surface Residues in Empirical p K a Predictions. *J. Chem. Theory Comput.* 7:525–537. https://doi.org/10.1021/ct100578z.

26. Jurrus, E., D. Engel, …, N. A. Baker. 2018. Improvements to the APBS biomolecular solvation software suite. *Protein Sci.* 27:112–128. https://doi.org/10.1002/pro.3280.

27. Dobson, C. L., P. W. A. Devine, …, D. C. Lowe. 2016. Engineering the surface properties of a human monoclonal antibody prevents self-association and rapid clearance in vivo. *Sci. Rep.* 6:38644. https://doi.org/10.1038/srep38644.

28. Banfield, M. J., R. L. Naylor, …, R. L. Brady. 2001. Specificity in Trk Receptor:Neurotrophin Interactions. *Structure.* 9:1191–1199. https://doi.org/10.1016/S0969-2126(01)00681-5.

29. Robinson, R. C., C. Radziejewski, …, E. Y. Jones. 1999. The structures of the neurotrophin 4 homodimer and the brain-derived neurotrophic factor/neurotrophin 4 heterodimer reveal a common Trk-binding site. *Protein Sci.* 8:2589–2597. https://doi.org/10.1110/ps.8.12.2589.

30. Fleming, J., P. Magana, …, S. Velankar. 2025. AlphaFold Protein Structure Database and 3D-Beacons: New Data and Capabilities. *J. Mol. Biol.* 437:168967. https://doi.org/10.1016/j.jmb.2025.168967.

31. Jumper, J., R. Evans, …, D. Hassabis. 2021. Highly accurate protein structure prediction with AlphaFold. *Nature.* 596:583–589. https://doi.org/10.1038/s41586-021-03819-2.

32. Jullien, J., V. Guili, …, B. B. Rudkin. 2002. Molecular Kinetics of Nerve Growth Factor Receptor Trafficking and Activation. *J. Biol. Chem.* 277:38700–38708. https://doi.org/10.1074/jbc.M202348200.

33. Woronowicz, A., S. R. Amith, …, M. R. Szewczuk. 2007. Dependence of neurotrophic factor activation of Trk tyrosine kinase receptors on cellular sialidase. *Glycobiology.* 17:10–24. https://doi.org/10.1093/glycob/cwl049.

34. Park, S.-J., J. Lee, …, W. Im. 2019. CHARMM-GUI Glycan Modeler for modeling and simulation of carbohydrates and glycoconjugates. *Glycobiology.* 29:320–331. https://doi.org/10.1093/glycob/cwz003.

35. Huang, J., S. Rauscher, …, A. D. MacKerell. 2017. CHARMM36m: an improved force field for folded and intrinsically disordered proteins. *Nat. Methods.* 14:71–73. https://doi.org/10.1038/nmeth.4067.

36. Abraham, M. J., T. Murtola, …, E. Lindahl. 2015. GROMACS: High performance molecular simulations through multi-level parallelism from laptops to supercomputers. *SoftwareX.* 1–2:19–25. https://doi.org/10.1016/j.softx.2015.06.001.

37. Nosé, S. 1984. A molecular dynamics method for simulations in the canonical ensemble. *Mol. Phys.* 52:255–268. https://doi.org/10.1080/00268978400101201.

38. Hoover, W. G. 1985. Canonical dynamics: Equilibrium phase-space distributions. *Phys. Rev. A.* 31:1695–1697. https://doi.org/10.1103/PhysRevA.31.1695.

39. Parrinello, M., and A. Rahman. 1981. Polymorphic transitions in single crystals: A new molecular dynamics method. *J. Appl. Phys.* 52:7182–7190. https://doi.org/10.1063/1.328693.

40. Nosé, S., and M. L. Klein. 1983. Constant pressure molecular dynamics for molecular systems. *Mol. Phys.* 50:1055–1076. https://doi.org/10.1080/00268978300102851.

41. Darden, T., D. York, and L. Pedersen. 1993. Particle mesh Ewald: An N ·log(N) method for Ewald sums in large systems. *J. Chem. Phys.* 98:10089–10092. https://doi.org/10.1063/1.464397.

42. Essmann, U., L. Perera, …, L. G. Pedersen. 1995. A smooth particle mesh Ewald method. *J. Chem. Phys.* 103:8577–8593. https://doi.org/10.1063/1.470117.

43. Hess, B., H. Bekker, …, J. G. E. M. Fraaije. 1997. LINCS: A linear constraint solver for molecular simulations. *J. Comput. Chem.* 18:1463–1472.

44. Humphrey, W., A. Dalke, and K. Schulten. 1996. VMD: Visual molecular dynamics. *J. Mol. Graph.* 14:33. https://doi.org/10.1016/0263-7855(96)00018-5.

45. Gowers, R., M. Linke, …, O. Beckstein. 2016. MDAnalysis: A Python Package for the Rapid Analysis of Molecular Dynamics Simulations, pp. 98–105.

46. Michaud-Agrawal, N., E. J. Denning, …, O. Beckstein. 2011. MDAnalysis: A toolkit for the analysis of molecular dynamics simulations. *J. Comput. Chem.* 32:2319–2327. https://doi.org/10.1002/jcc.21787.

47. Hunter, J. D. 2007. Matplotlib: A 2D Graphics Environment. *Comput. Sci. Eng.* 9:90–95. https://doi.org/10.1109/MCSE.2007.55.

48. Waskom, M. 2021. Seaborn: statistical data visualization. *J. Open Source Softw.* 6:3021. https://doi.org/10.21105/joss.03021.

49. Harris, C. R., K. J. Millman, …, T. E. Oliphant. 2020. Array programming with NumPy. *Nature.* 585:357–362. https://doi.org/10.1038/s41586-020-2649-2.

50. 2020. The Pandas Development Team. pandas-dev/pandas: Pandas, Zenodo.

51. McKinney, W. 2010. Data Structures for Statistical Computing in Python. *In* Proceedings of the 9th Python in Science Conference. S. van der Walt and J. Millman, eds, pp. 56–61.

52. Rodríguez-Tébar, A., G. Dechant, …, Y. A. Barde. 1992. Binding of neurotrophin-3 to its neuronal receptors and interactions with nerve growth factor and brain-derived neurotrophic factor. *EMBO J.* 11:917–922. https://doi.org/10.1002/j.1460-2075.1992.tb05130.x.

53. Bothwell, M. 1995. Functional interactions of neurotrophins and neurotrophin receptors. *Annu. Rev. Neurosci.* 18:223–253. https://doi.org/10.1146/annurev.ne.18.030195.001255.

54. Forsyth, P. A., N. Krishna, …, R. S. Kenchappa. 2014. p75 Neurotrophin Receptor Cleavage by α- and γ-Secretases Is Required for Neurotrophin-mediated Proliferation of Brain Tumor-initiating Cells. *J. Biol. Chem.* 289:8067–8085. https://doi.org/10.1074/jbc.M113.513762.

55. Pietropaolo, A., A. Magrì, …, E. Rizzarelli. 2018. Binding of Zn(II) to Tropomyosin Receptor Kinase A in Complex with Its Cognate Nerve Growth Factor: Insights from Molecular Simulation and in Vitro Essays. *ACS Chem. Neurosci.* 9:1095–1103. https://doi.org/10.1021/acschemneuro.7b00470.

56. Athanasiou, C., A. C. Cabezudo, …, R. C. Wade. 2025. Simulation of neurotrophin receptor transmembrane helix interactions reveals active states and distinct signaling mechanisms. *JACS Au.* 5:2186–2200. https://doi.org/10.1021/jacsau.5c00174.

Biophysical Journal, Volume *125*

Supplemental information

Glycosylation-modulated conformational diversity in neurotrophin receptors

Alexandros Tsengenes, Christina Athanasiou, and Rebecca C. Wade

Supporting Material

Glycosylation-Modulated Conformational Diversity in Neurotrophin Receptors

Alexandros Tsengenes[1,2,3]†, Christina Athanasiou[1,2,3]†, Rebecca C. Wade[1,2,4,5]

[1] Molecular and Cellular Modeling Group, Heidelberg Institute for Theoretical Studies (HITS), 69118 Heidelberg, Germany.

[2] Faculty of Biosciences, Heidelberg University, 69120 Heidelberg, Germany.

[3] Heidelberg Biosciences International Graduate School, Heidelberg University, 69120 Heidelberg, Germany.

[4] Center for Molecular Biology of Heidelberg University (ZMBH), DKFZ-ZMBH Alliance, 69120 Heidelberg, Germany.

[5] Interdisciplinary Center for Scientific Computing (IWR), Heidelberg University, 69120 Heidelberg, Germany.

Table S1: Summary of simulated systems with or without glycans.

System No	Protein system	Glycosylation state	Replicas	Time/Replica (ns)
1	p75 dimer – NT3 dimer	+	3	500
2		-	3	500
3	p75 monomer – NT3 dimer	+	3	500
4		-	3	500
5	p75 monomer	+	3	500
6		-	3	500
7	TrkA dimer – NGF dimer	+	6	500
8		-	6	500
9	TrkA monomer – NGF dimer	+	6	500
10		-	6	500
11	TrkA monomer	+	3	500
12		-	3	500
13	TrkB dimer – NT-4/5 dimer	+	6	500
14		-	6	500
15	TrkB monomer – NT-4/5 dimer	+	6	500
16		-	6	500
17	TrkB monomer	+	6	500
18		-	6	500

Table S2: N-glycosylation positions on TrkA and TrkB receptors. For TrkB, the exact glycosylation positions are known experimentally.[1,2] For TrkA, the glycosylation positions that are known from the PDB ID: 2IFG[3] crystal structure are shown with normal font, while the ones that were added based on comparison with TrkB positions are shown in bold letters.

TrkA	TrkB
**Asn 67**	Asn 67
Asn 95	Asn 95
Asn 121	Asn 121
Asn 188	Asn 178
**Asn 202**	Asn 205
-	Asn 241
**Asn 253**	Asn 254
Asn 262	-
Asn 281	Asn 280
-	Asn 338
Asn 358	-
**Asn 401**	Asn 412

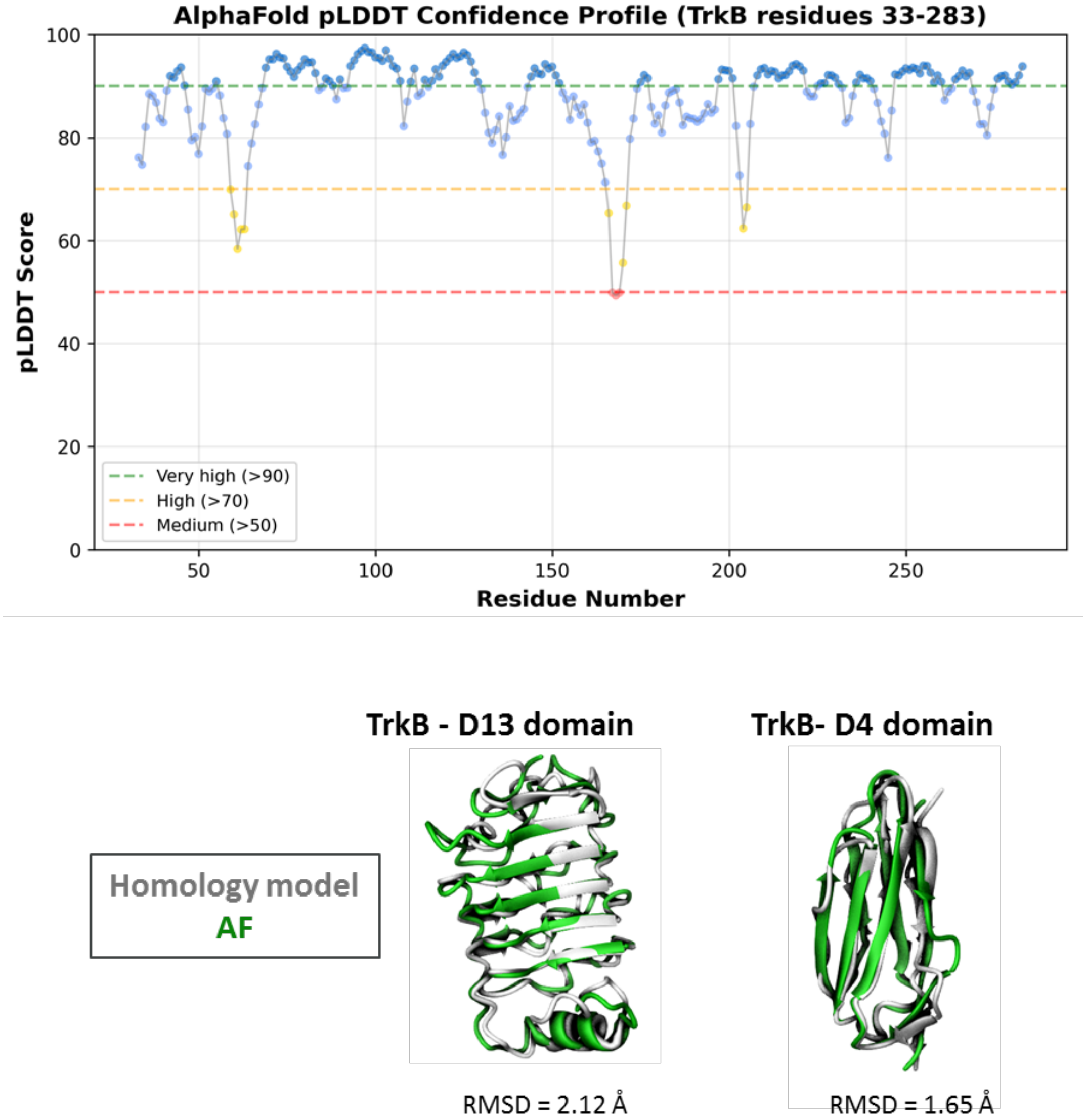

Figure S1: AlphaFold (AF) pLDDT score for the D1-D4 domains of TrkB (AF-Q16620-2-F1-v6 from the AlphaFold Protein Structure database (https://alphafold.ebi.ac.uk/)) and structural alignments of the D13 and D4 domains from the TrkB homology model used in the molecular dynamics simulations with the same domains from the AF model. Backbone RMSD values for the alignments are indicated below the figures.

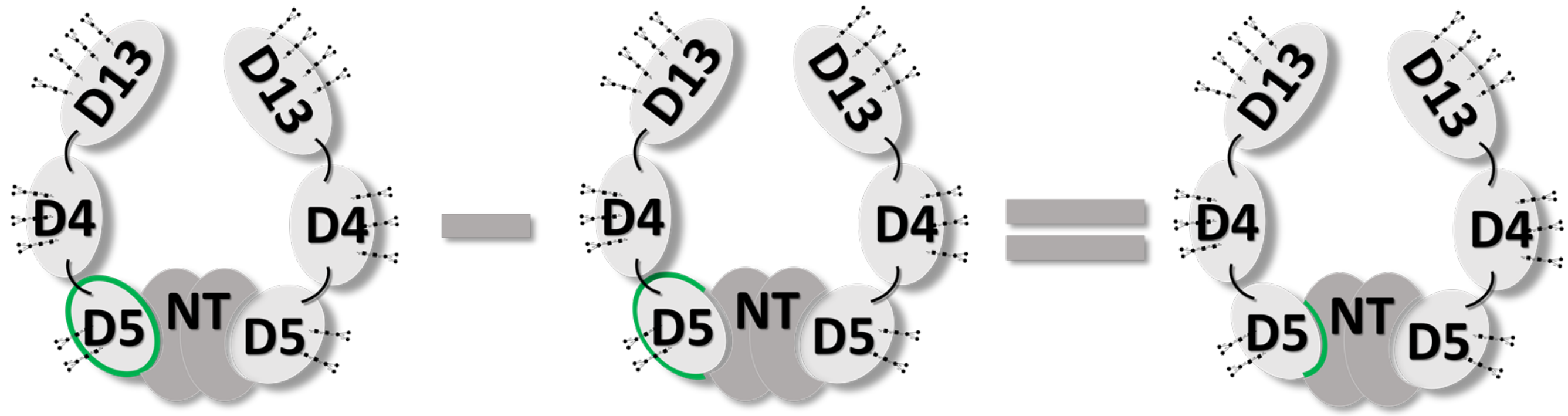

Figure S2: Schematic representation of the solvent accessible surface area (SASA) calculations that were performed to estimate the contact area of the EC domains of the receptors and the NTs. Here, the contact area between the D5 domain of the Trks and the NTs is shown as an example. For this purpose, the SASA of the D5 domain was calculated without and with the NT present and the difference of these two values yielded the contact area of D5-NT.

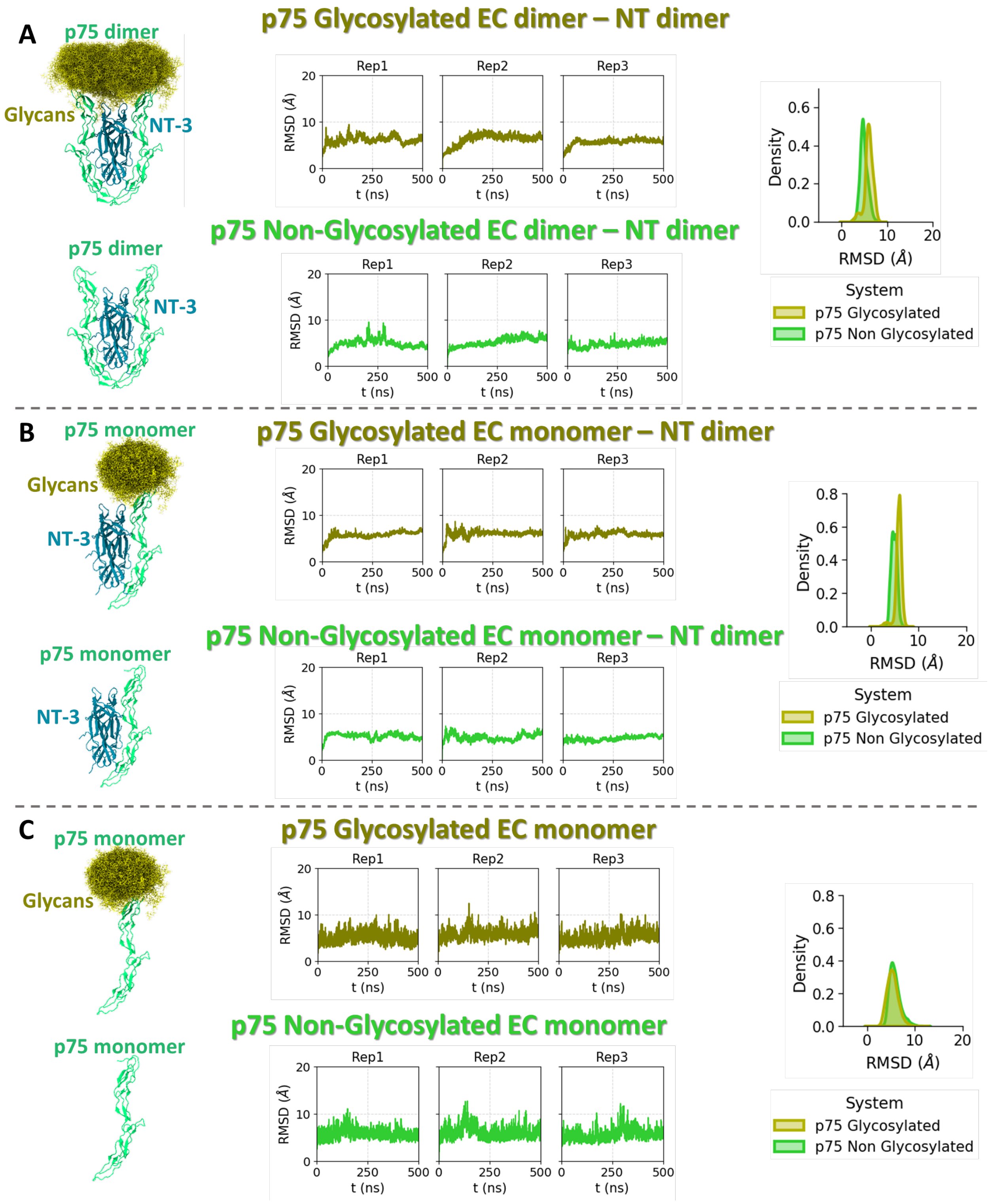

Figure S3: Summary of RMSD calculation results for the (A) p75-EC as a homodimer in complex to NT-3, (B) as a monomer in complex with NT-3 and (C) as a monomer alone, with and without glycans. The RMSD values are presented as an evolution with time (middle plots) and as probability densities for all the replicas together in the glycosylated and non-glycosylated systems (right plots).

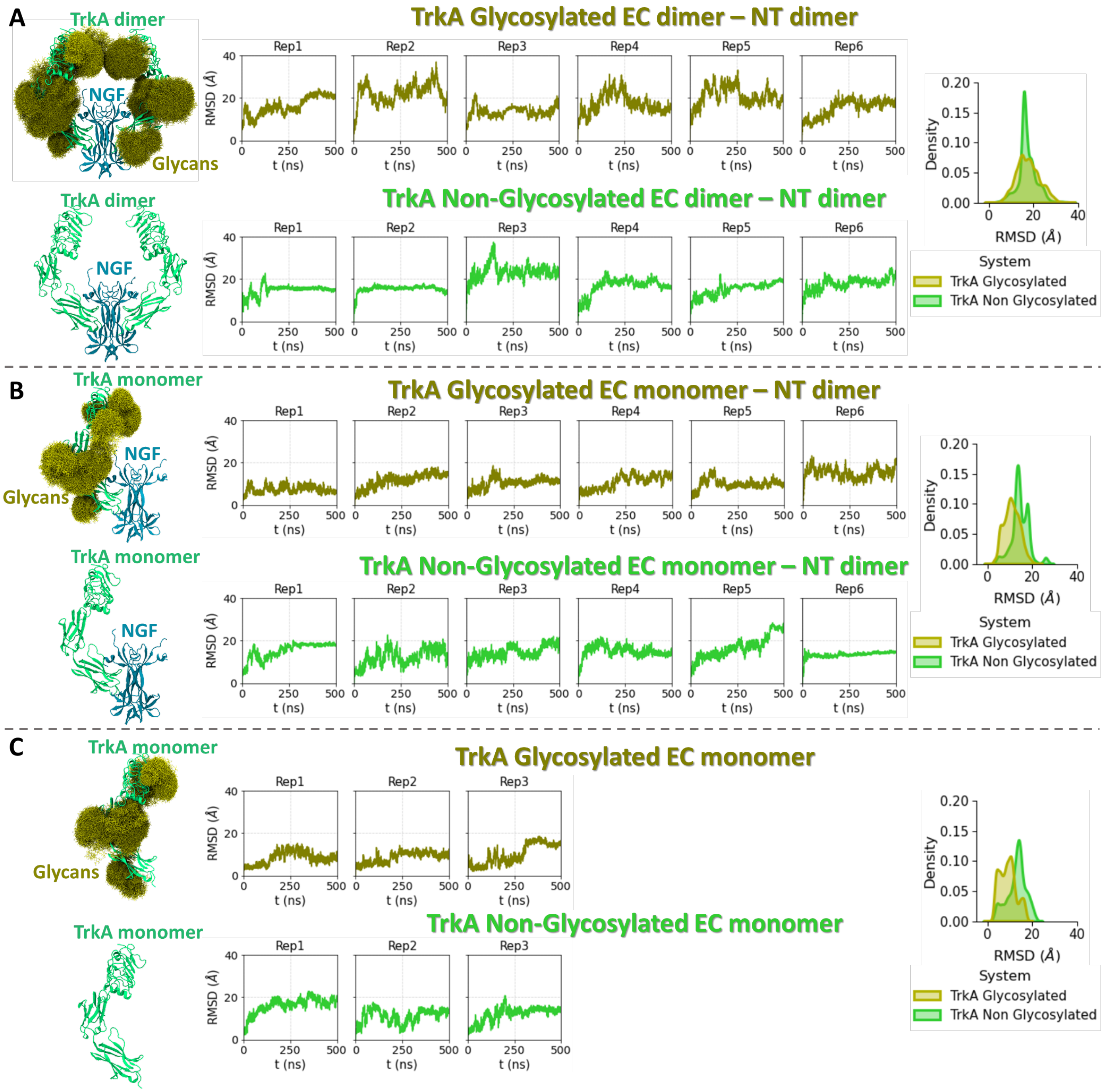

Figure S4: RMSD calculations of the glycosylated and non-glycosylated TrkA systems. (A), (B) and (C) show the RMSD for the TrkA-EC homodimer with the NGF homodimer bound, the TrkA-EC monomer with the NGF homodimer bound, the TrkA-EC monomer systems, respectively, with and without glycans. For each system the time-dependence of the RMSD of the protein backbone compared to the first frame (which corresponds to the crystal structure) is shown for each replica, as well as the distributions of RMSD values across all replicas and all frames of the production runs for the glycosylated and non-glycosylated systems (right plots in A, B and C).

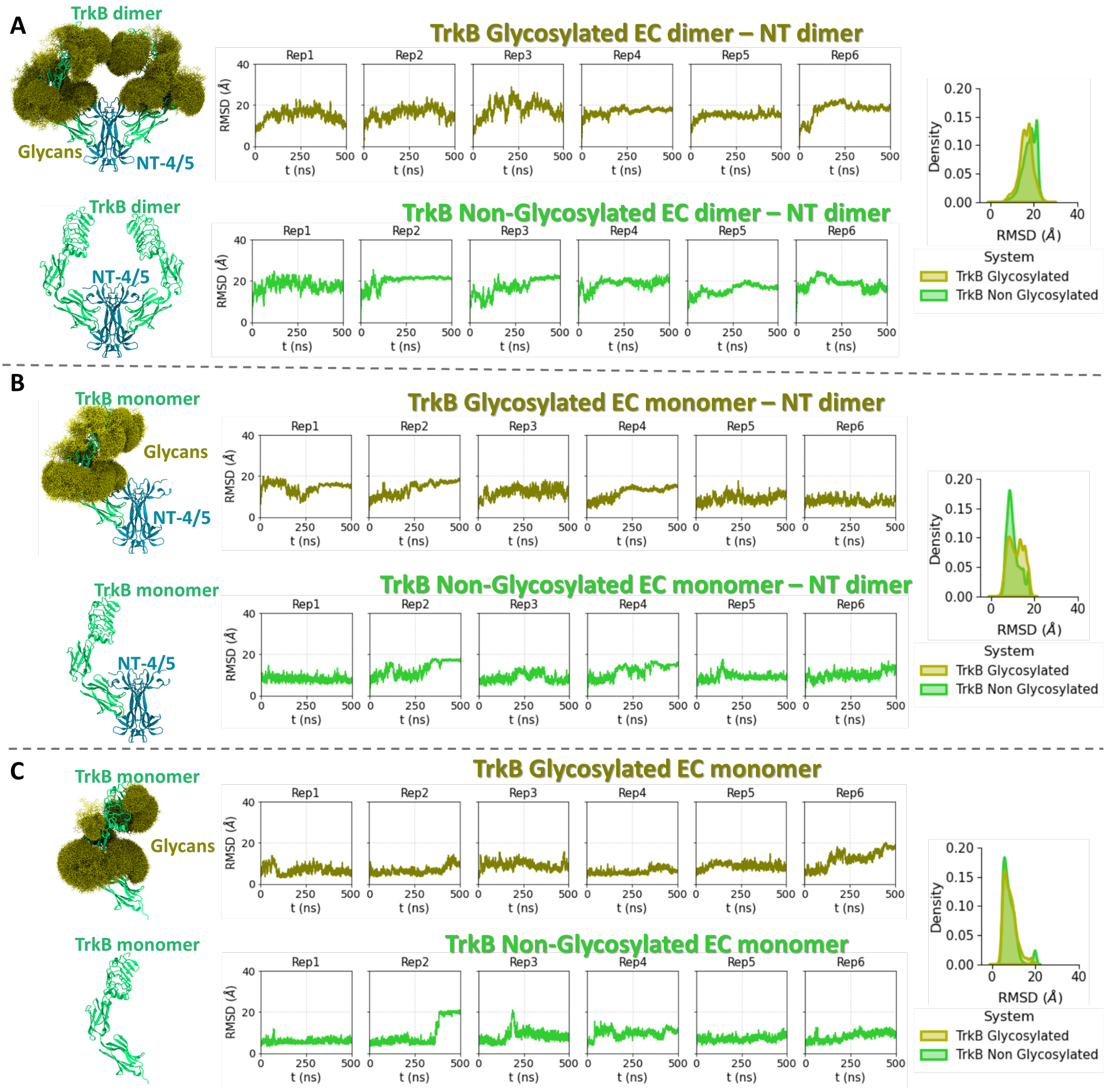

Figure S5: RMSD calculations of the glycosylated and non-glycosylated TrkB systems. (A), (B) and (C) show the RMSD for the TrkB-EC homodimer with the NT-4/5 homodimer bound, the TrkB-EC monomer with the NT-4/5 homodimer bound, the TrkB-EC monomer systems, respectively, with and without glycans. For each system the time-dependence of the RMSD of the protein backbone compared to the first frame (which corresponds to the crystal structure) is shown for each replica, as well as the distributions of RMSD values across all replicas and all frames of the production runs for the glycosylated and non-glycosylated systems (right plots in A, B and C).

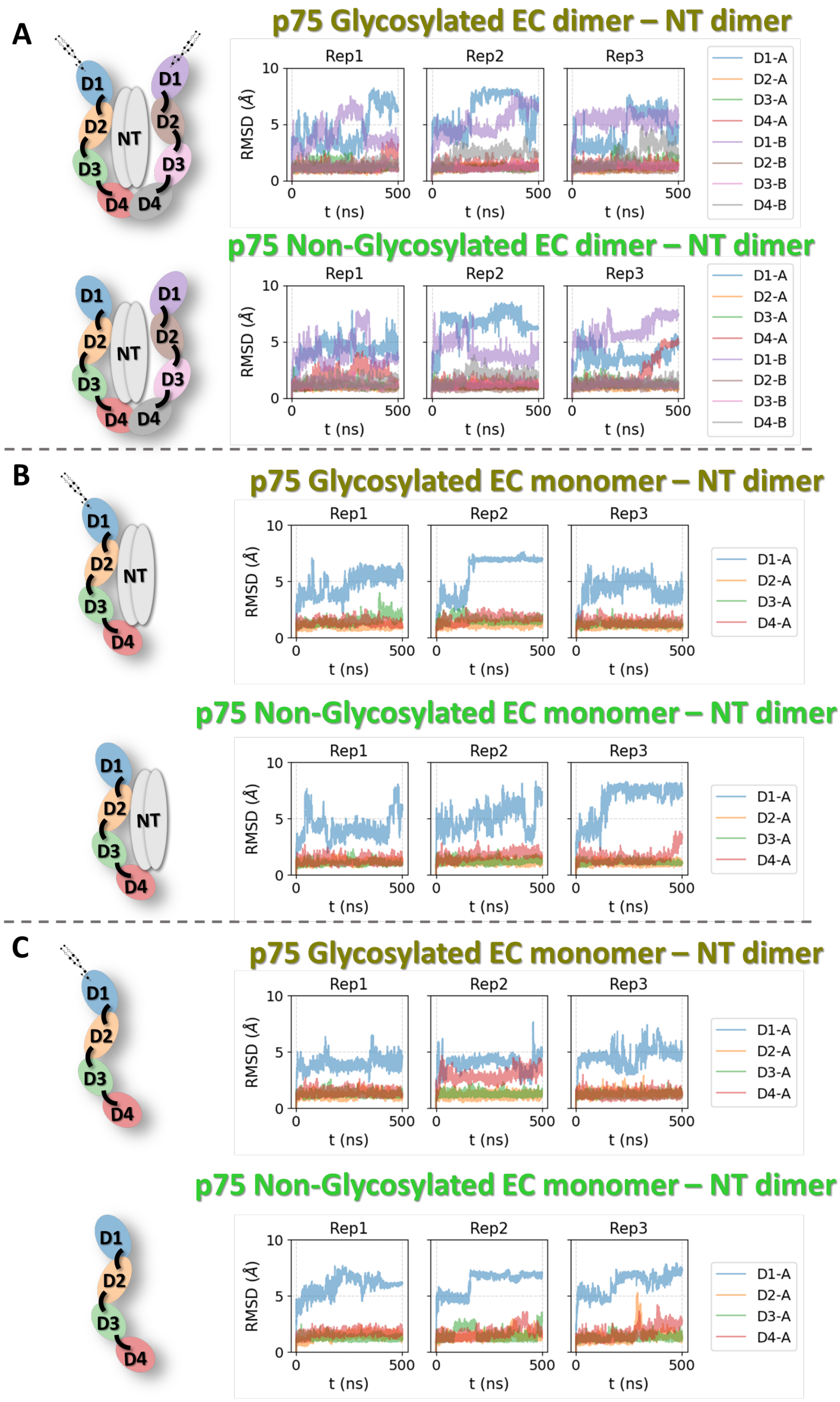

Figure S6: Summary of sub-domain RMSD calculation results for the (A) p75-EC as a homodimer in complex to NT-3, (B) as a monomer in complex with NT-3 and (C) as a monomer alone, with and without glycans. The RMSD values are presented as an evolution with time for each of the domains, after alignment to each domain.

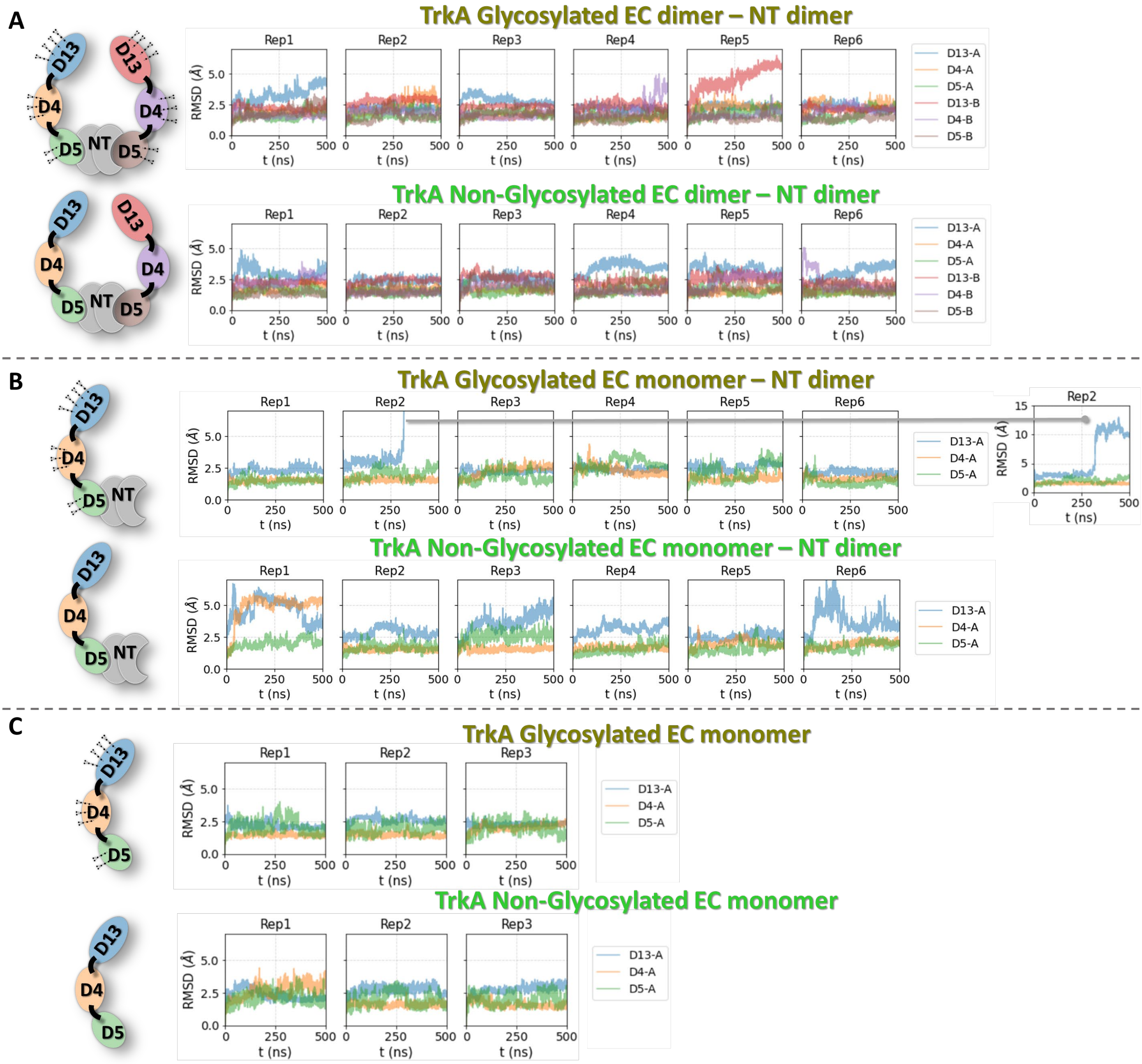

Figure S7: RMSD calculations for each domain of the glycosylated or not TrkA systems. (A), (B) and (C) show the internal RMSD for each domain the TrkA-EC homodimer with the NGF homodimer bound, the TrkA-EC monomer with the NGF homodimer bound, the TrkA-EC monomer systems, respectively, with and without glycans. For each system, the time-dependent RMSD of the domain backbone compared to the first frame is shown for each replica. Before the calculation of the internal RMSD of each domain, I performed alignment of the respective domain at each simulation snapshot. Schematic representation of each system, with color-coding of the domains is shown at the left. The first and second subunits are indicated with A and B letters in the legends.

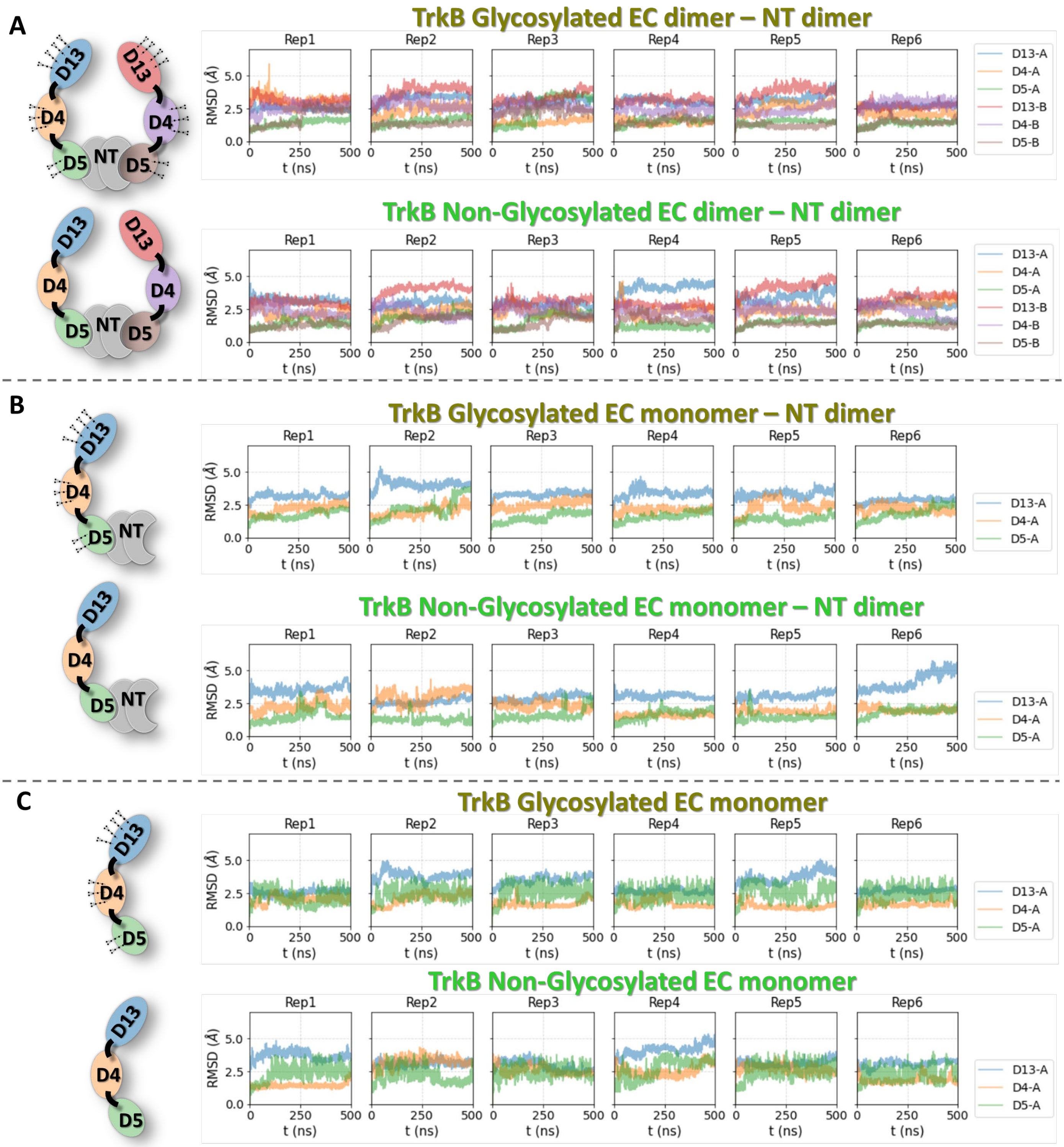

Figure S8: RMSD calculations for each domain of the glycosylated or not TrkB systems. (A), (B) and (C) show the internal RMSD for each domain the TrkB-EC homodimer with the NT-4/5 homodimer bound, the TrkB-EC monomer with the NT-4/5 homodimer bound, the TrkB-EC monomer systems, respectively, with and without glycans. For each system, the time-dependent RMSD of the domain backbone compared to the first frame is shown for each replica. Before the calculation of the internal RMSD of each domain, I performed alignment in the respective domain at each simulation snapshot. Schematic representation of each system, with color-coding of the domains is shown at the left. The first and second monomers are indicated with A and B letters in the legends.

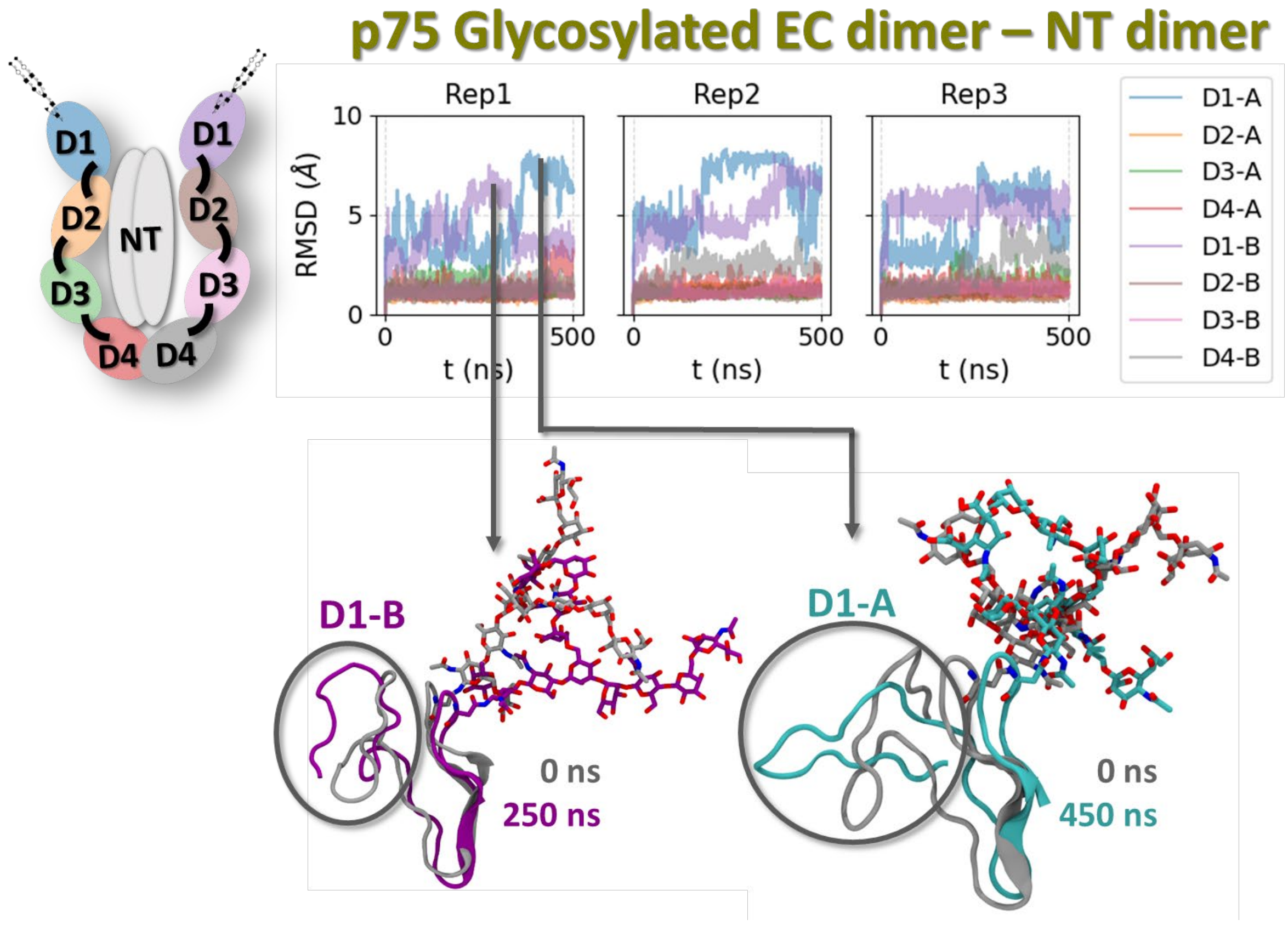

Figure S9: Closer examination of the p75-D1 domain conformations with the glycan at Asn 60, which is covalently attached to the D1 domain. Two example representations of high RMSD values are shown with D1-glycan aligned to the input structure from X-ray. The high RMSD conformations are shown in purple and cyan for the two D1 monomers and the initial structures in grey. The D1 domain is shown in cartoon representation, while the glycan is shown in stick representation.

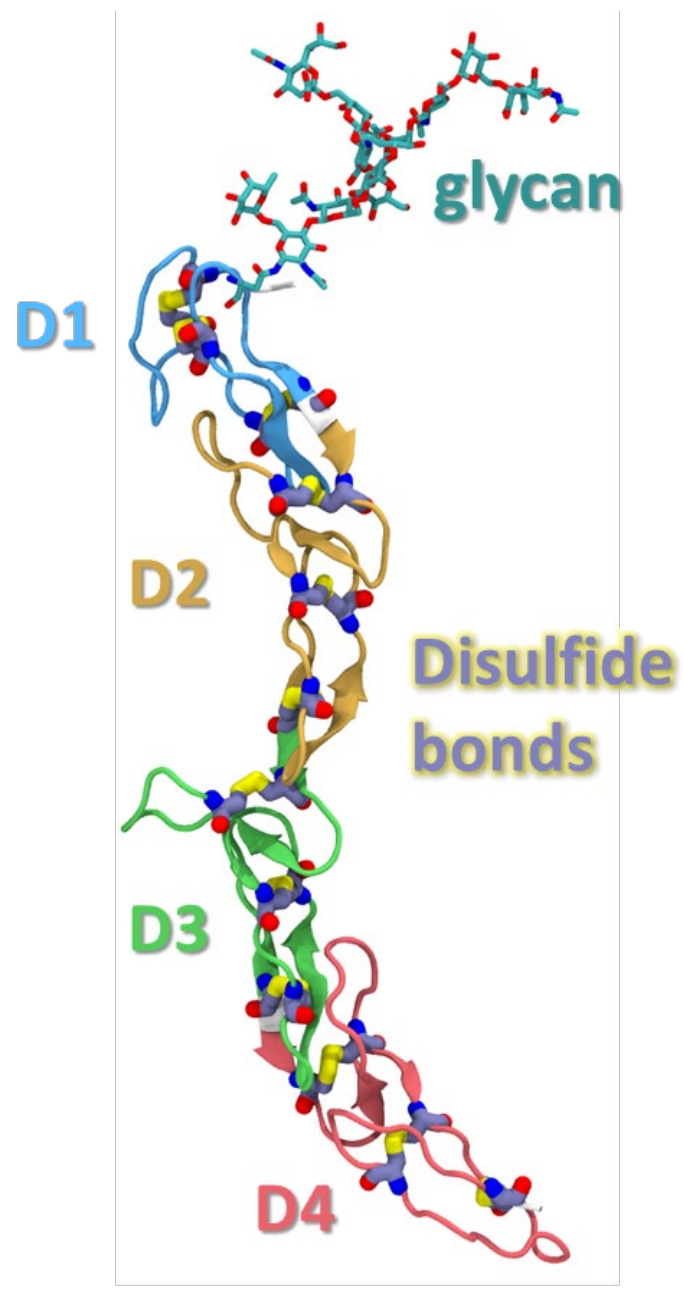

Figure S10: Disulfide bond ladder in the structure of p75-EC (from PDB ID: 3BUK). The D1-D4 domains are shown in cartoon representation. The disulfide bonds are shown in licorice representation.

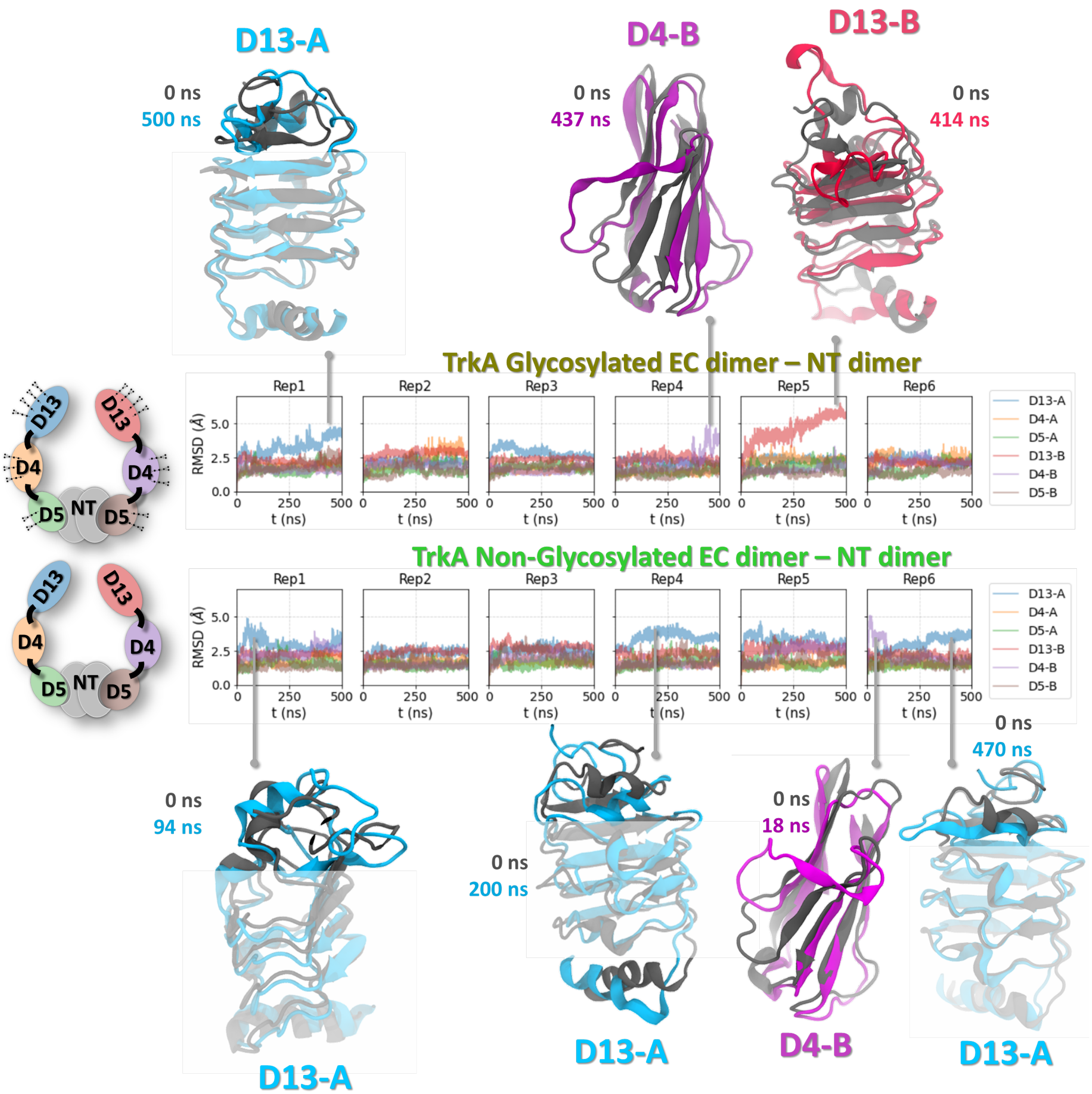

Figure S11: RMSD calculations for each domain of the glycosylated and not TrkA-EC homodimer bound to NGF system. For each system, the time-dependent RMSD of the domain backbone compared to the first frame is shown for each replica. Before the calculation of the internal RMSD of each domain, I performed alignment in the respective domain at each simulation snapshot. Schematic representation of each system, with color-coding of the domains is shown at the left. In cases of high RMSD values, the conformations of the domains are shown in cartoon representation aligned to the first frame.

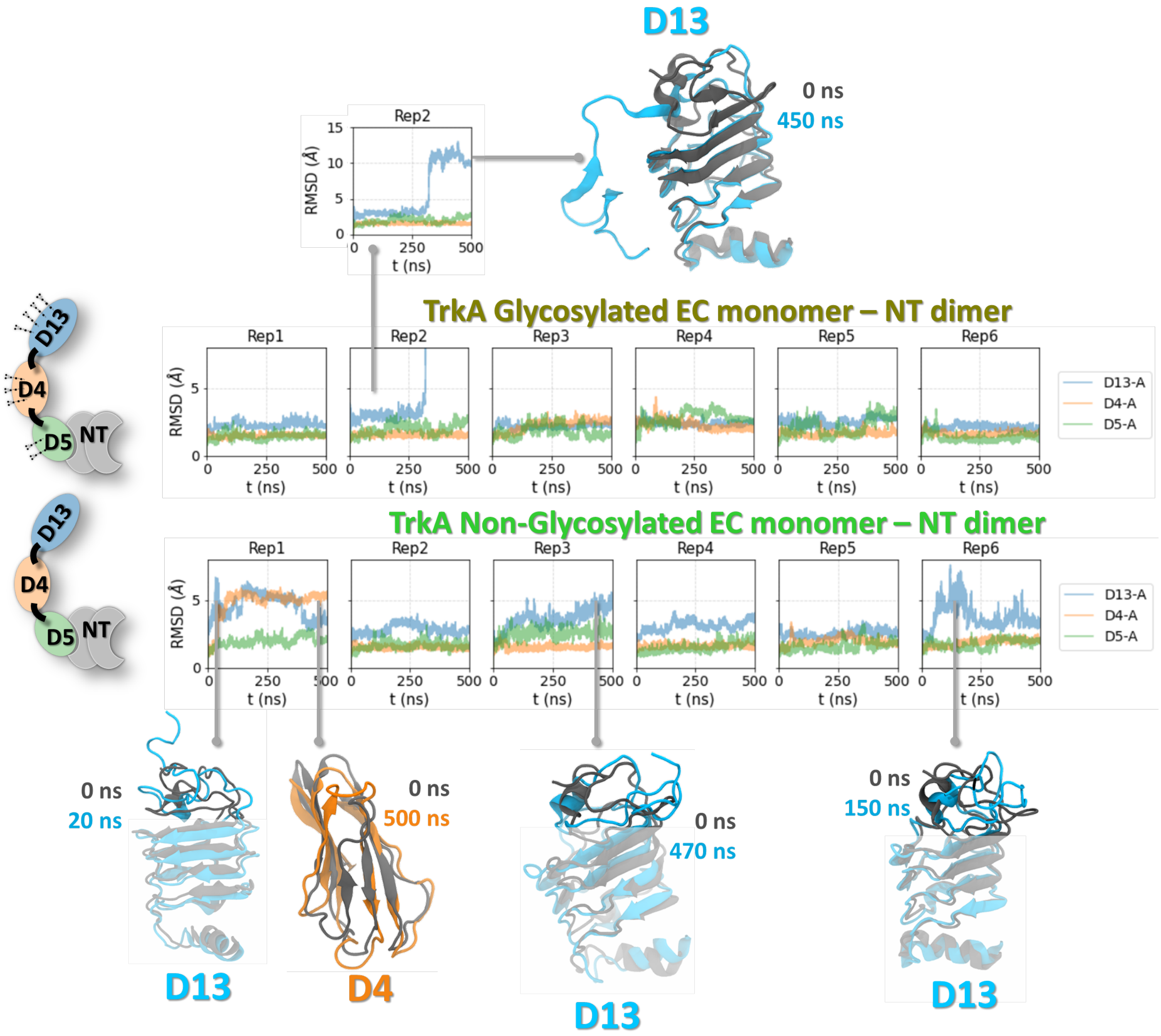

Figure S12: RMSD calculations for each domain of the glycosylated and not TrkA-EC monomer bound to NGF system. For each system, the time-dependent RMSD of the domain backbone compared to the first frame is shown for each replica. Before the calculation of the internal RMSD of each domain, I performed alignment in the respective domain at each simulation snapshot. Schematic representation of each system, with color-coding of the domains is shown at the left. In cases of high RMSD values, the conformations of the domains are shown in cartoon representation aligned to the first frame.

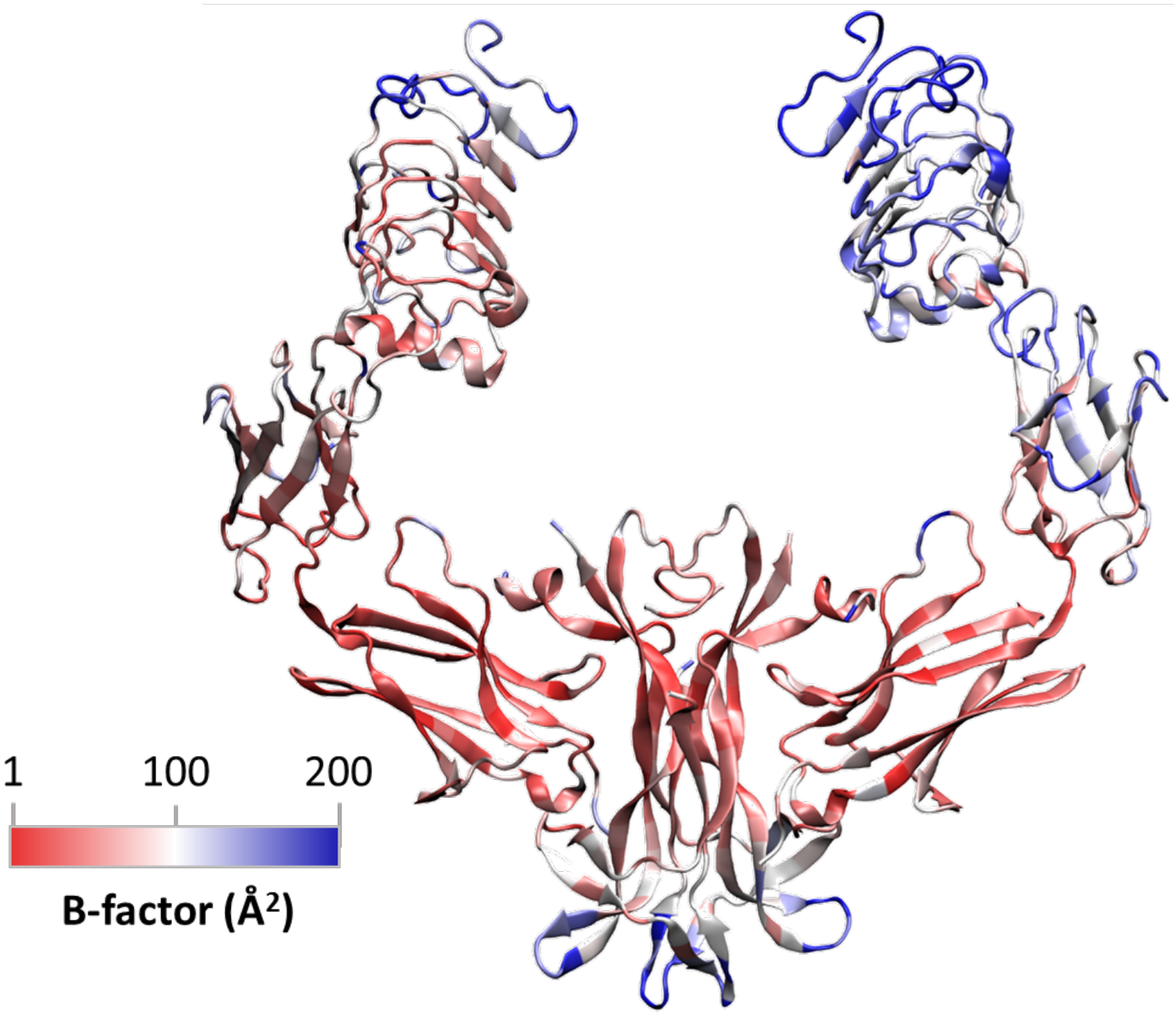

Figure S13: B-factors of the TrkA-EC bound to NGF from the crystal structure (PDB ID: 2IFG).[3]

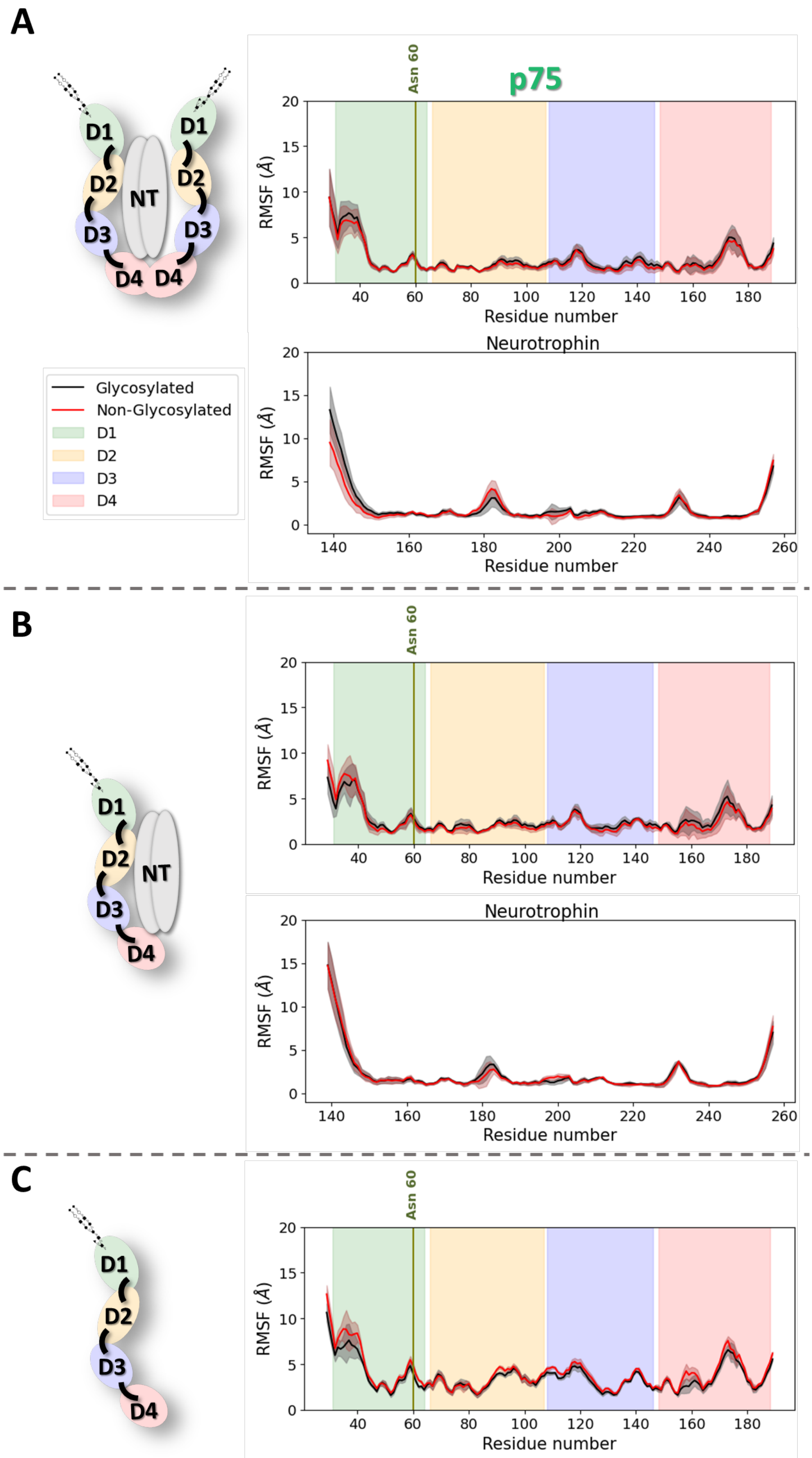

Figure S14: Summary of RMSF calculation results for the (A) p75-EC as a homodimer in complex to NT-3, (B) as a monomer in complex with NT-3 and (C) as a monomer alone, with (black) and without (red) glycans. The RMSF for each residue was calculated as an average from the three replica simulations and the standard deviation at each point is shown with shadowed areas. The different domains of p75-EC are color-coded and the glycosylated Asn 60 is indicated with a vertical line.

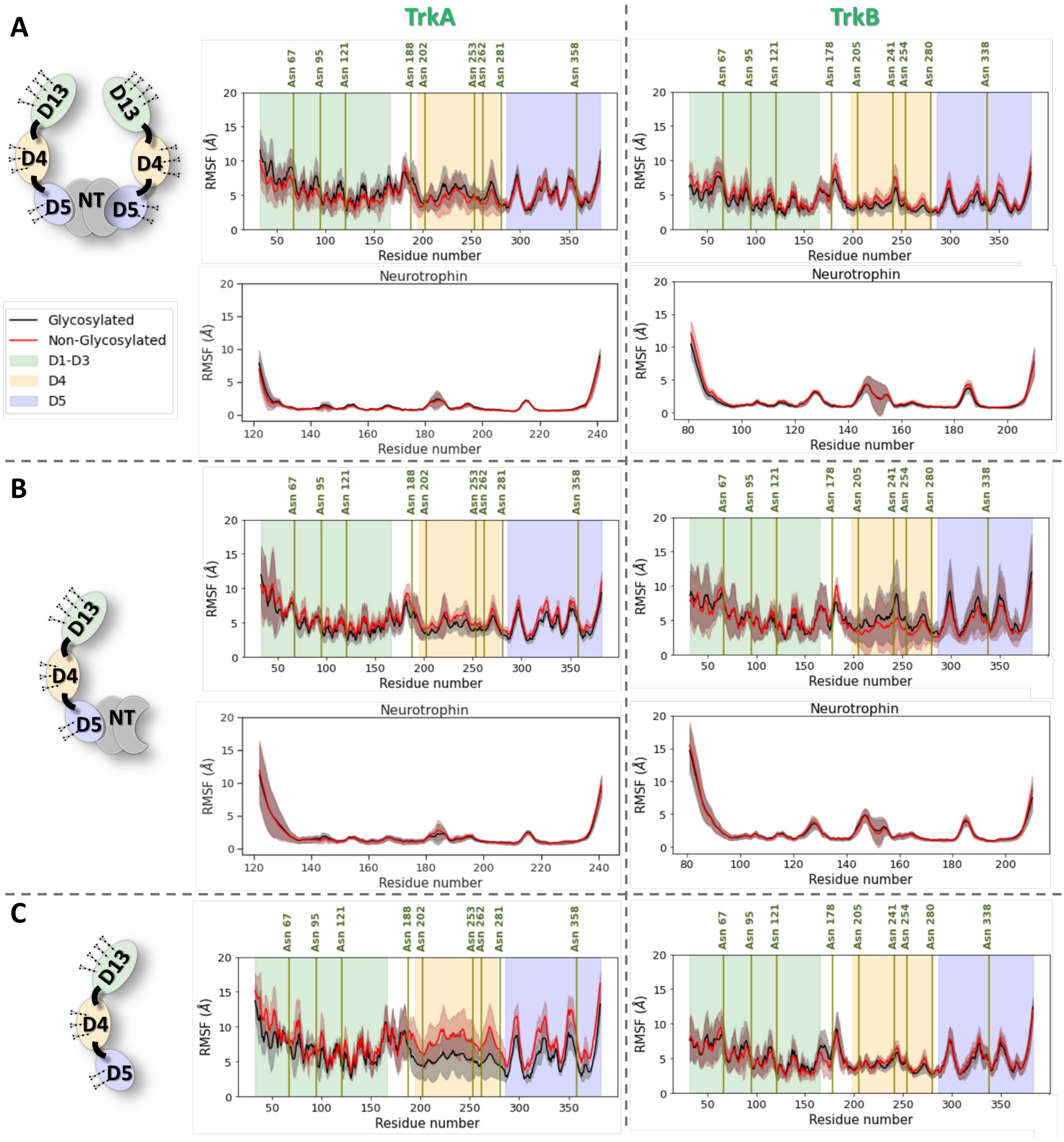

Figure S15: RMSF calculations of all the glycosylated or not TrkA and TrkB systems. (A), (B) and (C) show the average RMSF values from all replica simulations for each receptor and neurotrophin residue of the TrkA/B-EC homodimer with the NGF/ NT-4/5 homodimer bound, the TrkA/B-EC monomer with the NGF/ NT-4/5 homodimer bound, the TrkA/B-EC monomer systems, respectively, with or without glycans. The average RMSF values from all replica simulations are shown for each residue in black and red lines for the glycosylated and non-glycosylated systems, respectively. The standard deviation for each residue is shown with shadowed areas. The residue positions of glycosylated asparagines are shown with vertical lines. Before the RMSF calculation, I aligned the systems to an average structure from the simulations.

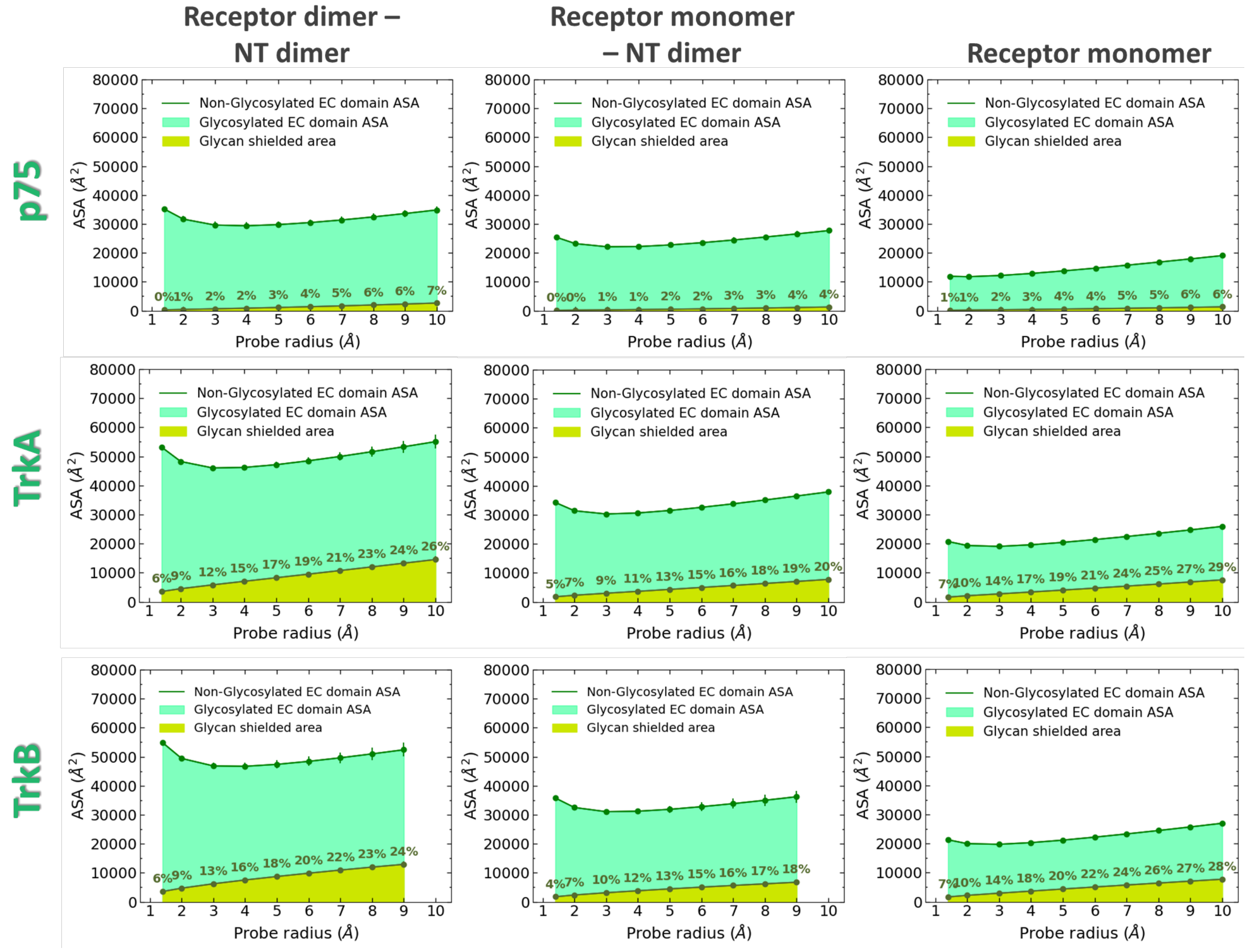

Figure S16: Accessible surface area (ASA) of the p75, TrkA and TrkB systems and the area shielded by glycans at multiple probe radii from 1.4 Å (water molecule) to 10 Å (protein-sized molecule). The values have been calculated and averaged across all replicas with glycans and are reported with standard deviation bars. The area shielded by the glycans is presented in yellow (rounded % values are reported), whereas the green line represents the accessible area of the protein in the absence of glycans. Highlighted in green is the area that remains accessible in the presence of glycans. The ASA for the TrkB dimer and monomer systems, which was calculated for probe radius 10 Å, is not shown because of incorrect calculation of the ASA in this high radius for the particular systems.

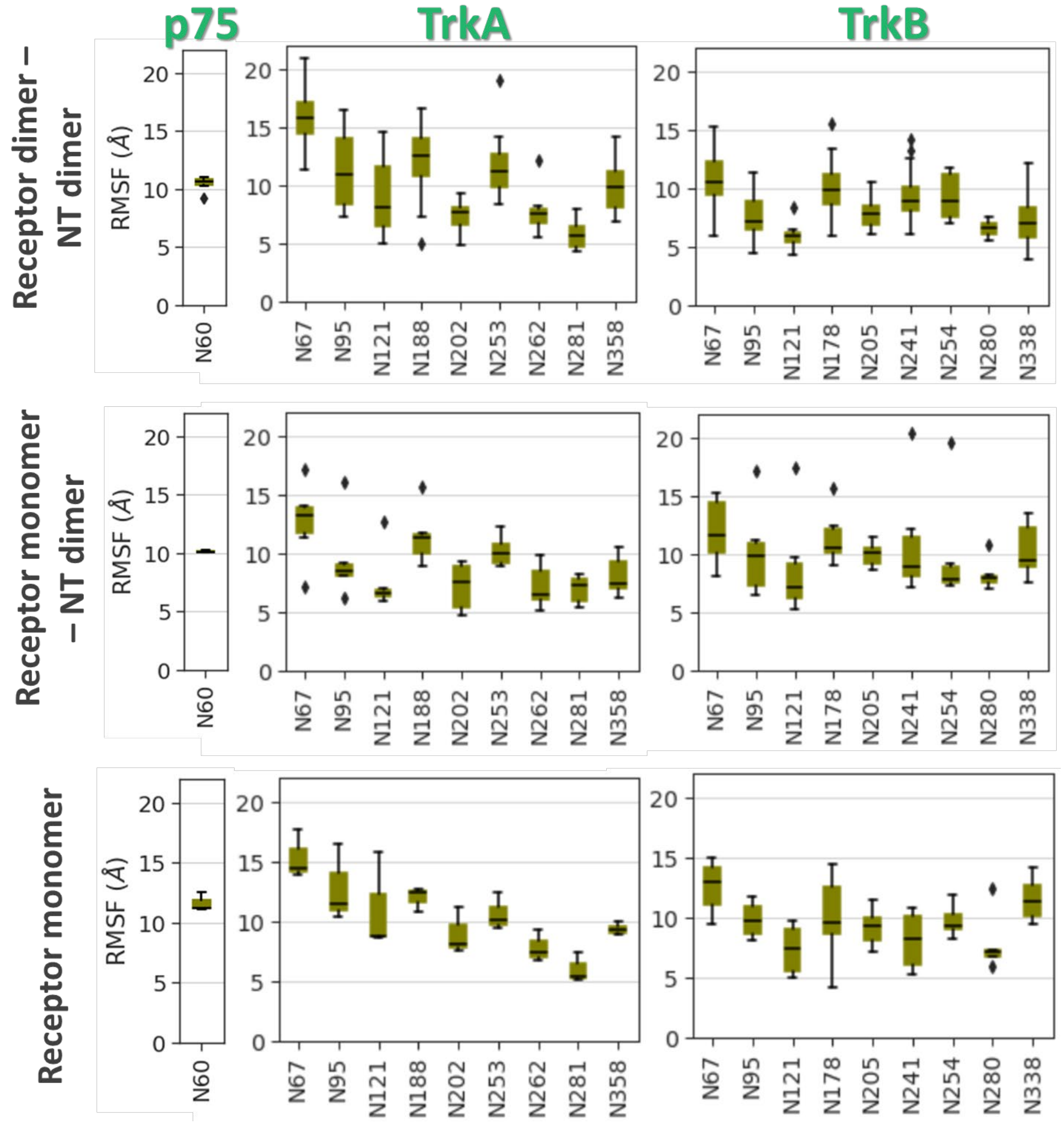

Figure S17: Box plots of RMSF calculations of all glycans attached in asparagine residues in all p75, TrkA and TrkB glycosylated systems. Before the RMSF calculation, alignment of each EC segment was performed to its initial structure (frame 0).

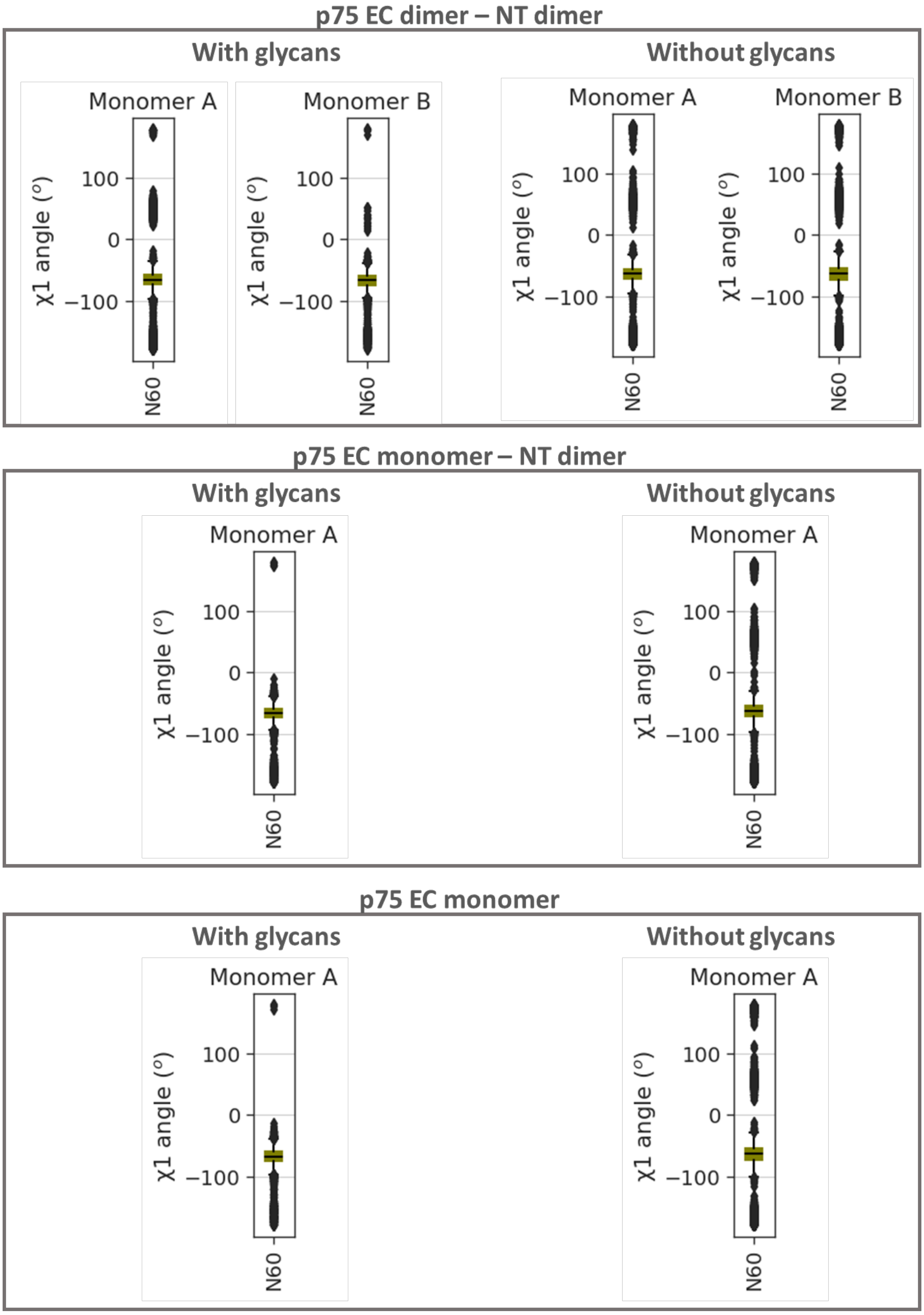

Figure S18: Distributions of the χ1 dihedral angle of N60 for the three p75-EC systems with and without the glycans present.

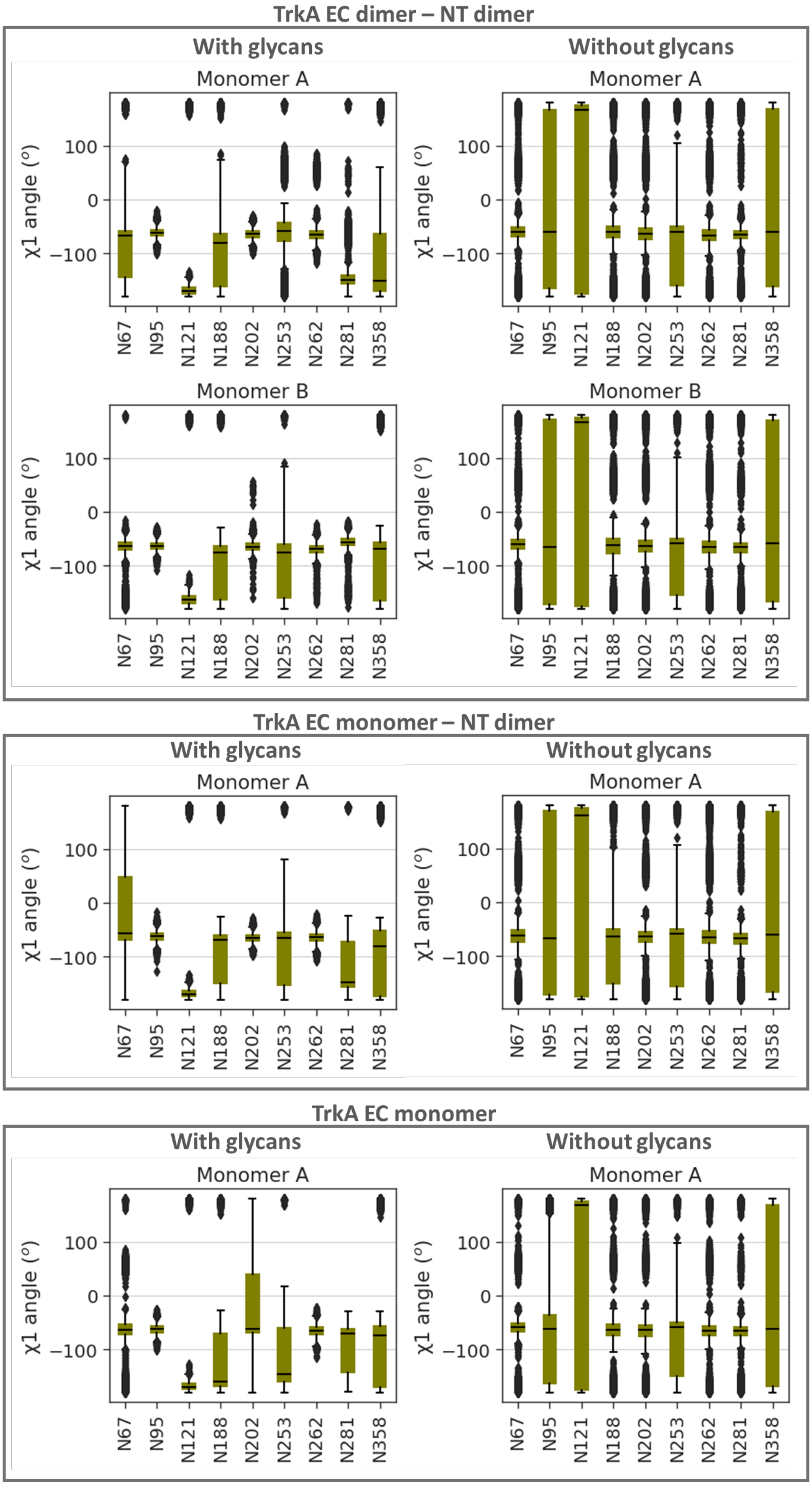

Figure S19: Box plots of the distribution of χ1 dihedral angle of glycosylated Asn residues. A comparison of the χ1 distributions is shown between glycosylated and non-glycosylated TrkA systems.

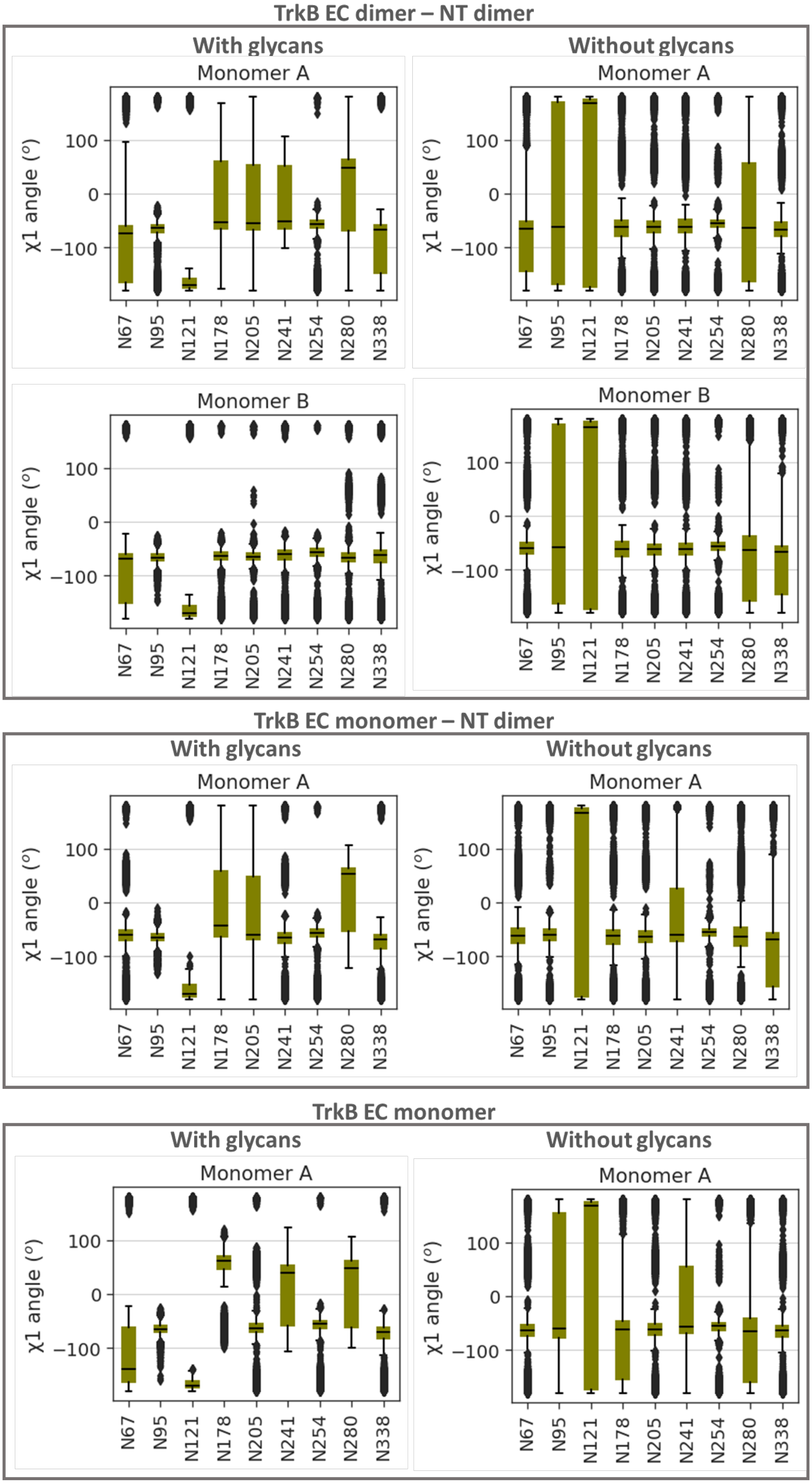

Figure S20: Box plots of the distribution of χ1 dihedral angle of glycosylated Asn residues. A comparison of the χ1 distributions is shown between glycosylated and non-glycosylated TrkB systems.

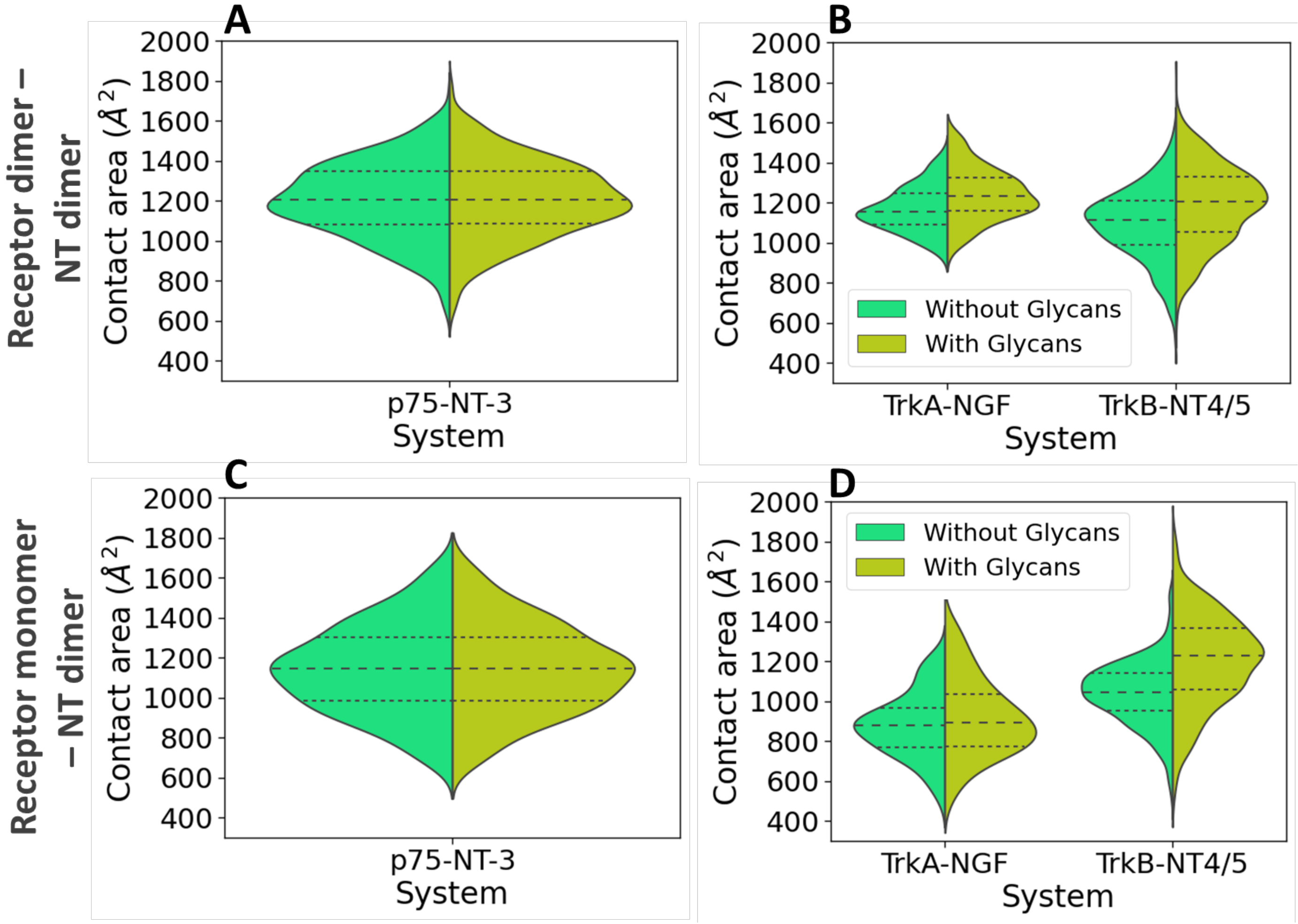

Figure S21: Violin plots with the contact area between the EC domains of p75, TrkA and TrkB receptors with the NTs. The systems with the EC receptor dimer bound to NTs with and without glycans are shown in (A) and (B), while the systems with the EC receptor monomers bound to NTs with and without glycans are shown in (C) and (D). For the systems with the EC dimers, the contact area for each monomer was calculated separately.

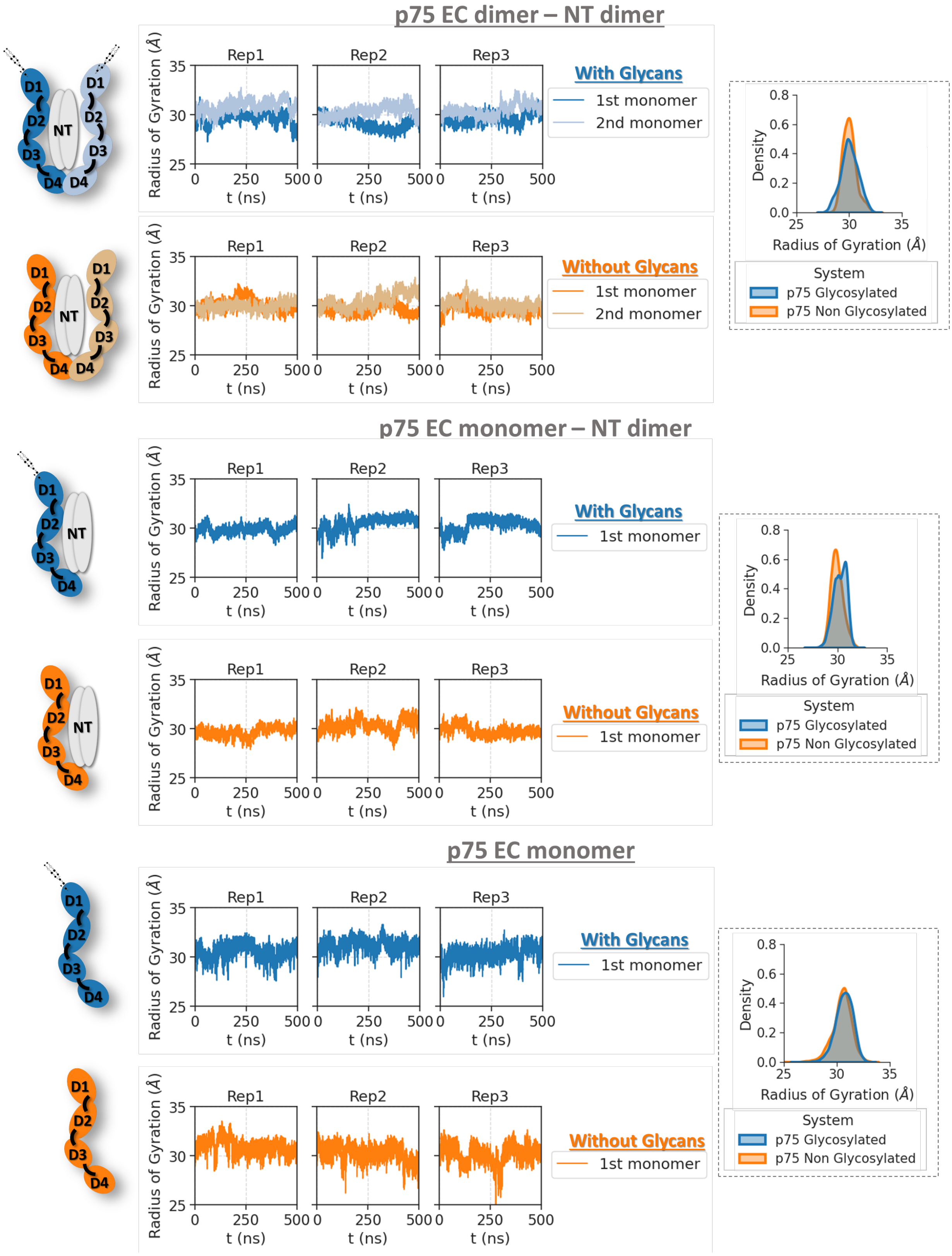

Figure S22: Time evolution and distribution of the radius of gyration values for the systems of p75-EC as a homodimer in complex to NT-3, as a monomer in complex with NT-3 and as a monomer alone, with (blue) and without (orange) glycans. For the system of p75-EC as a homodimer in complex to NT-3, the radius of gyration was calculated for both p75-EC domains separately.

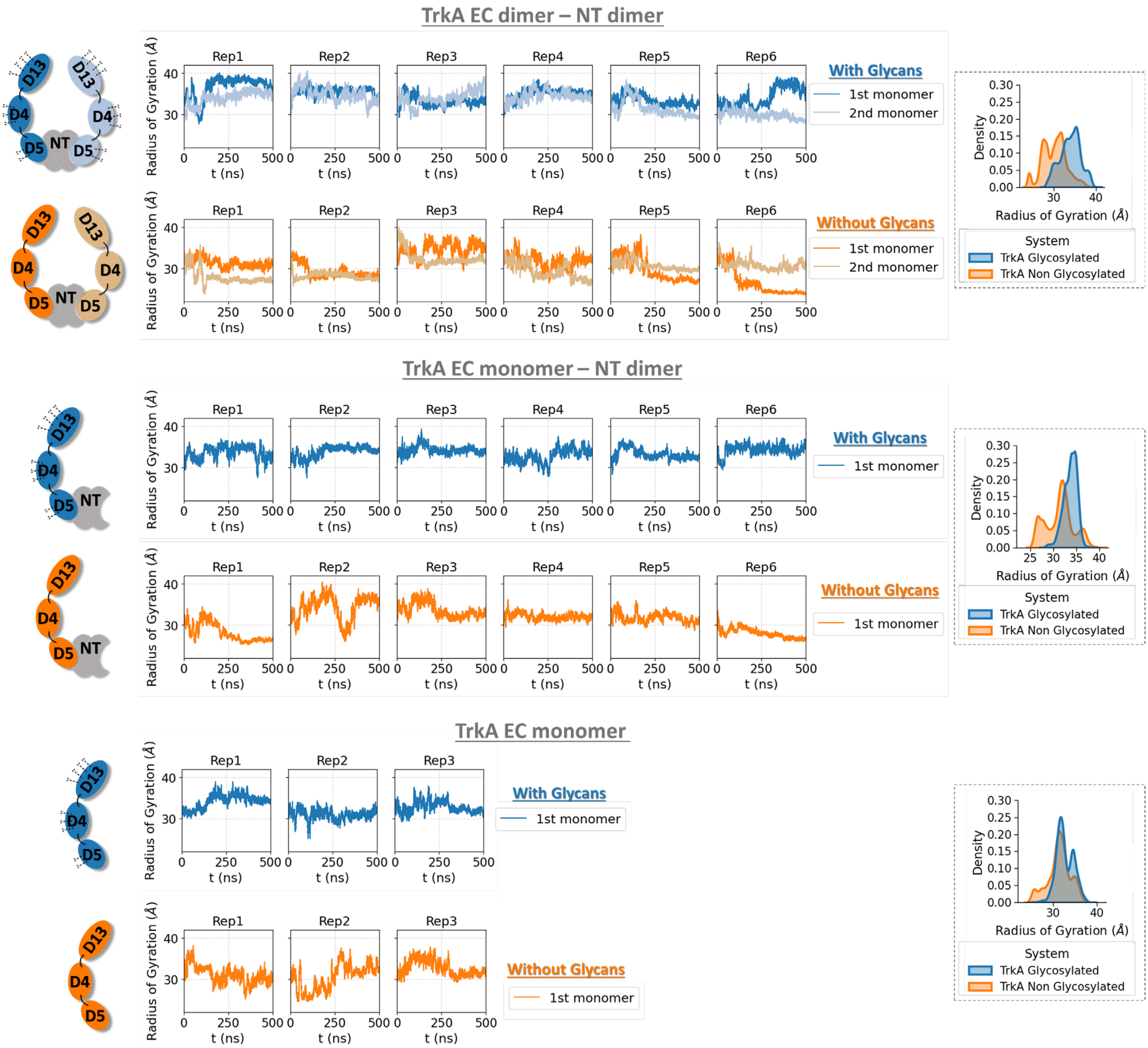

Figure S23: Radius of gyration of the receptor backbone of the glycosylated and not TrkA systems. The color-coding of each domain is shown in the schematic representations of the systems at the left of the plots. The time-evolution of the radius of each receptor monomer is shown for each replica, as well as the distributions of the radius of gyration values across all replicas for the glycosylated and non-glycosylated systems (right plots).

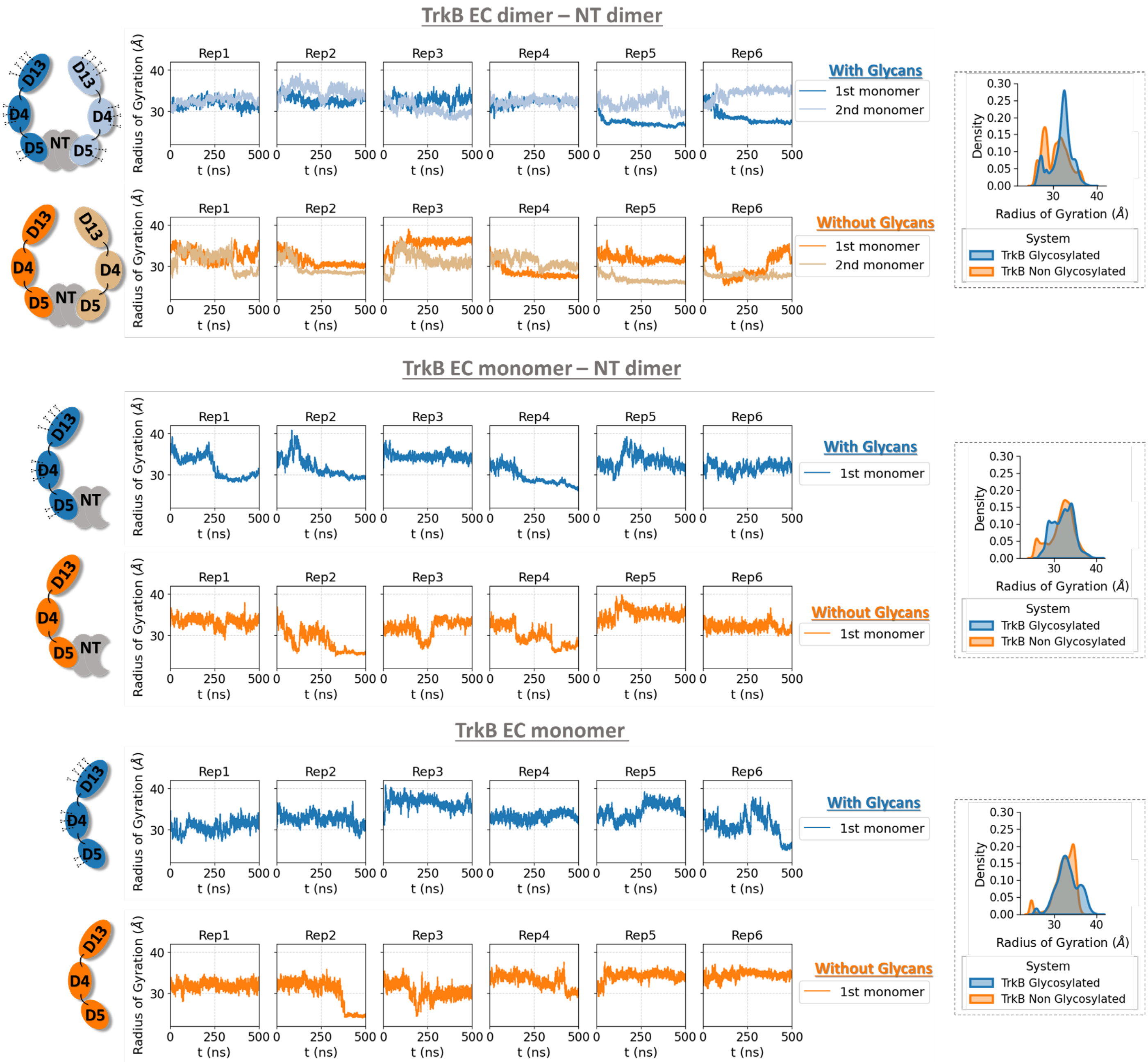

Figure S24: Radius of gyration of the receptor backbone of the glycosylated and not TrkB systems. The color-coding of each domain is shown in the schematic representations of the systems at the left of the plots. The time-evolution of the radius of each receptor monomer is shown for each replica, as well as the distributions of the radius of gyration values across all replicas for the glycosylated and non-glycosylated systems (right plots).

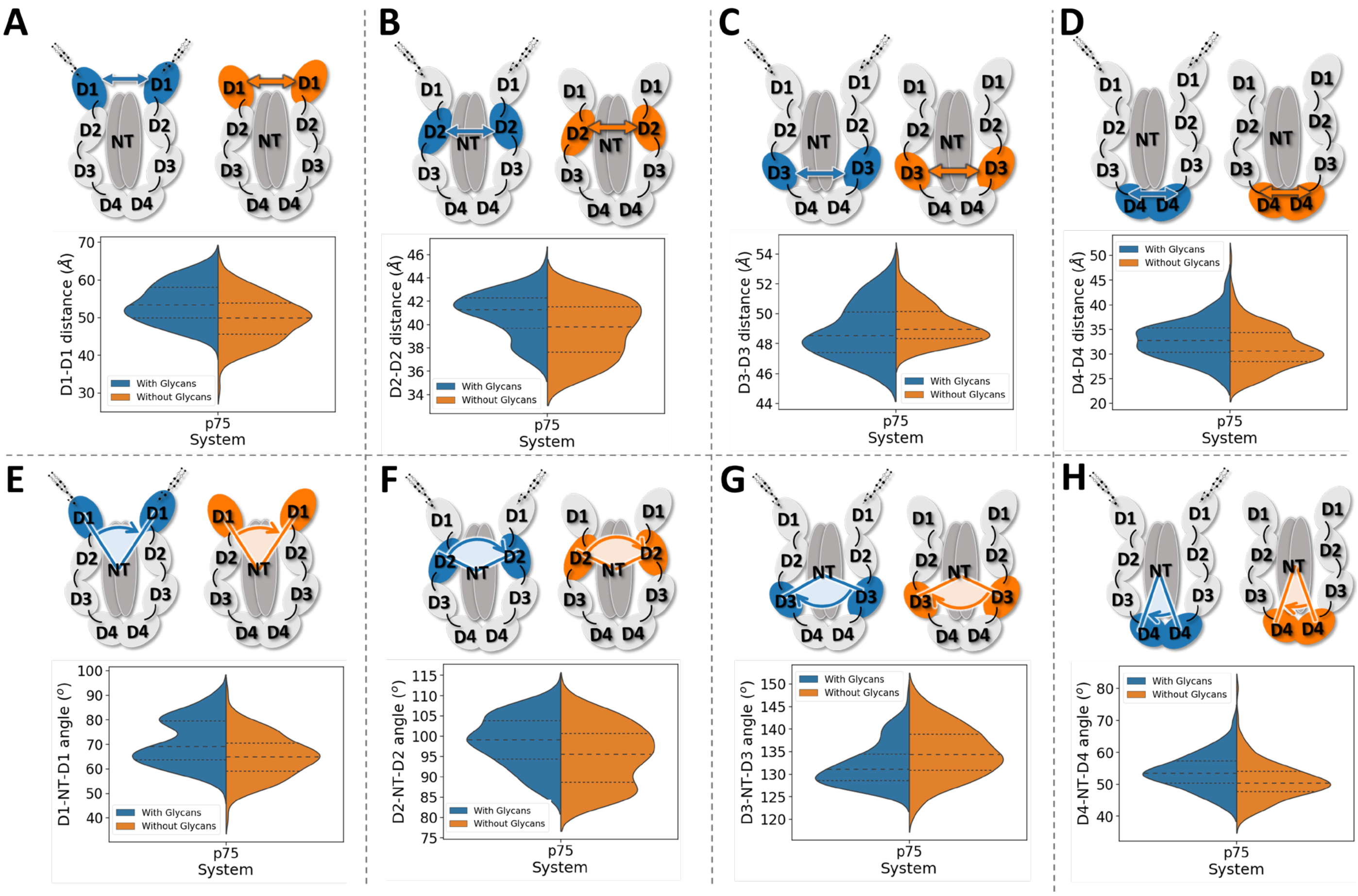

Figure S25: Calculated distributions of the distances and angles between the four p75-EC domains. (A)-(C) Distances between the COGs of D1-D1, D2-D2, D3-D3 and D4-D4 domains, in the presence (blue) and absence (orange) of glycans. (E)-(H) Angles defined by the COGs of D1-NT-D1, D2-NT-D2, D3-NT-D3 and D4-NT-D4 domains.

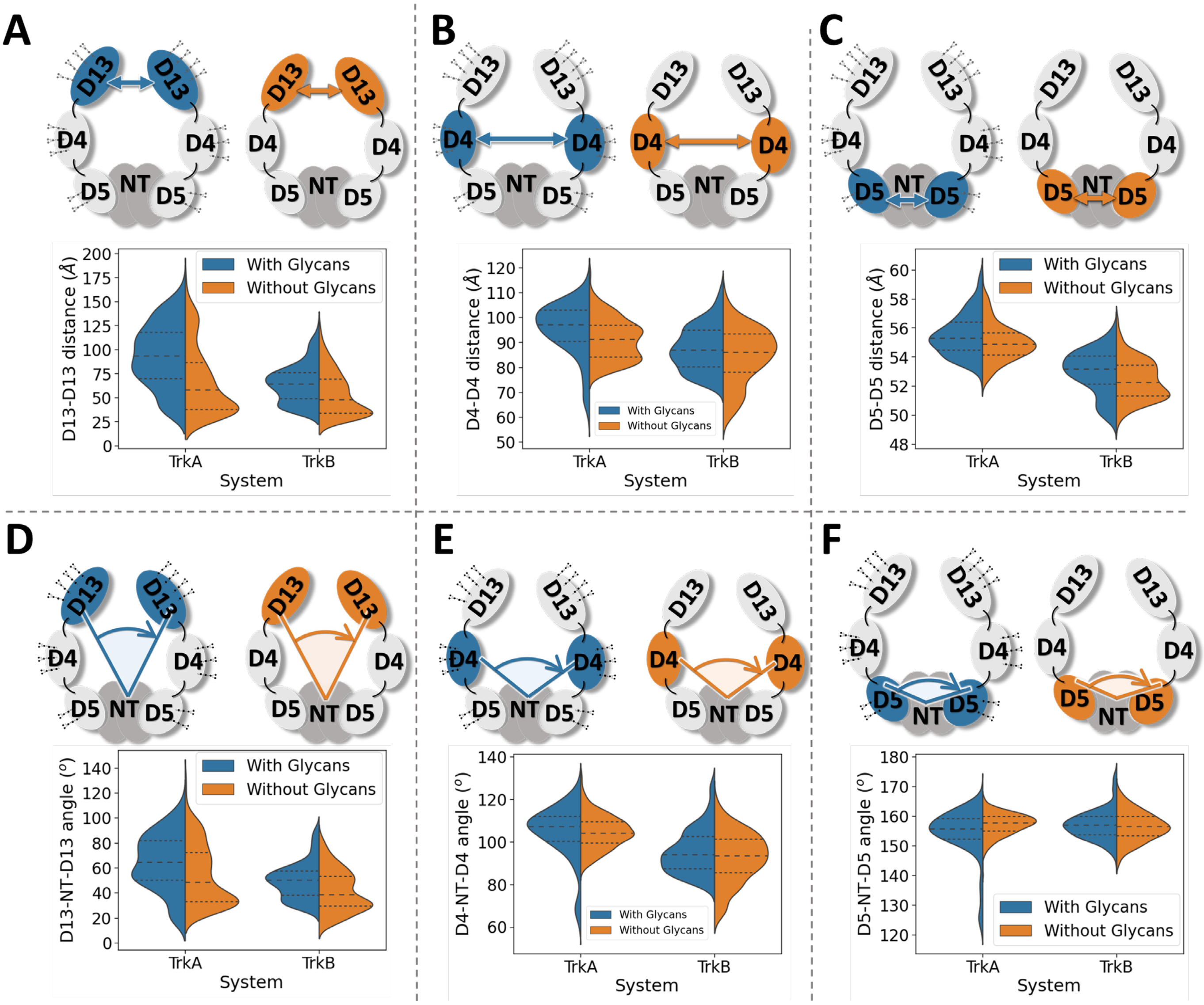

Figure S26: Distance and angle analysis between the receptor monomer domains. (A, B, C) Calculated distances between the centers of geometry (COG) of the D13, D4 and D5 domains from the two different receptor monomers. (D, E, F) Calculated angles formed between the COG of one of the same domains from the first receptor monomer, the COG of the NT and the COG of the same domain from the second monomer. In all cases, the distances and angles were calculated every 100 frames and the final distributions from the glycosylated and non-glycosylated TrkA and TrkB systems are shown in violin plots. The quartiles of the distributions are indicated with dashed lines.

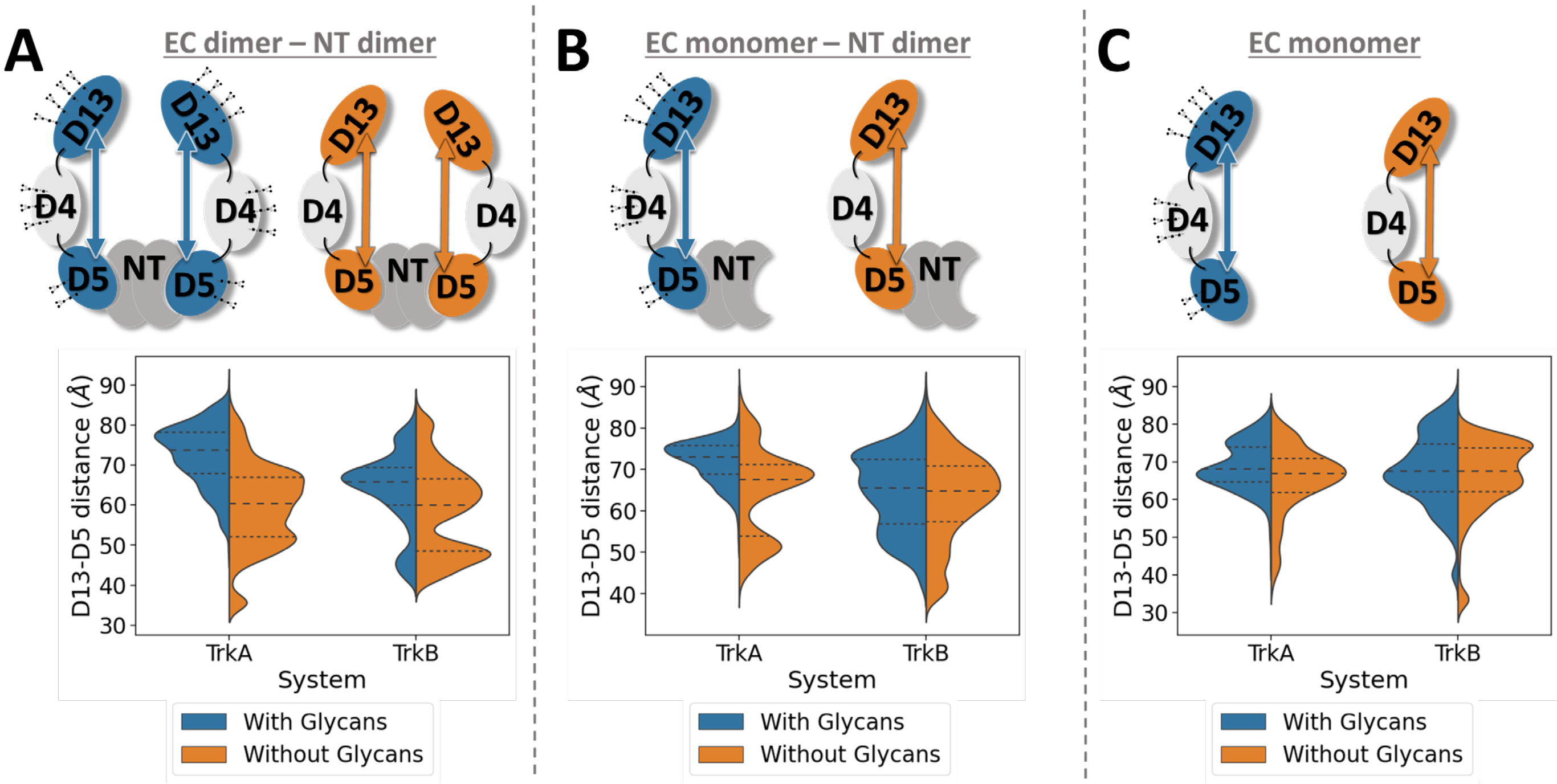

Figure S27: Distance between the centers of geometry of the D13-D5 domains in the different TrkA and TrkB systems (A, B, C). In all cases, the distances were calculated every 100 frames and the final distributions from the glycosylated and non-glycosylated TrkA and TrkB systems are shown in violin plots. The quartiles of the distributions are indicated with dashed lines.

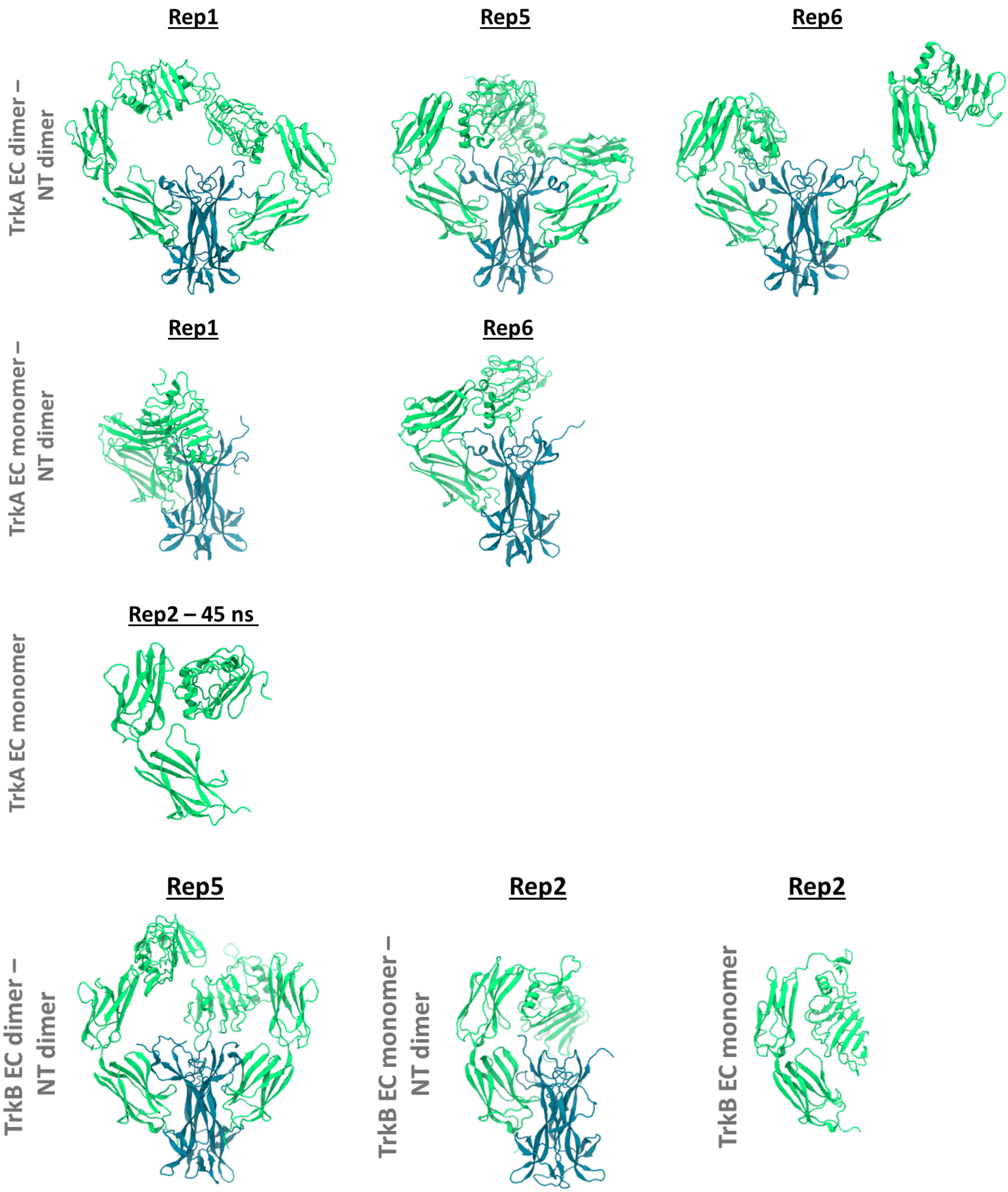

Figure S28: Conformations of the different non-glycosylated TrkA and TrkB systems, which correspond to low radius of gyration and inter-domain distance values. All the conformations are taken from the last frame of the indicated replica simulations, except for the last TrkA which is a rendering at 45 ns of the simulation.

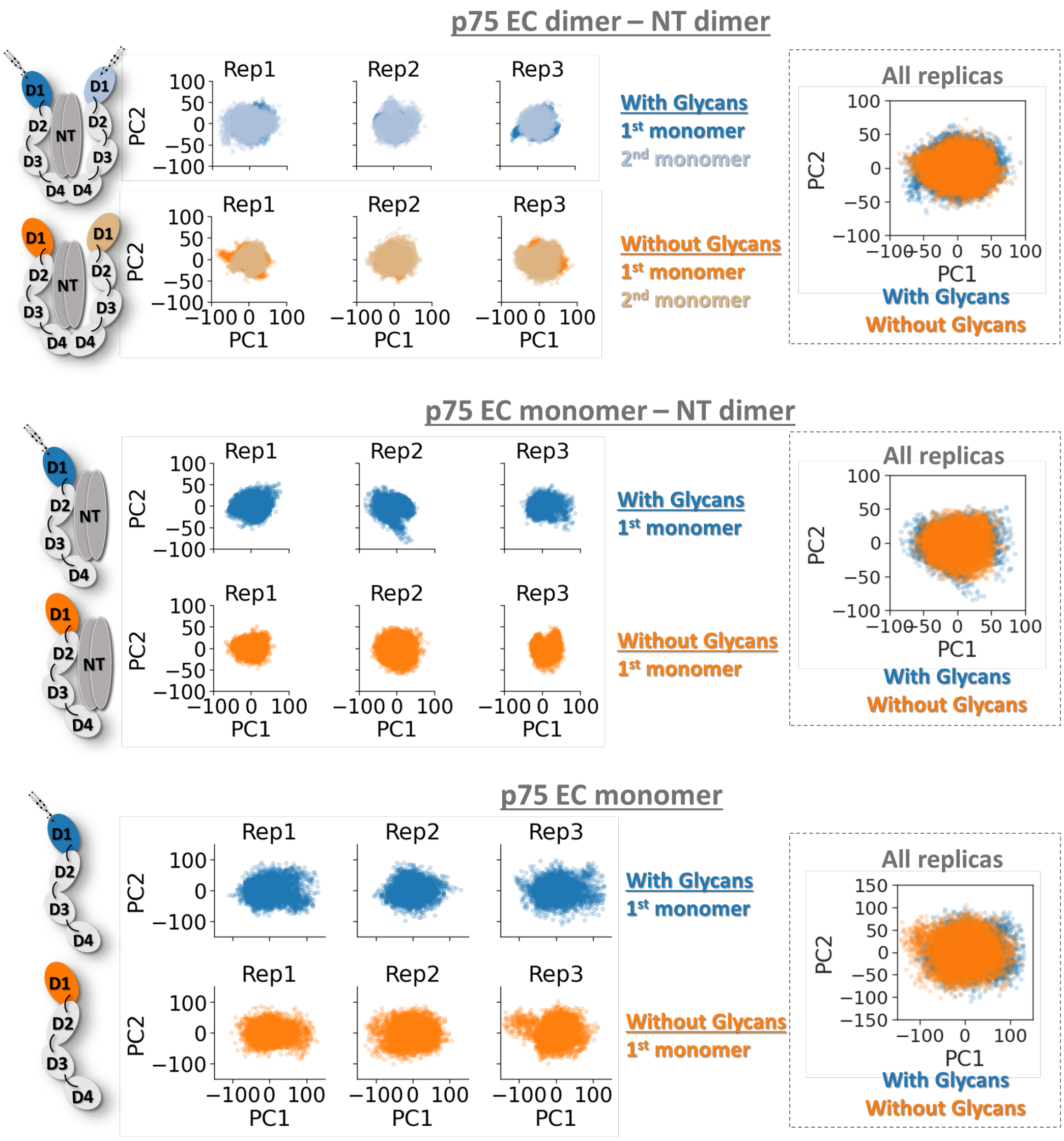

Figure S29: Principal component analysis (PCA) of the D1 domain of p75-EC after alignment to the rest of the domains for the glycosylated (blue) or not (orange) systems. Only the 1st and 2nd principal components are visualized.

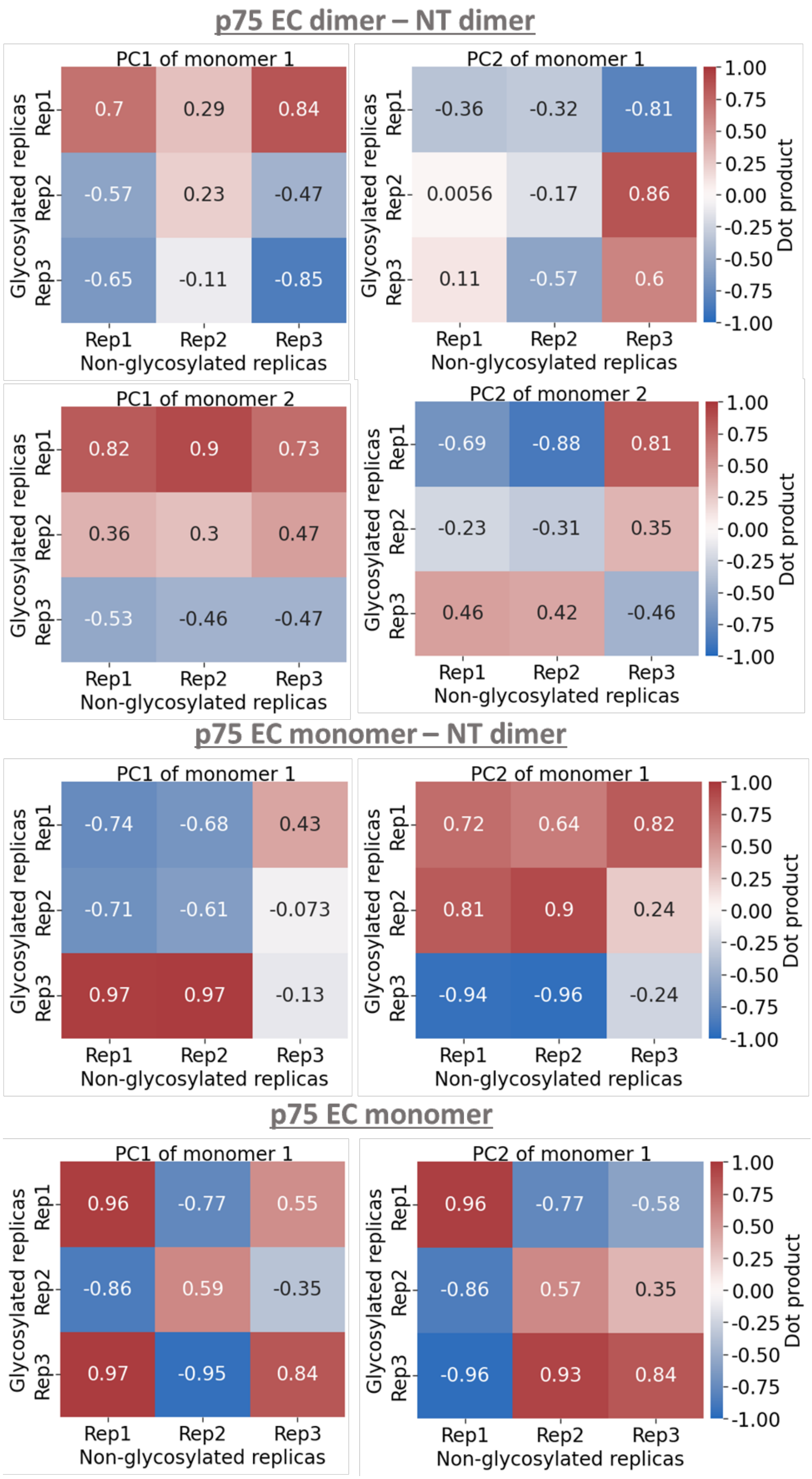

Figure S30: Dot products of the 1st and 2nd principal components from the various replicas and systems with and without glycans. The dot product values are also color-coded with heatmaps, with values equal to 1 showing parallel vectors, values of -1 showing antiparallel vector and 0 meaning vertical vectors. PCA analysis was performed separately for each replica simulation. As a result, the PC1 and PC2 are not necessarily the same in all replica simulations. Thus, to compare the PCs between different replicas, the dot products between the PC1s and PC2s from the various replicas were calculated. The results showed that in some cases the PCs were pointing towards the same direction (positive dot products) between the glycosylated and non-glycosylated systems, while in other cases they pointed in opposite directions (negative dot products), or were even perpendicular to each other (close to zero dot products).

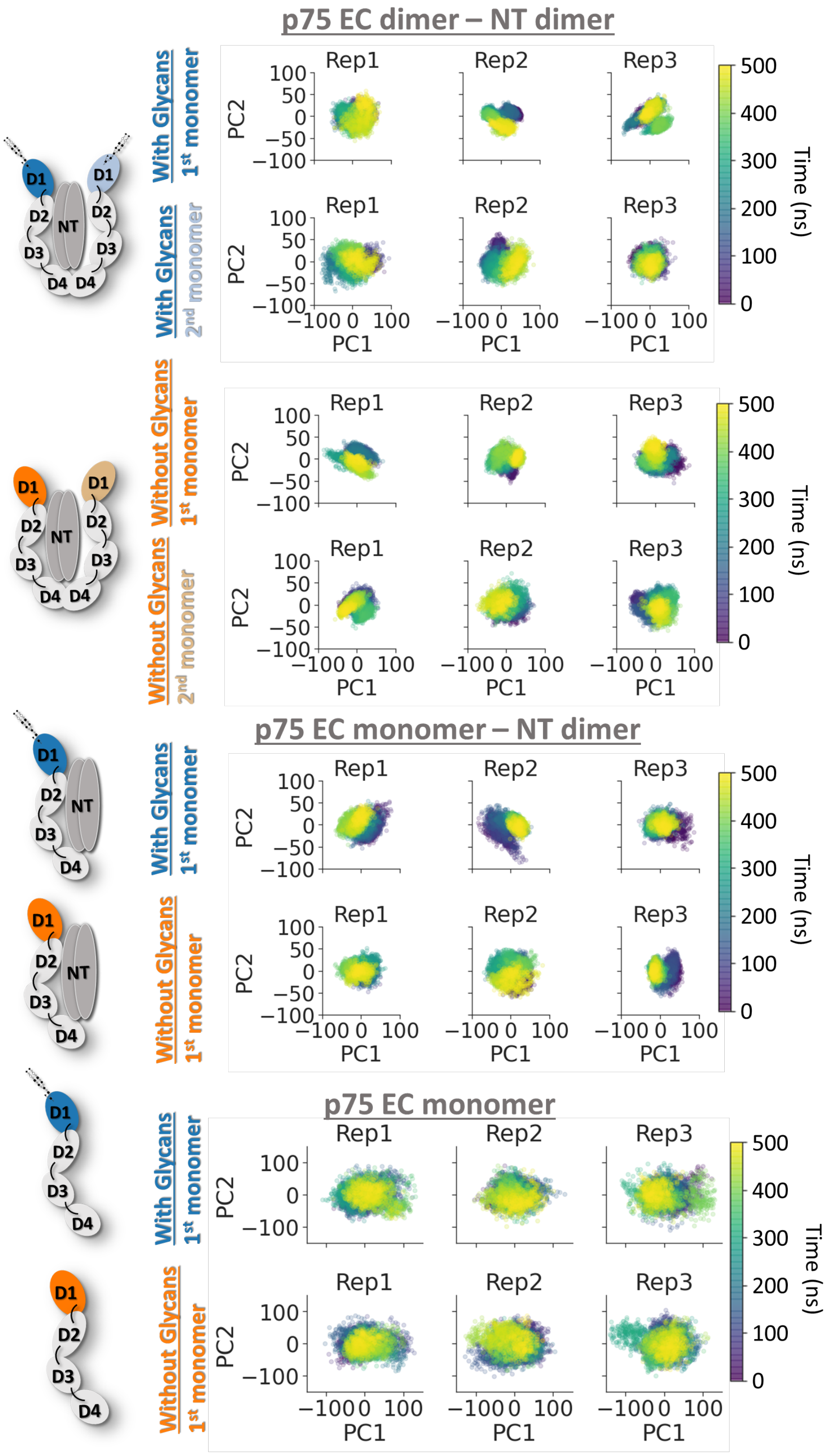

Figure S31: Principal component analysis (PCA) of the D1 domain of p75-EC as a time evolution after alignment to the rest of the domains, for the glycosylated or not systems. Only the 1st and 2nd principal components are visualized. The systems visit the same PC1-PC2 space at all times of the simulation, which is probably facilitated by the narrow space that is explored by the system, consistent with the previously discussed rigidity of the p75-EC domain.

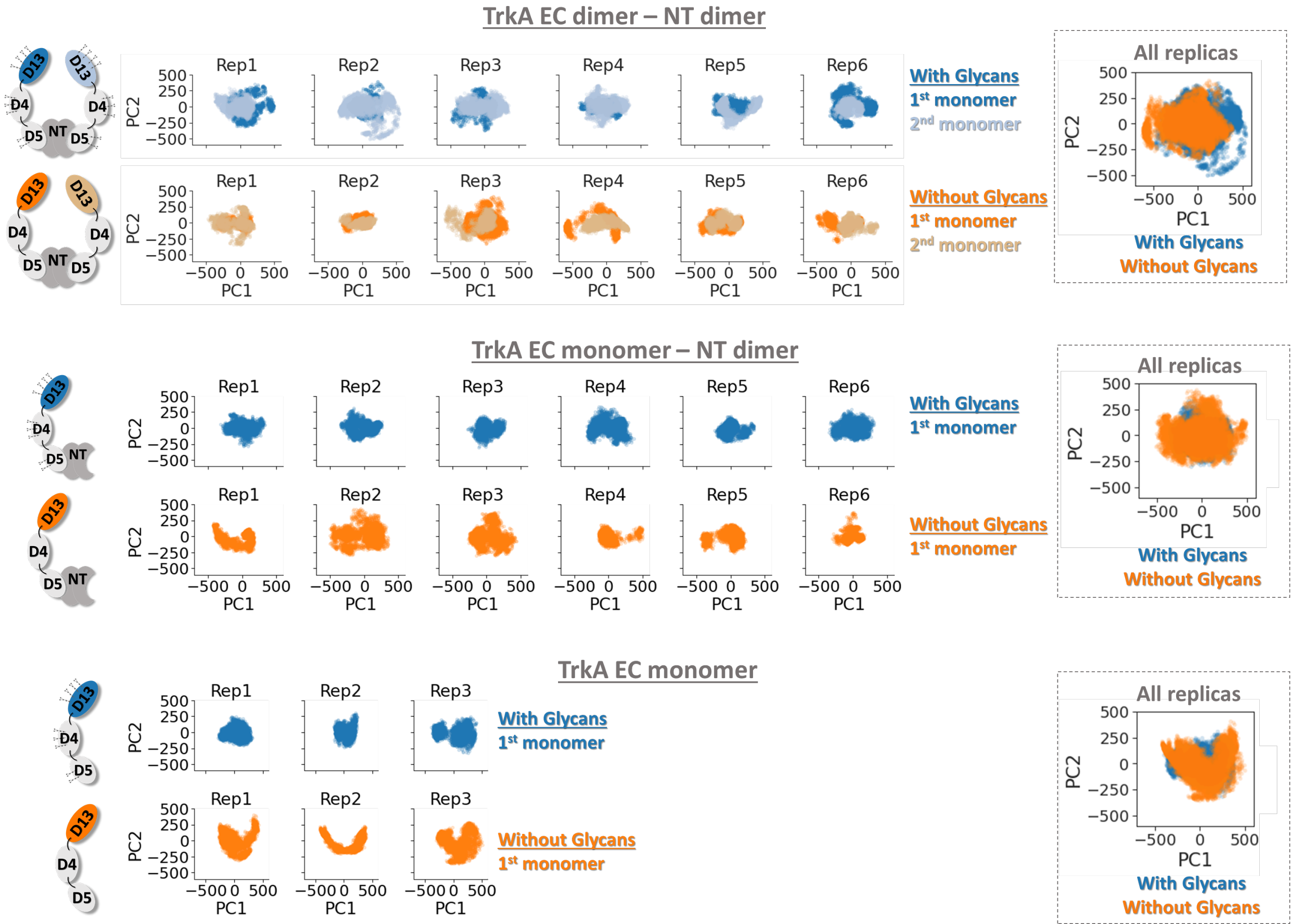

Figure S32: First and second principal components of the D13 domain during the simulations of the glycosylated and not TrkA systems. PCA analysis was performed to the D13 domains after alignment of each EC segment to the D4 and D5 domains. The color coding of each domain is shown in the schematic representations of the systems at the left of the plots.

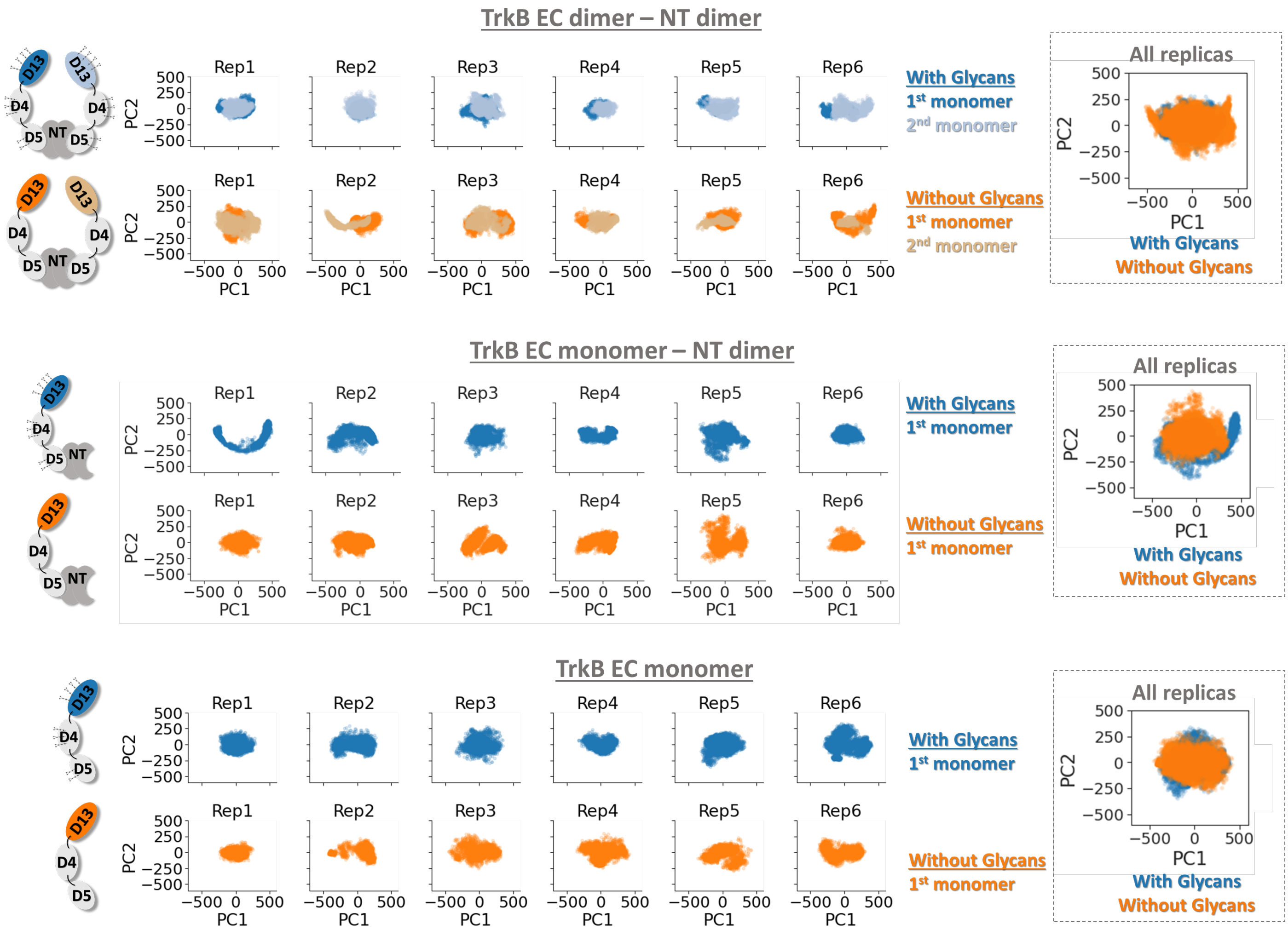

Figure S33: First and second principal components of the D13 domain during the simulations of the glycosylated and not TrkB systems. PCA analysis was performed to the D13 domains after alignment of each EC segment to the D4 and D5 domains. The color coding of each domain is shown in the schematic representations of the systems at the left of the plots.

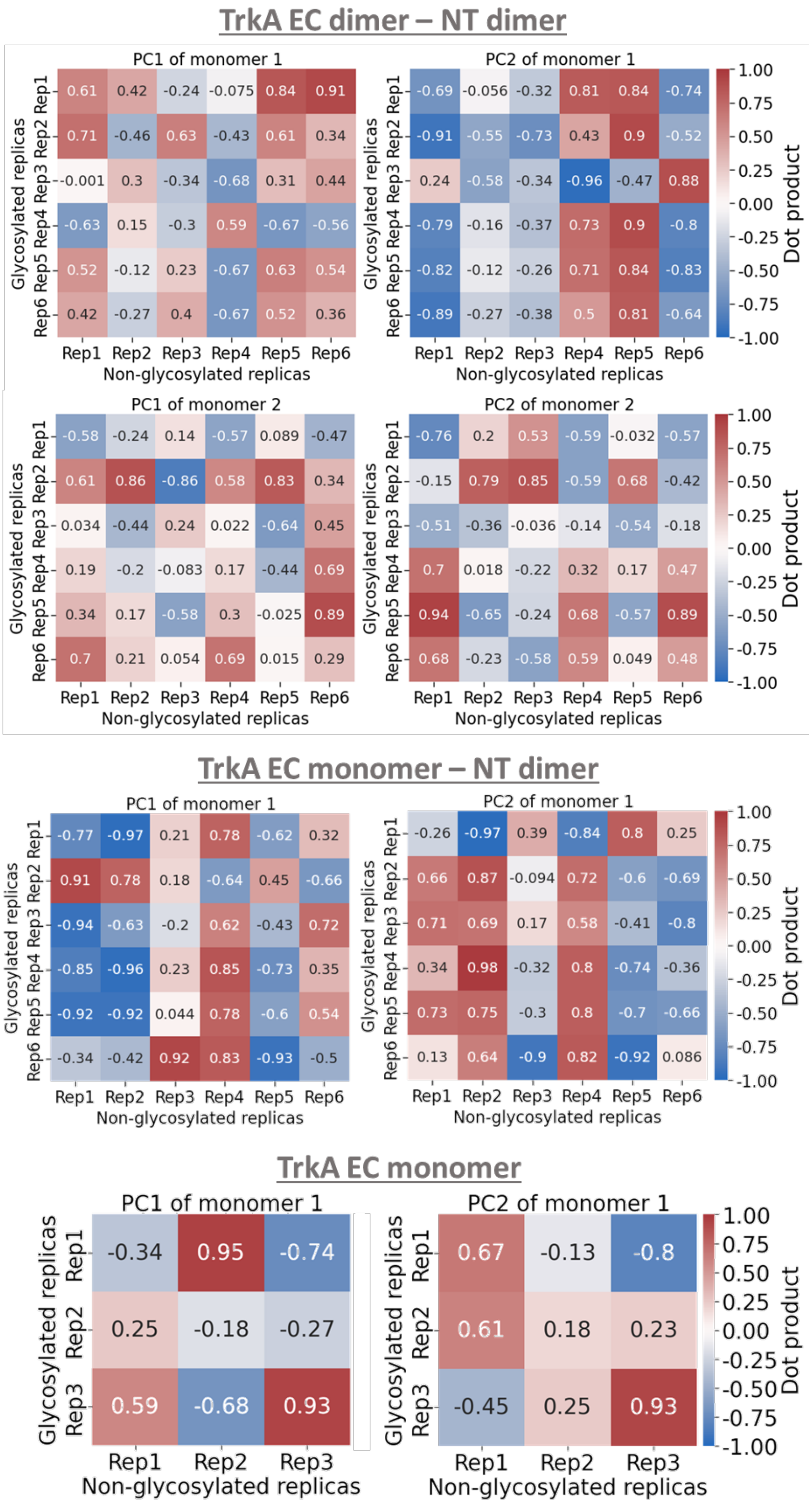

Figure S34: Calculation of the dot products of the first (PC1) and second (PC2) principal components between glycosylated and non-glycosylated replica pairs of TrkA systems. The PCA was performed on the D13 domain only, after alignment of the trajectories for the D4-D5 domains. In the systems of the receptor dimers, the PC1 and PC2 dot products were calculated for each monomer separately. The dot products are visualized as heatmaps, and the values mean: [1] parallel vectors, [-1] anti-parallel vectors and [0] vertical (or orthogonal) vectors. The analysis showes values close to zero for TrkA, indicating not so similar motions.

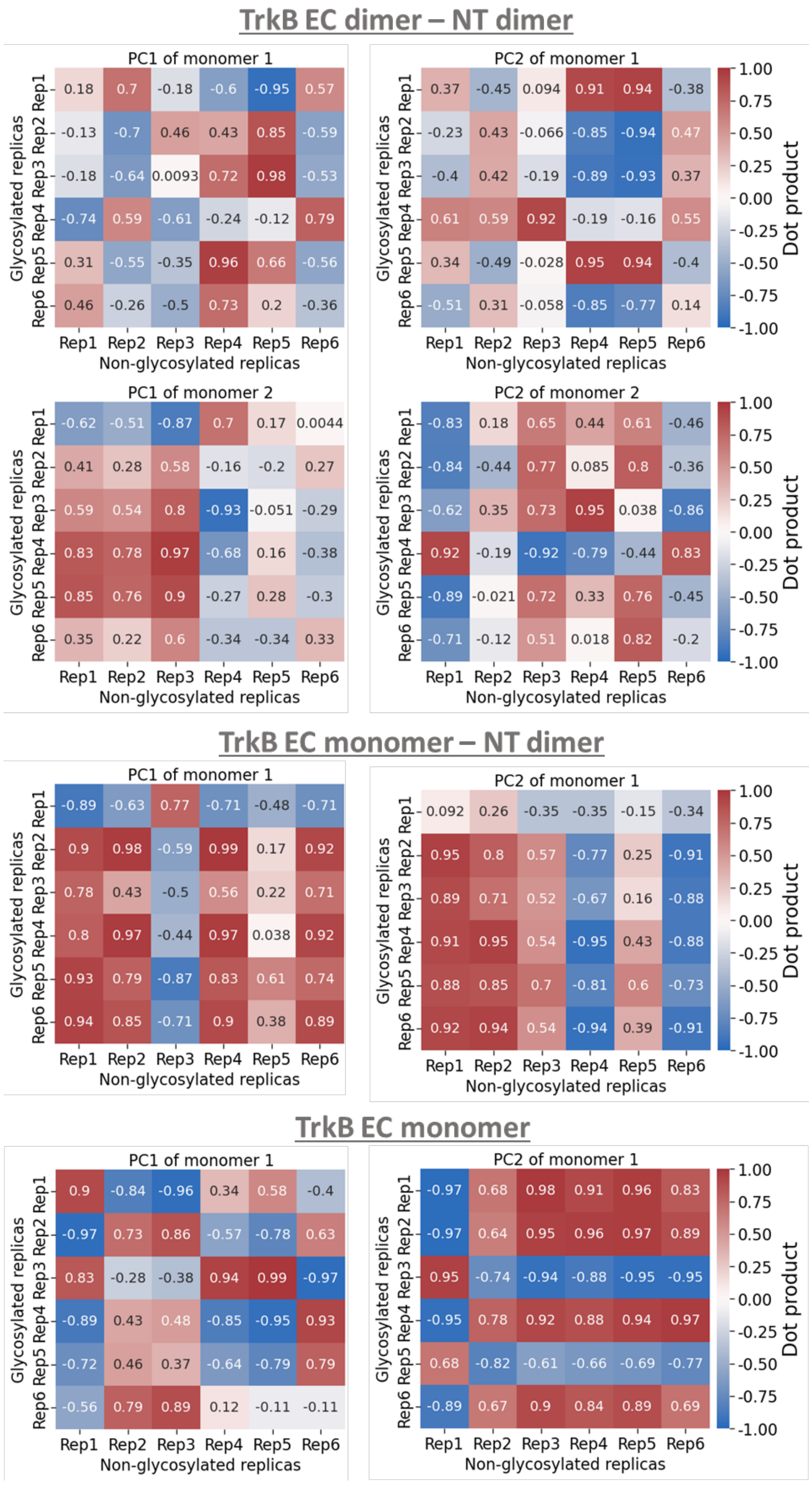

Figure S35: Calculation of the dot products of the first (PC1) and second (PC2) principal components between glycosylated and non-glycosylated replica pairs of TrkB systems. The PCA was performed on the D13 domain only, after alignment of the trajectories in the D4-D5 domains. In the systems of the receptor dimers, the PC1 and PC2 dot products were calculated for each monomer separately. The dot products are visualized as heatmaps, and the values mean: [1] parallel vectors, [-1] anti-parallel vectors and [0] vertical (or orthogonal) vectors. In contrast to TrkA, for TrkB, stronger similarities were observed with parallel or anti-parallel principal components, especially for the systems with receptor monomers. This behavior seems to coincide with the bigger differences observed between glycosylated and non-glycosylated forms for the TrkA systems compared to TrkB.

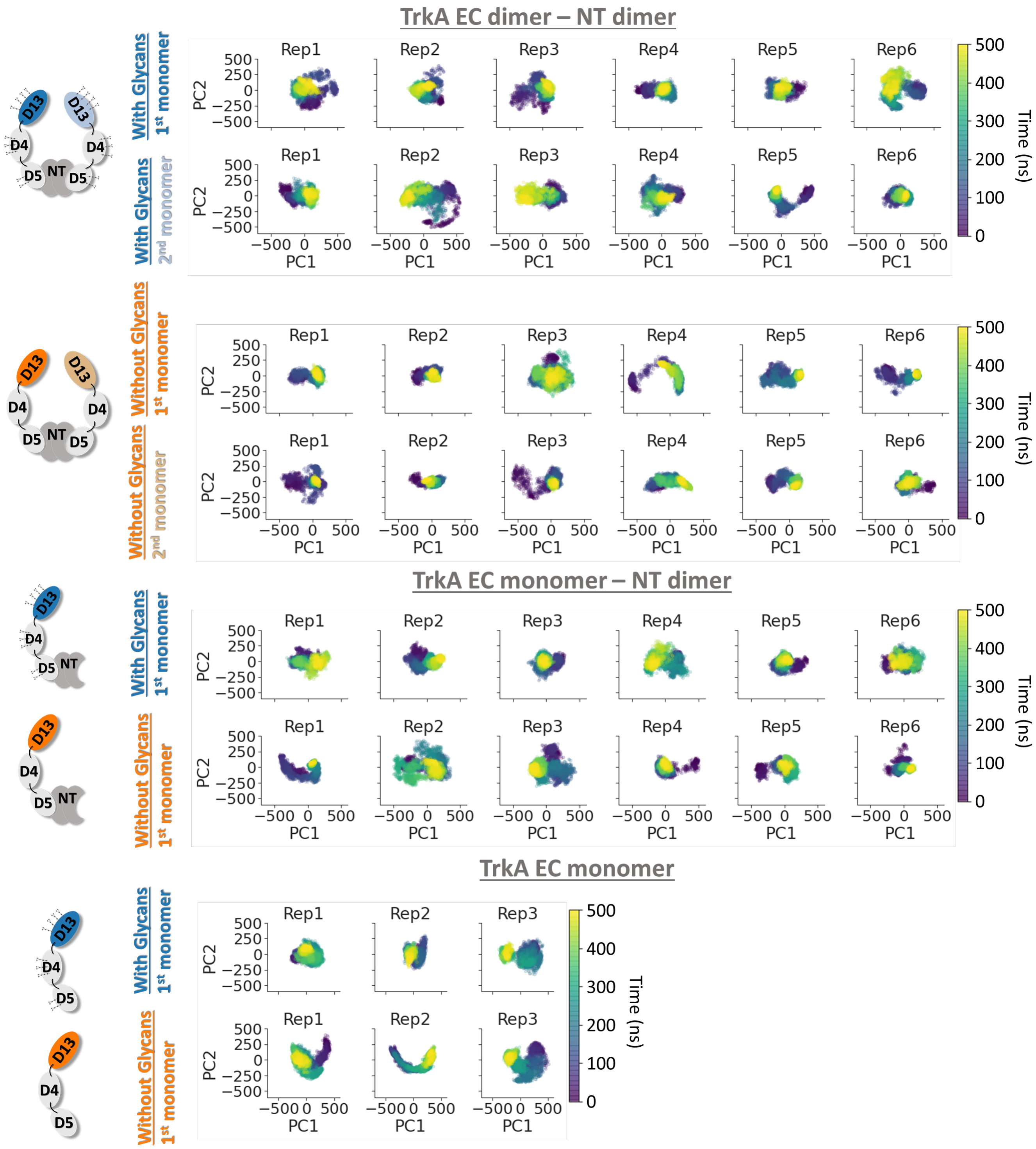

Figure S36: Time evolution of the first and second principal components of the D13 domain during the simulations of the glycosylated and not TrkA systems. PCA analysis was performed to the D13 domains after alignment of each EC segment to the D4 and D5 domains. The color coding of each domain is shown in the schematic representations of the systems at the left of the plots. The systems explore specific regions of the PC1 and PC2 space. In cases where some conformational change takes place during the simulations, and thus the system moves from one region to another in the PC1/PC2 space, no revisiting of the initial region takes place.

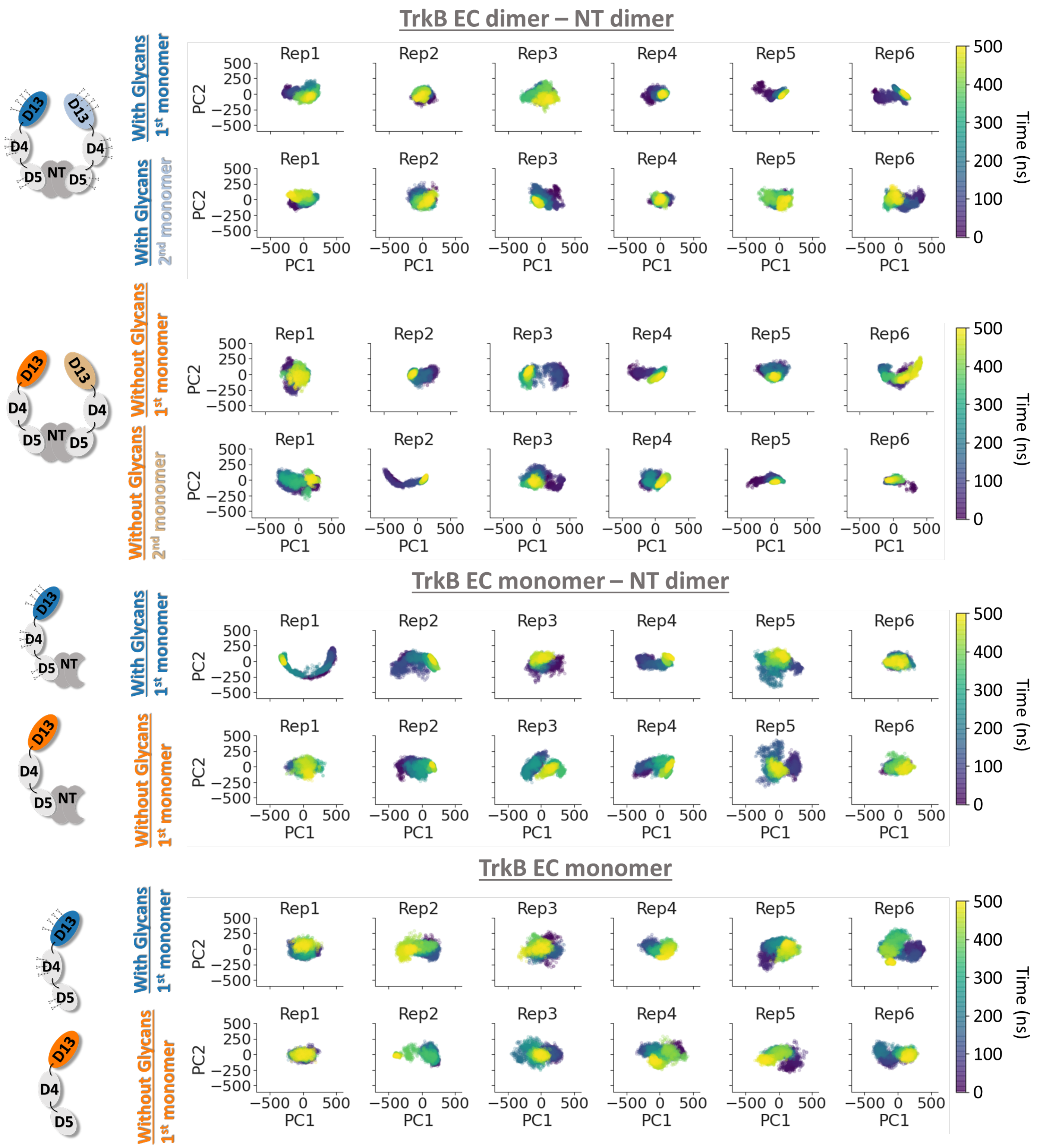

Figure S37: Time evolution of the first and second principal components of the D13 domain during the simulations of the glycosylated and not TrkB systems. PCA analysis was performed to the D13 domains after alignment of each EC segment to the D4 and D5 domains. The color coding of each domain is shown in the schematic representations of the systems at the left of the plots. The systems explore specific regions of the PC1 and PC2 space. In cases where some conformational change takes place during the simulations, and thus the system moves from one region to another in the PC1/PC2 space, no revisiting of the initial region takes place.

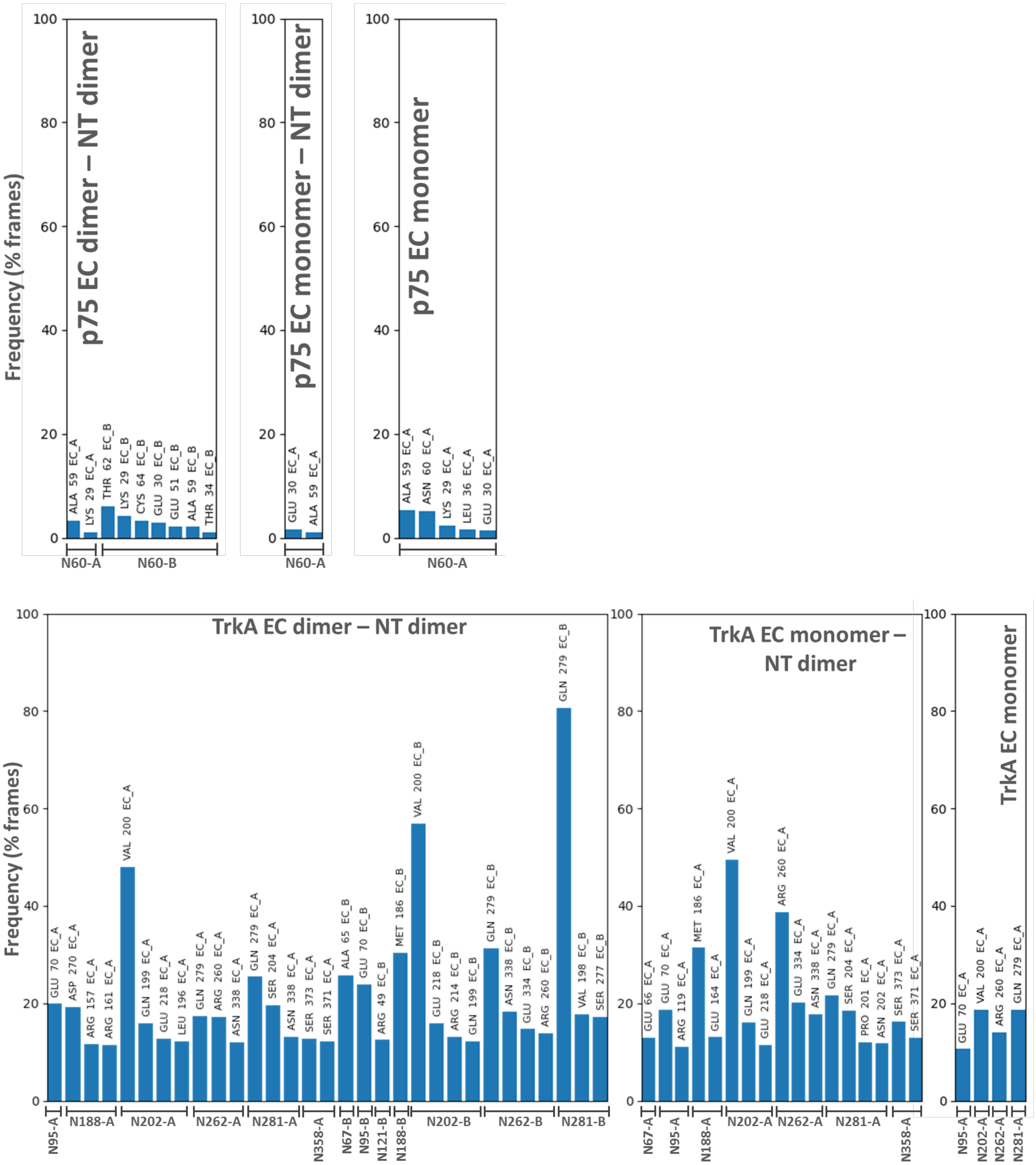
p75 EC dimer – NT dimer
p75 EC monomer – NT dimer
p75 EC monomer
Frequency (% frames)
ALA 59 EC_A
LYS 29 EC_A
THR 62 EC_B
LYS 29 EC_B
CYS 64 EC_B
GLU 30 EC_B
GLU 51 EC_B
ALA 59 EC_B
THR 34 EC_B
N60-A
N60-B
GLU 30 EC_A
ALA 59 EC_A
N60-A
ALA 59 EC_A
ASN 60 EC_A
LYS 29 EC_A
LEU 36 EC_A
GLU 30 EC_A
N60-A
TrkA EC dimer – NT dimer
TrkA EC monomer – NT dimer
TrkA EC monomer
GLU 70 EC_A
ASP 270 EC_A
ARG 157 EC_A
ARG 161 EC_A
VAL 200 EC_A
GLN 199 EC_A
GLU 218 EC_A
LEU 196 EC_A
GLN 279 EC_A
ARG 260 EC_A
ASN 338 EC_A
GLN 279 EC_A
SER 204 EC_A
ASN 338 EC_A
SER 373 EC_A
SER 371 EC_A
ALA 65 EC_B
GLU 70 EC_B
ARG 49 EC_B
MET 186 EC_B
VAL 200 EC_B
GLU 218 EC_B
ARG 214 EC_B
GLN 199 EC_B
GLN 279 EC_B
ASN 338 EC_B
GLU 334 EC_B
ARG 260 EC_B
GLN 279 EC_B
VAL 198 EC_B
SER 277 EC_B
N95-A
N188-A
N202-A
N262-A
N281-A
N358-A
N67-B
N95-B
N121-B
N188-B
N202-B
N262-B
N281-B
GLU 66 EC_A
GLU 70 EC_A
ARG 119 EC_A
MET 186 EC_A
GLU 164 EC_A
VAL 200 EC_A
GLN 199 EC_A
GLU 218 EC_A
ARG 260 EC_A
GLU 334 EC_A
ASN 338 EC_A
GLN 279 EC_A
SER 204 EC_A
PRO 201 EC_A
ASN 202 EC_A
SER 373 EC_A
SER 371 EC_A
N67-A
N95-A
N188-A
N202-A
N262-A
N281-A
N358-A
GLU 70 EC_A
VAL 200 EC_A
ARG 260 EC_A
GLN 279 EC_A
N95-A
N202-A
N262-A
N281-A

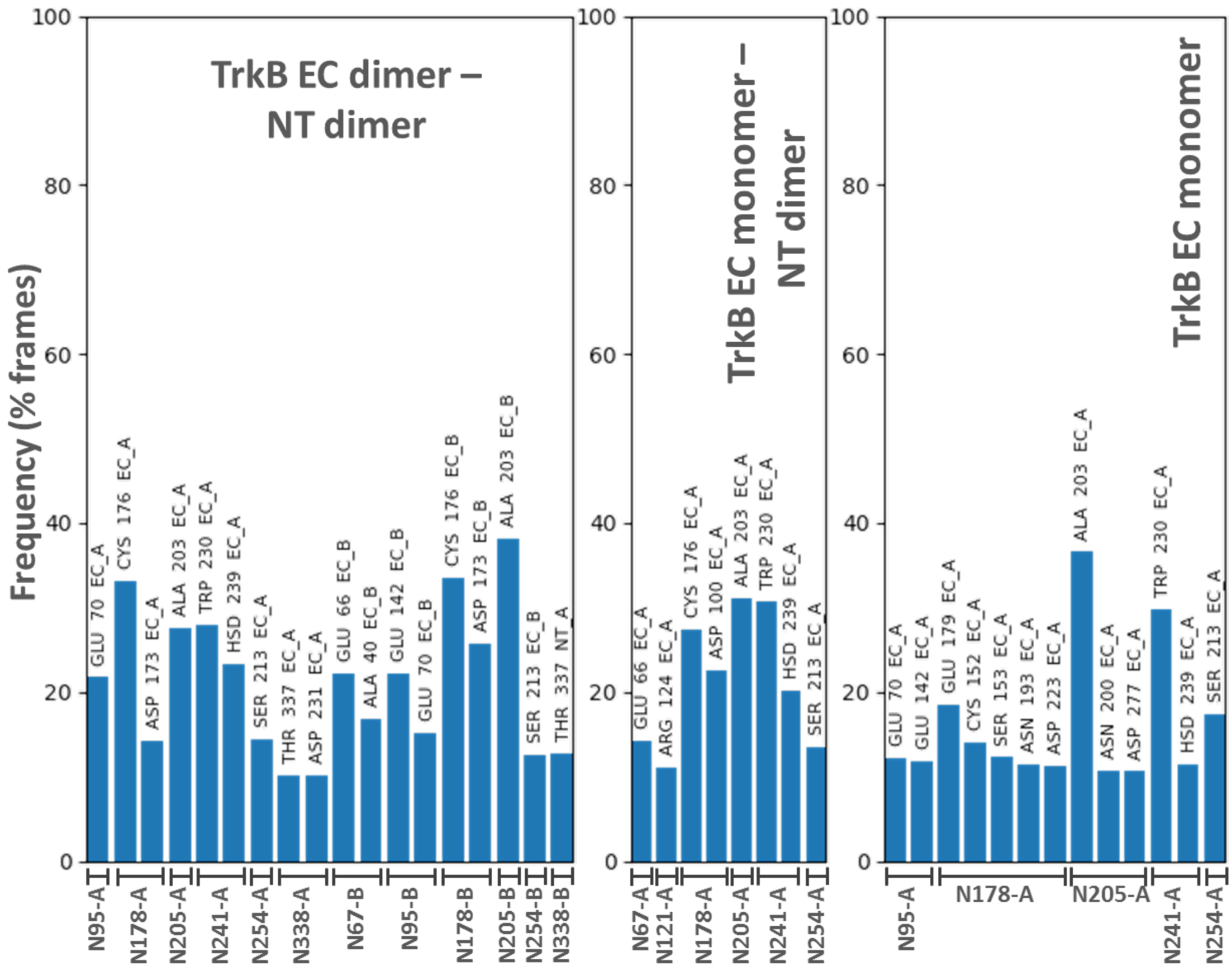

Figure S38: Hydrogen bonding analysis between the glycans attached in different Asn residues and the protein residues from all glycosylated p75, TrkA and TrkB systems. The glycans are indicated with the Asn residues from each receptor monomer (with A and B) in the x-axis, while the protein residues that interact with the glycans are indicated at the top of each bar. The height of the bars shows the percentage of the total simulation time in which the corresponding hydrogen bonding is present. Only hydrogen bonding of higher than 1% occupancy for p75, and 10% occupancy for the Trks is shown.

Supporting References

1. Haniu, M. *et al.* Extracellular Domain of Neurotrophin Receptor trkB: Disulfide Structure, N-Glycosylation Sites, and Ligand Binding. *Arch. Biochem. Biophys.* **322**, 256–264 (1995).
2. Haniu, M. *et al.* Interactions between Brain-derived Neurotrophic Factor and the TRKB Receptor. *J. Biol. Chem.* **272**, 25296–25303 (1997).
3. Wehrman, T. *et al.* Structural and Mechanistic Insights into Nerve Growth Factor Interactions with the TrkA and p75 Receptors. *Neuron* **53**, 25–38 (2007).
